# Inhomogeneity of negative enthalpy enables excellent mechanical properties in nanosized single crystals of multi-principal element alloys

**DOI:** 10.1038/s41467-026-78017-7

**Published:** 2026-10-11

**Authors:** Yan Ma, Zhanxin Wang, Jiabao Zhang, Ning Xu, Zhimeng Duan, Dongwei Li, Ziqi Wu, Xiaoxue Chang, Ruiwen Shao, Tingyang Huang, Xiayan Wang, Zaicheng Sun, Changtian Wang, Zibing An, Chengpeng Yang, Jianfei Zhang, Xiaozhou Liao, Lihua Wang, Xiaodong Han

**Affiliations:** 1 Beijing Key Lab of Microstructure and Property of Advanced Materials, College of Materials Science & Engineering, Beijing University of Technology, Beijing, China; 2 Branch of Iron and Steel Research Institute, Ansteel Beijing Research Institute Co., Ltd., Beijing, China; 3 School of Medical Science and Engineering, Beijing Institute of Technology, Beijing, China; 4 Analysis & Testing Center, Beijing Institute of Technology, Beijing, China; 5 School of Economics, Beijing Wuzi University, Beijing, China; 6 State Key Laboratory of Materials Low-Carbon Recycling, College of Chemistry and Life Science, Beijing University of Technology, Beijing, China; 7 Department of Materials Science and Engineering, Southern University of Science and Technology, Shenzhen, China; 8 Key Laboratory of Multifunctional Nanomaterials and Smart Systems, Division of Advanced Materials, Suzhou Institute of Nano-Tech and Nano-Bionics, Chinese Academy of Sciences, Suzhou, China; 9 Beijing Key Laboratory of Multimedia and Intelligent Software Technology, Beijing Artificial Intelligence Institute, Faculty of Information Technology, Beijing University of Technology, Beijing, China; 10 School of Aerospace, Mechanical and Mechatronic Engineering, The University of Sydney, Sydney, New South Wales Australia

**Keywords:** Metals and alloys, Structural properties

## Abstract

Many nanosized metals with limited dislocation nucleation and multiplication usually exhibit poor plasticity. Previous studies have demonstrated that multi-principal element alloys (MPEAs) can achieve a combination of strength and plasticity. However, whether these mechanical properties extend to their nanosized counterparts and how nanosized MPEAs accommodate plastic deformation remains unknown. Using a homemade in-situ device, we captured the atomic-scale tensile process and measured the stress-strain curve of Fe_48_Mn_32_Co_10_Cr_10_ nanosized single crystals (NSCs). We find that NSCs achieve a strength of ~3 GPa and a uniform elongation of ~20%. We show that this combination arises from the inhomogeneous distribution of enthalpy, which produces a broad spectrum of energy barriers for the nucleation of full dislocations, partial dislocations, and nanotwins. Consequently, dislocations and nanotwins are randomly generated throughout MPEA NSCs, effectively suppressing local stress concentration and leading to both high strength and large uniform elongation.

## Abstract (web summary)

Nanosized multi-principal element alloy single-crystals achieve both strength ~3 GPa and uniform elongation ~20% due to uneven enthalpy distribution, which promotes random, widespread defect formation and prevents localized stress buildup.

## Introduction

Previous experiments and simulations have shown that many nanosized metals exhibit high strengths but usually suffer from low plasticity. During deformation, high stress is required for the nucleation of new dislocations, and dislocations can easily escape rather than multiply, leading to many nanosized metals exhibiting high strength but low plasticity^1–3^. This strength-plasticity trade-off phenomenon has been supported by a large number of studies on pure metals. In recent years, multi-principal element alloys (MPEAs) exhibit exceptional mechanical properties^4–6^, which seem to overcome this strength-plasticity trade-off phenomenon. For example, studies on the Cantor alloy demonstrated excellent crack resistance^7,8^. Previous studies have also shown that FeMnCoCr-based MPEAs exhibit remarkable strength, plasticity, and a high strain hardening rate^9,10^. These studies suggest that MPEAs frequently exhibit multiple deformation mechanisms, including full dislocation slip, deformation twinning, and phase transformations between face-centered cubic (FCC) and hexagonal close-packed (HCP) phases^11,12^, which contribute to the superior mechanical properties of MPEAs^13–15^. However, most studies to date have been conducted on bulk or relatively large samples. Whether these exceptional mechanical properties can still be preserved when the size of MPEAs decreases into the nanosized, and how the nanosized MPEAs accommodate plasticity, remains an open question. Furthermore, previous studies on microsized single crystal metals such as Au, Cu, Ni, and Al have revealed a dislocation “starvation” phenomenon^16–18^. As the size of these pure metals decreases to tens of nanometers, their deformation modes shift from full dislocation activity to partial dislocations or twinning^19,20^, as further confirmed by molecular dynamics (MD) simulations^21,22^. Yet, in nanosized MPEAs, the adjacent atomic bonds exhibit considerable variation across different regions, raising the question of whether the deformation mechanisms observed in pure metals are directly applicable. Several notable studies suggest that dislocation behavior in MPEAs is significantly influenced by short-range chemical ordering^23–25^, non-uniform stacking fault energy (SFE) distribution^26–28^, and negative enthalpy states^29,30^. These findings emphasize that local chemical variations can significantly affect the deformation mechanisms of MPEAs. However, most investigations are based on ex-situ nanoscale observations, lacking direct in-situ atomic-scale evidence. Therefore, it is highly desirable to achieve both high strength and large plasticity in nanosized MPEAs, as well as to reveal their atomic-scale mechanisms.

In this work, we employed a self-developed in-situ device capable of observing the atomic-scale deformation process while simultaneously measuring the strain-stress curve of nanomaterials. We dynamically observed at the atomic scale the plastic deformation behavior of Fe_48_Mn_32_Co_10_Cr_10_ nanosized single crystals (NSCs) in a transmission electron microscope (TEM). The NSCs exhibited the strength of ~3 GPa combined with a uniform elongation of ~20%, demonstrating a strength-plasticity synergy. In-situ atomic-scale observations revealed that deformation is accommodated through the random emission of full dislocations, partial dislocations, and nanotwins, which imparts both ultrahigh strength and large plasticity on the nanosized MPEAs. These distinctive deformation modes originate from the tendency for elements with negative mixing enthalpy to cluster, resulting in variations in local mechanical properties and a broad spectrum of energy barriers for the dislocations and nanotwins nucleation. Consequently, random stacking faults (SFs), full dislocations, and nanotwins are equally favorable in MPEA NSCs. These results demonstrate that the mixing enthalpy significantly influences the mechanical behavior of MPEAs, which have been overlooked previously.

## Results

### Atomic-scale element distribution

**Fig. 1:**
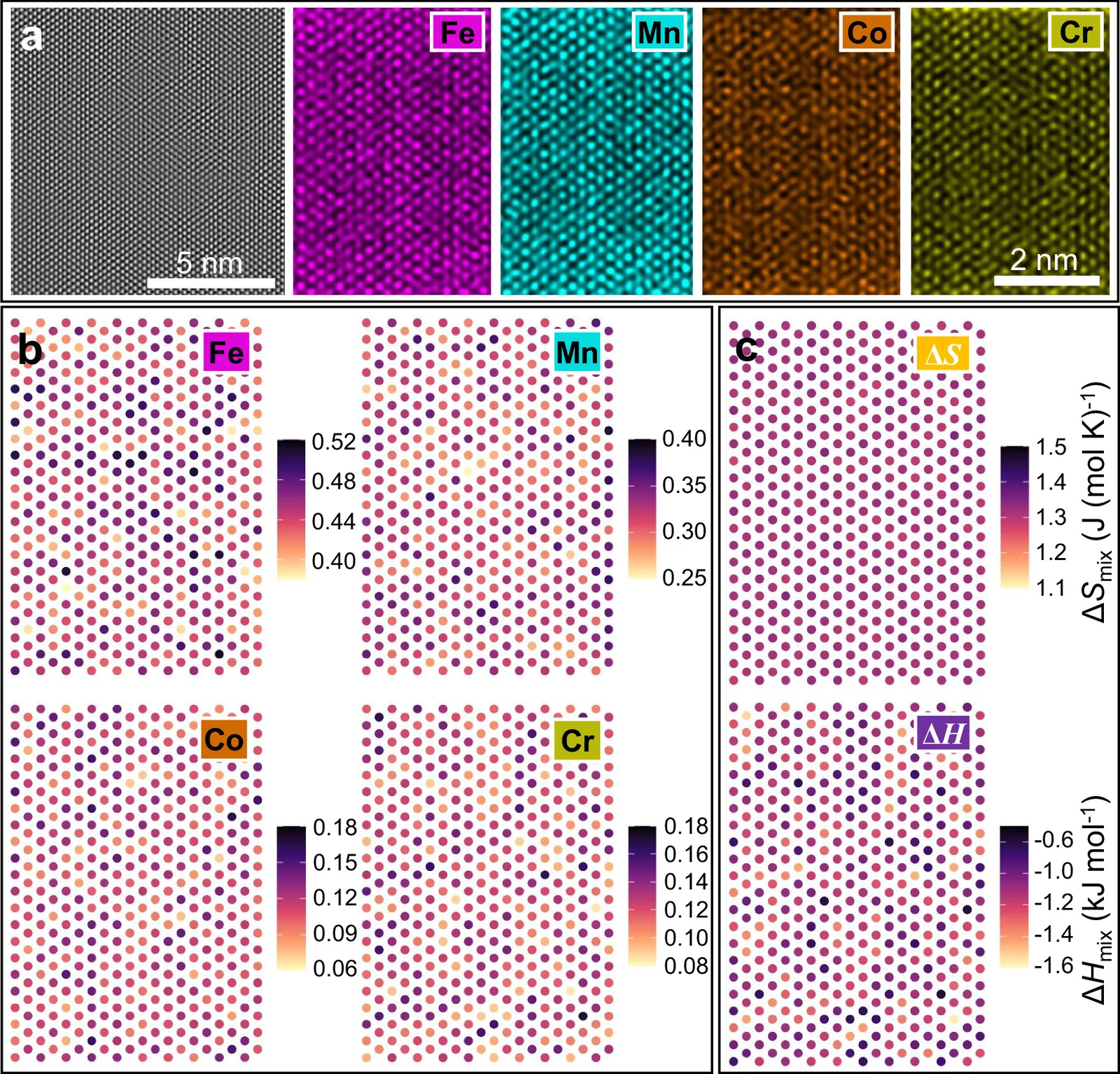
Atomic-scale element distribution. **a** High-angle annular dark-field (HAADF) image and corresponding atomic-scale energy-dispersive X-ray spectroscopy (EDS) maps of Fe, Mn, Co, and Cr elements in the nanosized single crystals (NSC). **b** Quantified two-dimensional elemental maps of Fe, Mn, Co, and Cr, demonstrating inhomogeneous elemental distributions at the atomic scale. **c** Atomic-level distributions of configurational entropy Δ*S* and mixing enthalpy Δ*H*.

Figure 1a presents an atomic-scale high-angle annular dark-field (HAADF) image, along with the corresponding energy-dispersive X-ray spectroscopy (EDS) maps for Fe, Mn, Co, and Cr in the NSC. Using our recently developed method, the quantified elemental maps in Fig. 1b reveal an inhomogeneous element distribution. For instance, the Fe content varies from approximately 52% to 38%, as indicated by a color gradient spanning from deep blue to deep red. Based on atomic-scale EDS maps, the atomic-level distributions of configurational entropy and mixing enthalpy were calculated, as shown in Fig. 1c. Because Fe, Mn, Co, and Cr are adjacent first-row transition metals with very similar metallic radii (Fe: 127 pm, Mn: 132 pm, Co: 126 pm, Cr: 127 pm), the effect of elastic strain (due to atomic size mismatch) on the enthalpy calculation can be neglected^31,32^. The configurational entropy (Δ*S*) is relatively homogeneous, with slight variations from 9.78 to 10.72 J (mol K)^−1^. In contrast, the mixing enthalpy (Δ*H*) exhibits negative values, ranging from −1.53 to −0.78 kJ mol^−1^, and is distributed inhomogeneously within the NSC. This negative mixing enthalpy arises from deviations in chemical affinity, leading to the formation of high-strength atomic bonds^30,33–35^. On the one hand, elements with negative mixing enthalpy prefer to gather together^29^, resulting in regions with strong atomic bonds and enhanced strength. On the other hand, this inhomogeneous distribution of enthalpy generates locally varying chemical environments, leading to non-uniform energy barriers for dislocation nucleation and motion^27,36^. Consequently, the inhomogeneous distribution of negative mixing enthalpy contributes to more complex dislocation behavior in MPEAs.

### In-situ tensile process

**Fig. 2:**
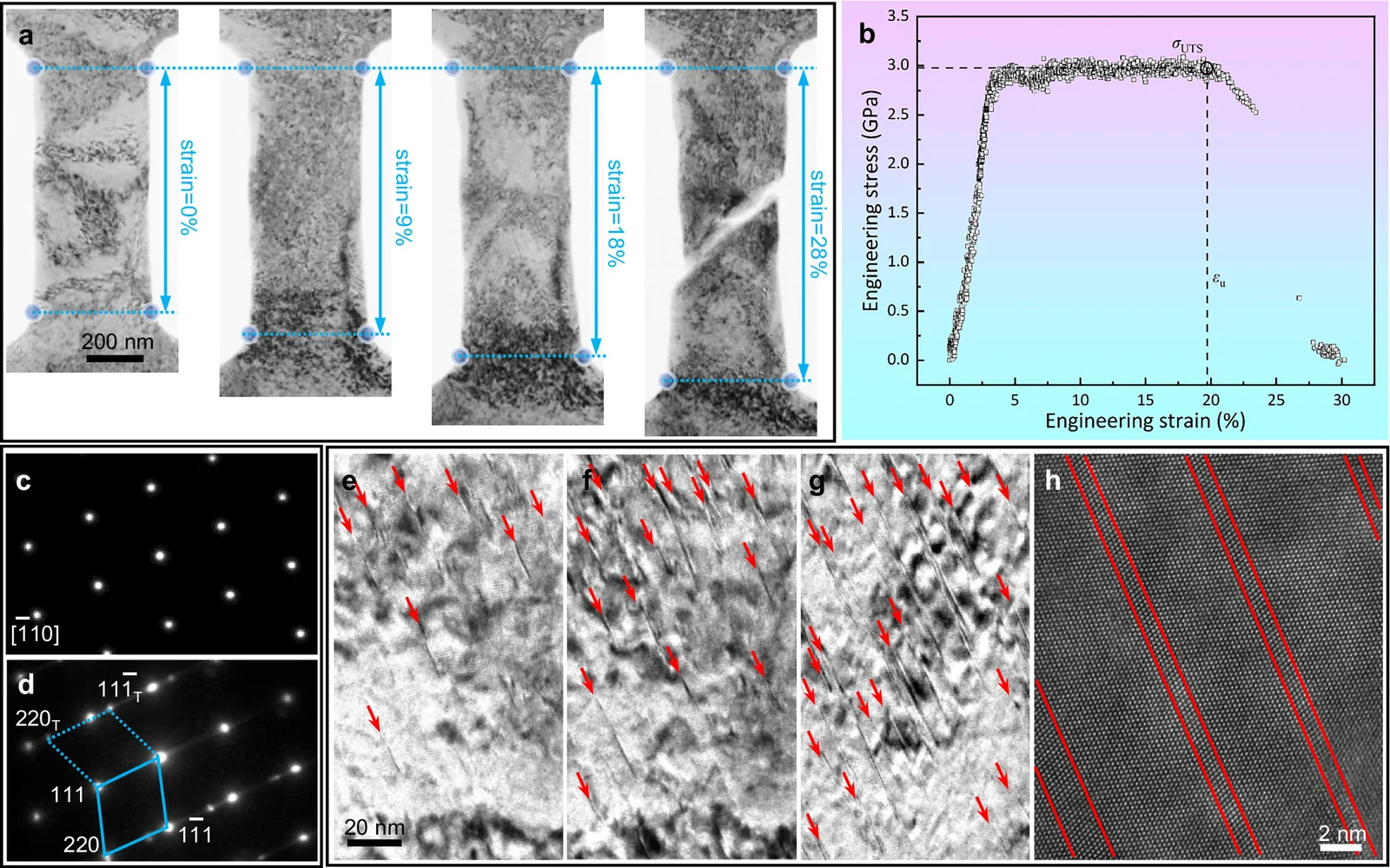
In-situ observation of the tensile process. **a** Bright-field TEM images captured during in-situ tensile straining at low magnification. **b** Tensile stress-strain curve of the NSC, *σ*_UTS_ is the ultimate tensile strength, *ε*_u_ is the ultimate elongation. **c**, **d** SAED patterns before and after deformation, illustrating the formation of SFs and nanotwins. **e**–**g** Magnified TEM bright-field images of the deformation in the early stage, showing the propagation of SFs (red arrows) and nanotwins. **h** HAADF image of the deformed specimen, with solid lines marked coherent twin boundary (CTB). Source data are provided as a Source Data file.

Figure 2a displays a series of bright-field TEM images captured along a < 110> zone axis during the tensile deformation of the NSC. The initial dimensions of the NSC were ~ 800 nm in length, ~ 400 nm in width, and ~ 60 nm in thickness. Uniaxial tensile loading was applied along the <111> direction perpendicular to the TEM beam direction, resulting in three distinct stages: (a) uniform plastic deformation, (b) stress concentration and rapid local shear, and (c) crack initiation, propagation, and fracture. We conducted uniaxial tensile experiments using a self-developed in-situ device on the NSC, and the corresponding strain-stress curve was measured simultaneously (Supplementary Figs. 1–3). As shown in Fig. 2b, the engineering tensile stress-strain curves revealed that the NSC exhibited a uniform tensile strain of ~ 20%, and a strength reaching ~ 3.0 GPa. Based on the engineering/true strain-stress curve, strain-hardening rate curve (Supplementary Fig. 4) and in situ TEM images (Supplementary Fig. 5), after the true strain exceeds ~ 17%, the NSC fractures rapidly through local shear, followed by crack initiation and propagation. At the early stage (true strain below 5%), the NSC exhibits strong strain hardening (Supplementary Fig. 4d). As the true strain increases to the range of 5-17%, the normalized strain-hardening rate remains at ~1.5 (150%). This relatively stable normalized strain-hardening rate ensures the relatively large uniform elongation of the NSC. After the true strain exceeds ~17%, the normalized strain-hardening rate falls below 1.0 (100%) and drops sharply. At this point, the readily mobile dislocations have been exhausted, and further deformation becomes increasingly localized in high-stress regions, where dislocation activities with much higher energy barriers are required. Consequently, the NSC can no longer disperse stress through uniform plastic deformation and instead undergoes rapid local shear followed by crack initiation and propagation (Supplementary Fig. 5). This may explain why the true stress-strain response of the NSC differs from that typically observed in bulk materials. Figure 2c displays the selected area electron diffraction (SAED) pattern before plastic deformation, revealing a typical FCC structure. After deformation, as shown in Fig. 2d, additional spots appear in the SAED pattern, indicating twin structures characteristic of FCC metals^37^. Figure 2e–g shows magnified TEM images illustrating the early-stage deformation. Nanotwins are indicated by red arrows. During the in-situ process, the number of fine nanotwins progressively increases with applied strain, without significant twin thickening (Supplementary Movie 1). Figure 2h presents the HAADF image of the deformed specimen, confirming the widespread presence of nanotwins. Notably, these twins grew to only a few nanometers in thickness and ceased extending under further loading. This behavior contrasts with that observed in nanosized pure metals, where twins can readily extend to several tens of nanometers in thickness. We also conducted tests on NSCs with thicknesses of 160 nm and 230 nm (Supplementary Figs. 6 and 7). The in-situ observations show that there are also abundant SFs and nanotwins, indicating the observed deformation mode should not be an artifact of FIB damage. The true strain-stress curve shows a trend that, as the thickness of NSCs increases, their strength decreases while elongation increases. This size effect is consistent with previous studies^38^.

**Fig. 3:**
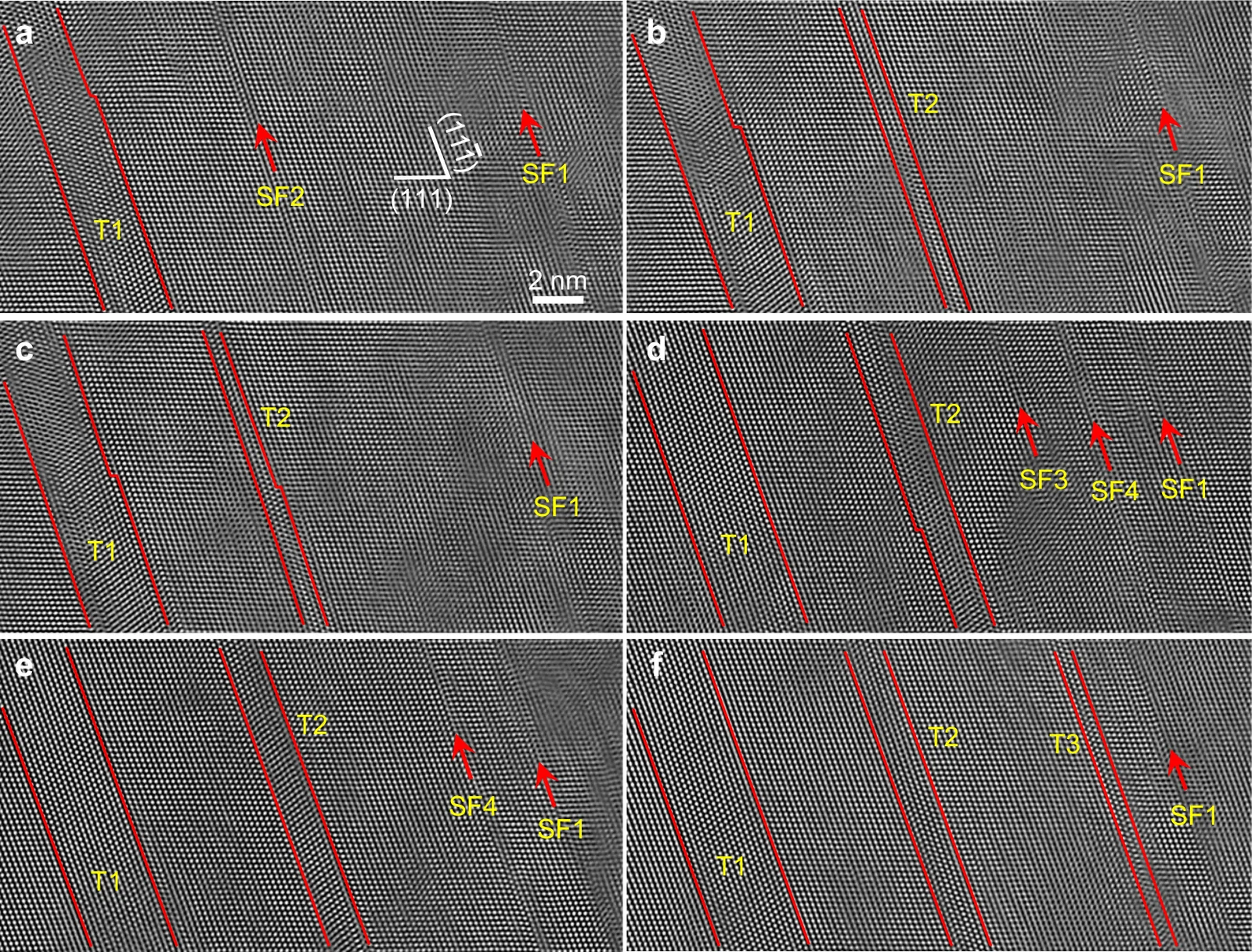
Atomic-scale observation of the NSC during in-situ tensile straining. **a**–**f** Red lines mark the twin boundaries (TBs), and arrows mark stacking faults (SFs). The images illustrate how the continuous emission of partial dislocations leads to the transformation of SFs into deformation twins and their subsequent growth. The sequence also depicts the nucleation of SFs from free surfaces, resulting from the random emission of partial dislocations.

Figure 3 presents in-situ atomic-scale evidence that the plastic deformation of the NSC is mediated by the random generation of deformation twins and partial dislocations from the onset of plastic deformation up to ~5% strain. Initially, most partial dislocations glide parallel to twin boundaries (TBs). In Fig. 3a, a deformation twin (T1) and two stacking faults (SF1 and SF2) emerge in this region. The TB is marked by red lines, while red arrows indicate SFs. A step on the right TB of T1 indicates the presence of a partial dislocation. Throughout the in-situ observation, the position of SF1 remains stationary, serving as a reference point. As loading continues, as shown in Fig. 3b, a new twin (T2) forms at the location of SF2, and the thickness of T1 increases through migration of the step on the right TB of T1. With further tensile loading, as seen in Fig. 3c, a step forms and glides on the right TB of T2, leading to TB migration and an increase in the thickness of T2. Figure 3d shows the formation of two new stacking faults, SF3 and SF4, as T2 continues to grow via partial dislocation generation and glide along the TB under continued loading. As loading progresses further, as shown in Fig. 3e, f, SF4 transforms into a new twin (T3), while the pre-existing SF3 disappears due to the generation of a trailing partial dislocation on the same glide plane as SF3. During this process, the thicknesses of deformation twins T1 and T2 remain unchanged. These atomic-scale observations reveal intriguing behavior: twins cease to grow after reaching a certain thickness, and partial dislocations are randomly generated, resulting in the random distribution of SFs and twins within the NSC. This random generation of dislocations helps suppress stress concentration, thereby enhancing the plasticity of the NSC^39–41^.

**Fig. 4:**
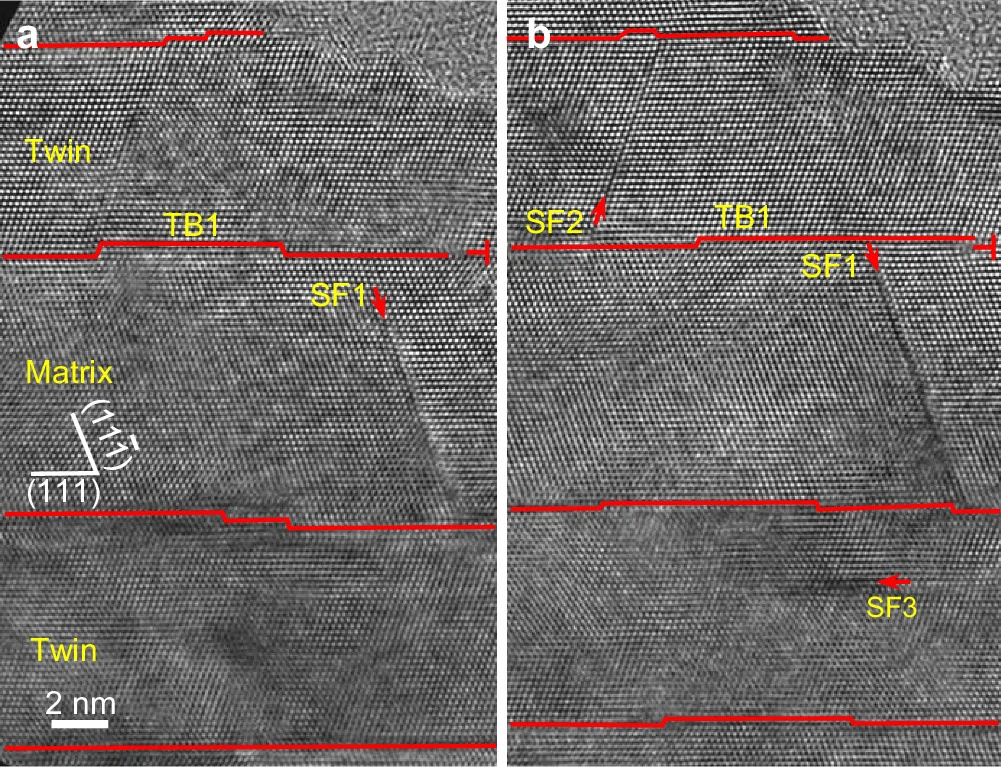
HRTEM images captured during in-situ tensile straining of the NSC. Red lines mark the TBs. Two deformation twins are shown within the FCC matrix, with steps present on the TBs. **a** SFs are present in both the twins and the matrix, marked by red arrows. **b** Under stress, the steps migrate, and SFs move, demonstrating the emission of partial dislocations on different {111} planes.

Following the formation of deformation twins, both full and partial dislocations intersecting with the TBs emerge as critical deformation modes. Figure 4 presents the HRTEM images taken from the ~5% to ~15% strain stage, showing full and partial dislocations on two intersecting {111} planes. In Fig. 4a, a full dislocation (marked “T”) is observed on the intersection plane of the TB and is pinned at TB1. Additionally, an SF resulting from a partial dislocation (denoted as SF1) is generated on an intersecting plane of the TB. This SF is approximately 2 nm away from TB1 and moves toward the TB. With continued loading, as shown in Fig. 4b, this partial dislocation moves upward and intersects with TB1 (SF1 is seen intersecting with the TB). Simultaneously, two new stacking faults (SF2 and SF3) appear in this region. SF2 intersects with TB1 due to the glide of a partial dislocation along the intersection plane of the TB, while SF3 is parallel to the TB, caused by a partial dislocation gliding on a plane parallel to the TB. These atomic-scale observations reveal the processes of full and partial dislocations intersecting with TBs during deformation.

**Fig. 5:**
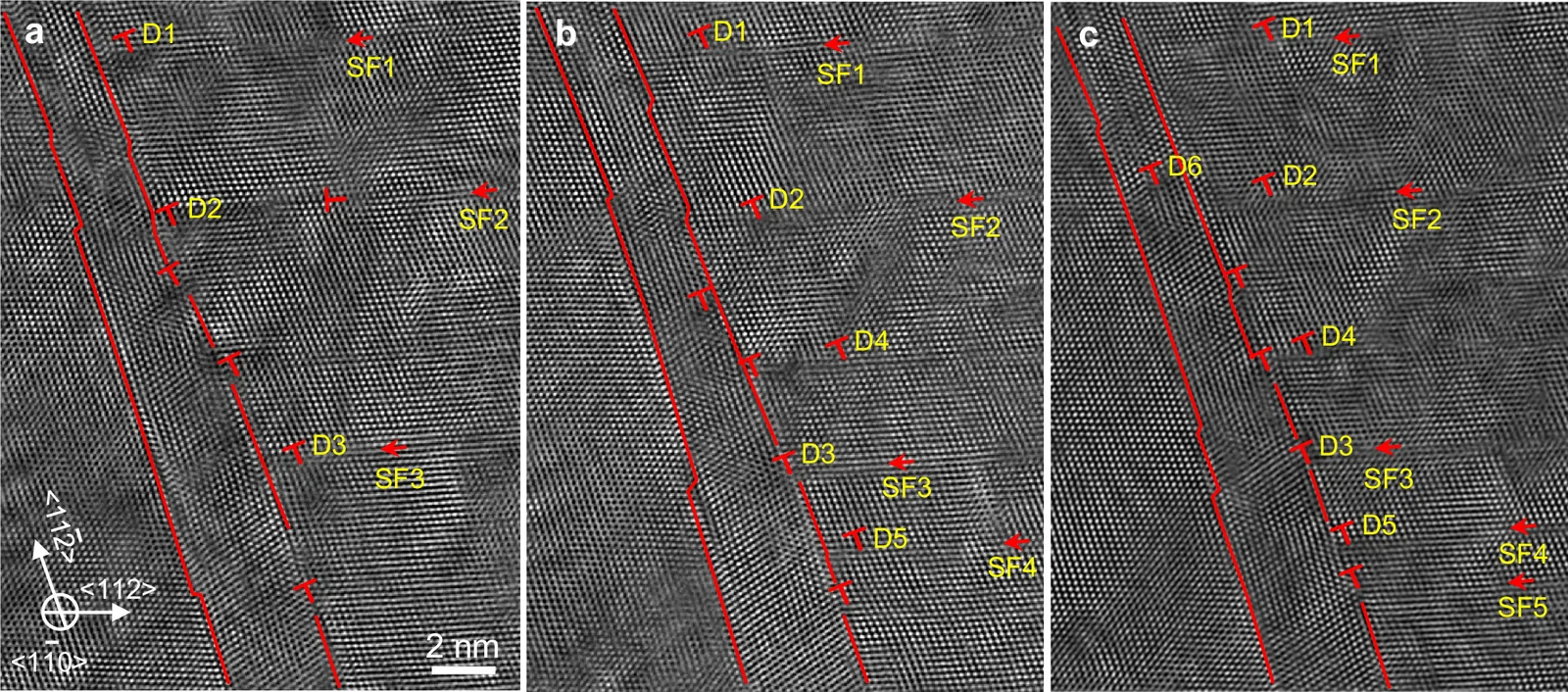
HRTEM images illustrating the interaction between partial dislocations and TBs during tensile straining of the NSC. **a**–**c** A twin is present on the left side, with the TBs marked by red lines. On the right side of the twin, numerous full dislocations are visible, denoted by “T”, along with several SFs, indicated by red arrows. As loading progresses, randomly emitted partial dislocations accumulate at the TB, forming full dislocations and contributing to strain hardening.

Figure 5 presents a typical example of in situ HRTEM images that were captured at a strain of ~15%, where both dislocation-TB interaction and partial-full dislocation interaction were observed. As shown in Fig. 5a, many full dislocations on {111} planes are blocked by the TB (marked with “T”). By comparing the positions of full dislocations D1 in Fig. 5a, b, it seems that this dislocation nucleates from the free surface intersection, and moves from the right side toward the coherent twin boundary (CTB) and is blocked by the CTB. As shown in Fig. 5b, c, full dislocations D4 and D5 also nucleate from the free surface; they move from the right side toward the CTB and are then blocked by the CTB. While comparing the positions of full dislocations D2 and D3 in Fig. 5b, c, it can be seen that these two dislocations are located near the CTB, and move from the CTB toward the right side, demonstrating that these full dislocations nucleate from the CTB-surface intersection. Besides full dislocation, many partial dislocations nucleate and intersect with CTBs, leaving SFs that terminate at the CTB, as shown in Fig. 5a, b (labeled “SF1-SF3”). The arrows indicate that these SFs move from the right side toward the CTB, increasing and becoming progressively closer to the CTB, demonstrating that these partial dislocations nucleate from the free surface. As shown in Fig. 5b, c, the newly formed SF4 and SF5 underwent a similar process as SF1-SF3. In addition, both the steps on the CTB and the width of the twin have changed (Fig. 5a–c), which results from partial dislocations nucleating from the CTB-surface intersection and gliding along the CTB, which is similar to what is shown in Fig. 3. This further confirms that the plastic deformation of the NSC is governed by the random emission of full and partial dislocations across multiple slip systems. The current study reveals that the deformation mechanism of NSC differs fundamentally from that in nanosized pure metals, where plastic deformation is typically governed by partial dislocations and twinning, allowing deformation twins to grow to several tens of nanometers^42,43^.

Generally, the above observed deformation mode increases the probability of interactions between full and partial dislocations and TBs. Based on statistical analysis of the HRTEM images, the dislocation density in the late stage of deformation is approximately 5.79–7.23 × 10^16^ m^−2^. These dislocation-TB interactions not only accommodate plastic deformation but also contribute to strain hardening^44^. In addition, chemical inhomogeneity—specifically, mixing of elements with different atomic radii resulting in lattice distortion—and solid solution strengthening effects also contribute to strain hardening^45^. Besides strain hardening, several factors lead to softening. First, large free surfaces allow numerous nucleated dislocations to easily escape. Second, many partial dislocations nucleate and glide parallel to the TBs, leading to TB migration. The synergy of these mechanisms results in a balance between strain hardening and softening, as observed in the flat plateau of the strain-hardening rate curve (Supplementary Fig. 4). In the later stage of deformation, those regions that can easily nucleate dislocations become exhausted, and strain is then accommodated by dislocation activities with a higher energy barrier that occurs in those high-stress regions. As a result, local shear readily occurs, and the balance between strain hardening and softening is disturbed. Consequently, the strain-hardening rate decreases to values below 1.0, and the NSC can no longer effectively disperse stress through uniform plastic deformation; instead, it becomes prone to rapid local shear and is followed by crack initiation and propagation (Supplementary Fig. 5).

### Computational simulation

**Fig. 6:**
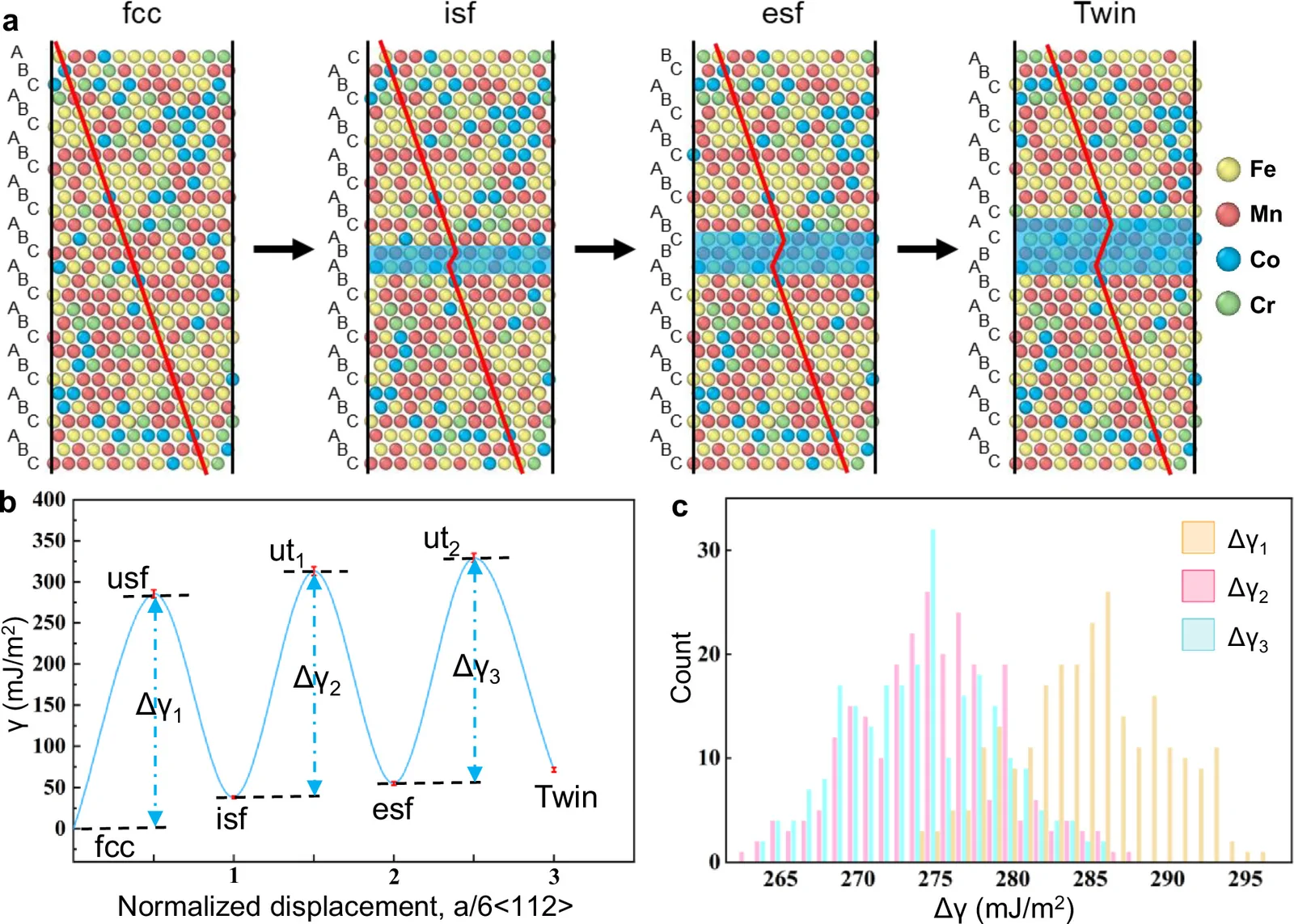
The simulation of GSFE in NSC. **a** Schematic of the twin formation pathway. The formations of a one-layer intrinsic stacking fault (ISF), two-layer extrinsic stacking fault (ESF), and three-layer twins are created by introducing Shockley partials with b = 1/6 <112> on the adjacent {111} plane. **b** Generalized stacking fault energy (GSFE) curve of the NSC along the twin formation pathway, usf: unstable stacking fault energy, ut_1_: unstable twinning energy for the first partial slip on the SF plane and ut2: unstable twinning energy for the second partial slip on the twin plane. **c** Distribution of unstable SFE barriers for three selected paths: FCC to ISF, ISF to ESF, and ESF to twin, corresponding to Δ*γ*_1_, Δ*γ*_2_, and Δ*γ*_3_ in (**b***)*, respectively. Source data are provided as a Source Data file.

To elucidate how the inhomogeneous distribution of negative mixing enthalpy indeed can affect the emission of partial dislocations and the formation of deformation twins in the NSC, the nudged elastic band (NEB) method^46^ was employed to calculate the generalized stacking fault energy (GSFE) of the alloy (see “Methods”). As illustrated in Fig. 6a, the schematic outlines the pathway of deformation twin formation through the glide of 1/6 <112> Shockley partial dislocations on adjacent {111} planes. The first Shockley partial creates an intrinsic stacking fault (ISF), followed by the second and third Shockley partials on adjacent {111} planes, leading to the formation of an extrinsic stacking fault (ESF) and a three-layer twin, respectively. In Fig. 6b, Δ*γ*_1_, Δ*γ*_2_, and Δ*γ*_3_ represent the energy barriers for the formation of ISF, ESF, and twin in the NSC, respectively. The values of Δ*γ*_2_ and Δ*γ*_3_ are slightly smaller than Δ*γ*_1_, and their distributions exhibit significant overlap. This is in stark contrast to the typical relationship observed in pure metals and conventional alloys (Δ*γ*_1_ ≫ Δ*γ*_2_ = Δ*γ*_3_)^39,40^. In pure metals, partial dislocations tend to emit continuously, which favors the formation of thick twins over the generation of new SFs. This is because the energy barrier for nucleating subsequent partial dislocations on crystallographic planes adjacent to the first partial (resulting in twin formation) is substantially lower than that for nucleation on distant planes (which leads to SFs). However, in the NSC, the inhomogeneously distributed negative mixing enthalpy leads to locally high-strength mechanical properties and varying local energy barriers for the formation of SFs and twins. This results in widely distributed and overlapping energy barriers for separated partial dislocations, full dislocations, and deformation twins. Consequently, this enables the generation of separated full dislocations, SFs, and deformation twins. Once a plane with a high energy barrier is encountered, twin thickening ceases, and the strain is accommodated by an SF nucleating elsewhere at a site with a lower Δ*γ*_1_. As a result, a high density of separated SFs and sub-nano-scale deformation twins is formed (Supplementary Figs. 8 and 9), rather than individual thick twins^27^.

In addition, we calculated the GSFE curves under tensile strains of 1% and 2% using MD simulations (Supplementary Fig. 10). The results reveal a pronounced stress/strain dependence in the NSC MPEA. All GSFE parameters systematically decrease with increasing strain, and their distributions also exhibit significant overlap, which is consistent with previous calculations^47^. While it should be noted that, because the actual potential of MPEAs is difficult to determine, the calculated values of GSFE may vary when using different potentials. Thus, the MD simulation may only represent a trend. In many cases, even the qualitative trends may depend strongly on the choice of interatomic potential.

## Discussion

Over the past decades, extensive research has been conducted on the dislocation behaviors of nanosized pure metals, with numerous studies demonstrating that their plastic deformation is primarily mediated by partial dislocations and twins^46,48–52^. In pure metals, low and uniform energy barriers facilitate the thickening of existing SFs or twins during plastic deformation^26,53^. For instance, the plasticity of Au, Cu, Ag, and Ni nanowires is typically dominated by thick twins spanning the gauge section^54–59^. In contrast, the thicknesses of deformation twins in NSC are generally only a few nanometers, with full dislocations, deformation twins and separated SFs randomly distributed. Our study presents the in-situ atomic-scale experimental results elucidating the deformation mechanisms in nanosized MPEAs. In MPEAs, those elements with negative enthalpies tend to aggregate together, leading to a non-uniform distribution of chemical elements^30,33,34,60^ and significant variations in energy barriers. When locally higher energy barriers are encountered, the twin thickening process halts, and new dislocations are generated randomly at another site, therefore leading to the random distribution of full dislocations, partial dislocations, and deformation twins. This deformation mode leads to the nanosized MPEA exhibiting both ultrahigh strength and large uniform elongation.

Actually, nanosized metals do not always exhibit poor ductility. Based on previous classic experiments and theoretical studies, in most cases, nanosized metals readily exist in a low dislocation density state, require high stress for the nucleation of new dislocations, and exhibit limited dislocation multiplication during deformation. These features make nanosized metals prone to localized deformation and early fracture, thereby leading them to usually exhibit ultrahigh strength but poor plasticity^61–63^. However, there are many cases where nanoscale metals can also exhibit large strength and high ductility. Previous studies show that the random nucleation of dislocations leads to large uniform elongation in nanoscale Cu^64^. Large uniform elongation that resulted from random generation of martensitic transformation was also observed in nanoscale polycrystalline NiTi^65^. These deformation modes are similar to those observed in the current study, which can effectively alleviate deformation localization, thereby enabling nanoscale metals to exhibit both ultrahigh strength and large uniform elongation. Our current results, combined with previous classical studies, suggest that equipping nanoscale metals with the ability to randomly nucleate new dislocations or deformation twins/phases is an efficient method to overcome the strength-ductility trade-off in nanoscale metals.

From a thermodynamic perspective, it should be emphasized that we do not deny the role of entropy in strengthening MPEAs, but rather that enthalpy is also crucial for improving their mechanical properties. According to the Gibbs free energy formula Δ*G* = Δ*H* - *T*Δ*S*, decreasing enthalpy or increasing mixing entropy can lower the system’s Gibbs free energy, making MPEAs thermodynamically stable. We performed GSFE MD simulations on binary and ternary alloys with varying mixed entropy and enthalpy, as shown in Supplementary Fig. 11 ~16. On the one hand, reducing Δ*H* promotes element segregation/inhomogeneity and the formation of phases or intermetallic compounds. In MPEAs, regulating enthalpy to a suitable value is necessary to achieve both high strength and large uniform elongation (as shown in this study). Excessively negative enthalpy promotes the formation of intermetallic compounds, leading to high strength but poor plasticity. Conversely, high (positive) enthalpy provides only a limited strengthening effect. On the other hand, increasing entropy drives homogeneous distribution of alloy atoms and suppresses element clustering and the formation of independent phases or intermetallic compounds. High entropy drives uniform mixing of elements with different atomic radii, resulting in lattice distortion and solid solution strengthening, thereby also improving the hardness and strength of MPEAs^45^. Therefore, the excellent properties of MPEAs originate from the synergistic effect of enthalpy and entropy, rather than a single factor.

Here, it should be noted that there are many factors that can affect the mechanical behavior of metals. First, the strain rate can significantly affect the uniform elongation and deformation mechanism of metals. In current experiments, the strain rate is in the range of 10^−4^–10^−3^ s^−1^. Such conventional strain rates should have a negligible effect on deformation twins, as only high strain rates (e.g., >10^2^ s^−1^) can promote twinning/SF activity^66,67^. Thus, the deformation twins and SFs observed in our experiments are intrinsic deformation mechanisms in the NSC rather than effects of strain rate.

Second, grain size also influences deformation mechanisms. Based on previous studies, as grain size decreases to a critical value (e.g., ~15 nm), the deformation of nanosized polycrystalline metals transitions from intragranular activities (dislocation slip and twinning) to intergranular mechanisms (grain boundary (GB) sliding, migration, and grain rotation)^68^. Thus, it is reasonable to infer that nanosized polycrystalline MPEAs should exhibit similar trends. In addition, both intragranular and intergranular deformation mechanisms can be significantly affected by GBs and chemical inhomogeneity; the dislocation activities, twinning mechanisms, and GB plasticity in nanosized polycrystalline MPEAs should differ substantially from pure metals and NSC MPEAs^38,69^. Third, the loading direction can affect the uniform elongation of metals. We also conducted tensile tests of NSC MPEAs with orientations of <110> and <001> (Supplementary Figs. 17 and 18), the results indicate that loading orientation can influence macroscopic mechanical response, such as uniform elongation. However, the underlying deformation mechanisms—governed by the random activation of full dislocations, partial dislocations, and nanotwins—remain consistent across different orientations. This suggests that while orientation affects certain mechanical properties, it does not change the intrinsic deformation behavior of nanoscale MPEAs, which is primarily determined by their chemical inhomogeneity.

In summary, we have developed an in-situ device capable of observing the atomic-scale deformation process while simultaneously measuring the strain-stress curve of NSCs. Such a device overcomes the long-term challenge of simultaneously observing these two key datasets. We revealed that the inhomogeneously distributed negative mixing enthalpy enables the NSC to exhibit both high strength and large uniform elongation. The inhomogeneously distributed negative mixing enthalpy makes a wide range of energy barriers for the formation of dislocations and deformation twins in MPEA. This results in the random distribution of full dislocations, SFs, and deformation twins within the NSC, which can effectively suppress stress concentrations, thereby enhancing plasticity. Furthermore, the interaction of these dislocations with TBs contributes to high strength. Our findings provide an atomic-scale insight into the deformation mechanisms of nanosized MPEAs and offer valuable guidance for future investigations into the deformation mechanisms of metallic alloys.

## Methods

### Material and in-situ tensile process

An experimental alloy ingot with a nominal composition of Fe_48_Mn_32_Co_10_Cr_10_ MPEA was synthesized using arc melting of pure metals, each having a purity greater than 99.9 wt.%. To ensure chemical homogeneity, the ingot underwent five remelting cycles in an argon atmosphere. Following this, the ingot was hot rolled at 1373 K. Subsequent homogenization was conducted at 1473 K for 4 h. Cold rolling of the alloy was then carried out at room temperature. The cold rolling process achieved a 75% depression rate, resulting in a final sheet thickness of 1.2 mm. The cold-rolled specimens underwent annealing at 1023 K for 1 h. The MPEA NSC was derived from an annealed polycrystalline bulk alloy. The NSC tensile sample was extracted from grains aligned with the [110] zone axes from the bulk alloy. This extraction was performed using a FEI Helios G4 UC focused ion beam (FIB). The NSC tensile sample was attached to an in-situ tensile device we developed (Supplementary Figs. 1–3) with double-tilt and stress-strain measurement capability and observed using an FEI Titan environmental transmission electron microscope.

The Fe₄₈Mn₃₂Co₁₀Cr₁₀ NSC was selected as the model system. This is a well-known system with exceptional mechanical properties that has attracted intense interest ^9^. In addition, it is suitable for investigating how negative‑enthalpy‑induced local chemical fluctuations influence the mechanical behavior of multi‑principal element alloys. This system exhibits negative enthalpy and covers a wide range of SFE values. Moreover, the metallic radii of Fe, Mn, Co, and Cr are very similar, allowing the elastic strain energy contribution to enthalpy to be neglected. Such a design enables the isolated examination of enthalpy effects on the deformation behavior of nanosized single‑crystal MPEAs. Furthermore, we also notice that most previous studies have focused on bulk polycrystalline MPEAs (containing numerous GBs) or relatively large samples; research on the mechanical behavior of NSC MPEAs remains scarce. Finally, nanoscale single crystals without GBs eliminate the complicating effects of GBs, which helps to more accurately investigate the effect of negative mixing enthalpy on the mechanical behavior of MPEAs. To verify the reproducibility of the observed deformation mechanisms, independent in situ TEM tensile experiments were conducted on three specimens prepared under identical conditions. Consistent deformation behaviors were observed across these independent experiments. The images presented in the main text are representative of these observations.

### Calculation of quantified element maps

The MPEA was characterized using the FEI Themis Z transmission electron microscope for HAADF image and atomic-level EDS maps. With the atomic-scale EDS data and corresponding HAADF atomic column coordinates, the center positions of each atomic column were computed using the open-source software Atomap. Subsequently, the positions of atomic columns were mapped onto the EDS data, and the average intensity of image pixels within small neighborhoods around the atomic coordinates was calculated. This intensity value represents the relative intensity of the element in the atom column, and the relative intensities of Fe, Mn, Co, and Cr were *A*_*i*_, *B*_*i*_, *C*_*i*_, and *D*_*i*_, respectively. The relative content was calculated based on the relative intensities of Fe, Mn, Co, and Cr. The formula is as follows:

1 $${P}_{i}=\frac{{A}_{i}}{{A}_{i}+{B}_{i}+{C}_{i}+{D}_{i}}$$

where *P*_*i*_ represents the relative percentage content of Fe in the atom column. Based on the *P*_*i*_ and the atom column coordinates, we drew two-dimensional images of the Fe (Mn, Co, and Cr) percentage content, as shown in Fig. 1b. The different colors represent different content.

### The calculations of the mixing enthalpy and configurational entropy

The mixing enthalpy of MPEA can be calculated by^29^:

2 $$\Delta {H}_{{\mbox{mix}}}=4{\sum }_{i < j}{c}_{i}{c}_{j}\Delta {H}_{{ij}}$$

where *c*_*i*_ and *c*_*j*_ are the mole fractions of element *i* and *j* calculated by atomic-scale EDS and quantified elemental maps. *∆H*_*ij*_ is the mixing enthalpy of element *i* and *j* in their binary liquid system (Supplementary Fig. 19).

The mixing entropy can be calculated by:

3 $$\Delta {S}_{{\mbox{mix}}}=-R{\sum }_{i=1}^{n}{c}_{i}{{\mathrm{ln}}}{c}_{i}$$

where *R* is the universal gas constant.

### Simulation of generalized stacking fault energy

The GSFE curves for multiple alloy compositions were calculated via a large-scale atomic/molecular massively parallel simulator (LAMMPS) to assess their intrinsic deformation propensity. The atomic interactions in the quaternary Fe_48_Mn_32_Co₁₀Cr₁₀ alloy were modeled using the potential developed by Gröger^70^. A total of 250 independent simulation cells were constructed for each composition to ensure statistical significance, as compositional fluctuations in these multi-component systems can significantly influence the GSFE^46^. Each cell contained a planar fault area of approximately 4.2 × 5.4 nm within the FCC lattice. To determine the GSFE pathways, three characteristic planar faults were created from the perfect FCC structure: a one-layer ISF, a two-layer ESF, and a three-layer twin. These defects were generated by rigidly shifting the stacking close-packed (111) atomic planes in sequence. Energy pathways (i.e., from perfect FCC to ISF, ISF to ESF, and ESF to twin) were obtained by constrained energy minimization on intermediated configurations linearly interpolated between initial and final configurations. For each pathway, ten intermediate images were uniformly interpolated between the corresponding initial and final states. To investigate the effect of pre-existing lattice strain on the deformation energy landscape, the GSFE for the Fe_48_Mn_32_Co_10_Cr_10_ alloy was also calculated under applied tensile strains. Uniaxial tensile strains of 1% and 2% were applied perpendicular to the primary {111} slip plane prior to the fault creation, simulating a pre-deformed state. For each strain level (1%, 2%), a new set of 250 independent models was generated, and the GSFE calculation procedure described above was repeated. This approach allows for a systematic evaluation of how elastic strain influences the unstable SFE, intrinsic SFE, and the energy barriers associated with twinning.

## Supplementary information

Supplementary Information

Description of Additional Supplementary Files

Supplementary Data 1

Supplementary Movie 1

Transparent Peer Review file

## Source data

Source Data

## Data Availability

The data that support the findings of this study are available from the corresponding authors upon request. Source data are provided with this paper.
