# Supplementary material for: Inhomogeneity of negative enthalpy enables excellent mechanical properties in nanosized single crystals of multi-principal element alloys
Source: Nat Commun. 2026 Oct 11;17:11541. doi: 10.1038/s41467-026-78017-7 (PMC13748569; doi:10.1038/s41467-026-78017-7)
Supplement: Supplementary file 1 [file 41467_2026_78017_MOESM1_ESM.pdf]

Inhomogeneity of negative enthalpy enables excellent mechanical properties in nanosized single crystals of multi-principal element alloys

Yan Ma[1,#], Zhanxin Wang[1,#], Jiabao Zhang[1,#], Ning Xu[2], Zhimeng Duan[1], Dongwei Li[1], Ziqi Wu[3], Xiaoxue Chang[4], Ruiwen Shao[3], Tingyang Huang[5], Xiayan Wang[6], Zaicheng Sun[6], Changtian Wang[1], Zibing An[7], Chengpeng Yang[8,*], Jianfei Zhang[9,*], Xiaozhou Liao[10], Lihua Wang[1,*] & Xiaodong Han[1,7,*]

[1] Beijing Key Lab of Microstructure and Property of Advanced Materials, College of Materials Science & Engineering, Beijing University of Technology, Beijing, 100124, China

[2] Branch of Iron and Steel Research Institute, Ansteel Beijing Research Institute Co., Ltd., Beijing, 102211, China

[3] School of Medical Science and Engineering, Beijing Institute of Technology, Beijing, 100081, China

[4] Analysis & Testing Center, Beijing Institute of Technology, Beijing, 100081, China

[5] School of Economics, Beijing Wuzi University, Beijing 101149, China

[6] State Key Laboratory of Materials Low-Carbon Recycling, College of Chemistry and Life Science, Beijing University of Technology, Beijing, 100124, China

[7] Department of Materials Science and Engineering, Southern University of Science and Technology, Shenzhen, 518055, China

[8] Key Laboratory of Multifunctional Nanomaterials and Smart Systems, Division of Advanced Materials, Suzhou Institute of Nano-Tech and Nano-Bionics, Chinese Academy of Sciences, Suzhou, 215123, China

[9] Beijing Key Laboratory of Multimedia and Intelligent Software Technology, Beijing Artificial Intelligence Institute, Faculty of Information Technology, Beijing University of Technology, Beijing, 100124, China

[10] School of Aerospace, Mechanical and Mechatronic Engineering, The University of Sydney, Sydney, New South Wales, 2006, Australia

[#] These authors contributed equally: Yan Ma, Zhanxin Wang, Jiabao Zhang

[*] Corresponding authors: Chengpeng Yang; Jianfei Zhang; Lihua Wang; Xiaodong Han

Email: yang15763948783@163.com; JFZhang@bjut.edu.cn; wlh@bjut.edu.cn; xdhan@bjut.edu.cn.

This PDF file includes:

Supplementary text
Supplementary Figures 1 to 19
Supplementary References

Supplementary text

Quantitative in-situ tensile TEM system. The newly developed double-tilt in situ TEM device comprises an integrated capacitance signal processing module, actuator, MEMS chip and specimen holder, as shown in Supplementary Fig. 1. The integrated capacitance signal processing module can acquire and record the strain-stress in real time with high stability and precision. The actuator provides an external force on the sample. The MEMS chip developed in this work integrates two capacitive displacement sensors on a single chip platform, which enables the acquisition of stress-strain data (see details in stress-strain measurement section below). As shown in Supplementary Fig. 1b, the developed specimen holder in this work has double tilt ability (±20° about both the α-axis and β-axis), allowing the electron beam to be accurately aligned along low-index zone axes of the specimen and thus enabling atomic-resolution imaging. This capability is key for in situ observation of the deformation process at the atomic-scale, and simultaneously acquiring stress-strain data. Owing to constraints such as actuator size and the limited pole-piece gap in TEM, previously designed specimen holders were only with single tilt ability, making it difficult to obtain atomic-scale imaging. The dual-sensor integrated MEMS chip and double tilt ability developed in the current study allow synchronized atomic-scale observation and quantitative strain-stress readout.

In-situ experimental MEMS chip. The in situ tensile test was conducted at a nominal strain rate of ~10^{-4} s^{-1}. As shown in Supplementary Fig. 1a, the MEMS chip adopts a semi-open C-shaped architecture, with the specimen region positioned between the two displacement sensors. Each sensor employs a parallel-plate capacitive configuration (Supplementary Fig. 1b): during tensile loading, the mass block drives the movable plate to move, thereby modulating the gap between the movable and fixed electrodes. The onboard signal-processing module within the holder performs amplification, demodulation, and acquisition of the capacitance signal (Supplementary Fig. 1c), enabling real-time displacement readout.

As shown in Supplementary Fig. 2c, the quantitative mechanical MEMS chip was fabricated from a sandwich-structured SOI (silicon-on-insulator) wafer consisting of a Si basal layer, a buried SiO_2 layer, and a p-type device layer (resistivity 1-10 Ω·cm). The buried oxide electrically isolates the device layer from the basal layer, ensuring stable and reliable electrical readout. Microfabrication began with dry etching to define the differential capacitive displacement sensors, force-sensing beam, interconnects, and other microstructural units in the device layer, with the load beam axis aligned along the <110> crystallographic direction. The basal layer and buried oxide were then removed by wet etching to release the suspended structures. To reduce interconnect resistance and improve signal transmission, a thin Au film was subsequently deposited on the interconnects by sputtering, enhancing conductivity and electrical contact stability.

After fabrication, the MEMS chip was assembled onto the integrated stage shown in Supplementary Fig. 1b, and capacitance-to-displacement calibration was performed inside the TEM using the digital double-tilt in situ holder. During actuator-driven motion of the proof mass, the relative displacement of the movable electrode with respect to the fixed electrode was directly measured from TEM images, while the corresponding capacitance output was recorded synchronously by the signal-processing module. Establishing a one-to-one correspondence between displacement and capacitance yields the capacitance-displacement calibration curve (Supplementary Fig. 2d). In subsequent in situ mechanical experiments, the real-time capacitance signals can be readily converted into displacement using this calibration, enabling quantitative determination and analysis of mechanical responses.

Stress-strain measurement. As shown in Supplementary Fig. 1, the MEMS chip integrates two capacitive displacement sensors, namely Sensor A and Sensor B. These two sensors, capable of measuring the axial displacement at the front and rear ends of the tested specimen, were integrated at both sides of the gauge section. This setup enables real-time acquisition of the strain and stress of the specimen during tensile loading. The lengths of the sample before and after tensile are denoted as l_1 and l_2, respectively. During tensile deformation, the displacements measured by Sensor A and Sensor B are x_a and x_b, respectively. Accordingly, the engineering strain ε of the specimen can be calculated using Eq. (1):

$$\varepsilon = \frac{l_2 - l_1}{l_1} = \frac{x_a - x_b}{l_1} \tag{1}$$

At the rear side of Sensor B, a double-ended fixed beam with stiffness k was integrated. During tensile loading, the beam undergoes flexural deformation, with a central deflection of x_b. The force applied to the beam, F_b, can be calculated according to Hooke's law as follows:

$$F_b = kx_b \tag{2}$$

Because the central load on the doubly clamped beam, F_b, and the tensile load experienced by the specimen, F, constitute an action-reaction pair, they are equal in magnitude according to Newton's third law (i.e., $F = F_b$). After determining the specimen's initial width w_s and initial thickness d_s from electron microscopy measurements, the engineering stress during tensile loading can be obtained using Eq. (3):

$$\sigma = \frac{F_s}{w_s d_s} = \frac{kx_b}{w_s d_s} \tag{3}$$

It should be noted that, because Eqs. (1) and (3) are formulated using the specimen's initial dimensions; the stress and strain obtained in this work correspond to engineering stress and engineering strain, respectively.

The length l, width w, and thickness d of the doubly clamped beam can be precisely defined by semiconductor microfabrication (etching) processes; therefore, the beam stiffness k can be calculated using Eq. (4):

$$k = \frac{2Ew^3 d}{l^3} \tag{4}$$

where E denotes the axial Young's modulus of the clamped beam.

The platform's accuracy was validated by testing a FIB-prepared <002> single-crystal Cu standard sample, as shown in Supplementary Fig. 3. The measured Cu <002> Young's modulus from the stress-strain curve was 67.718 GPa, which deviates by only 1.8% from the first-principles value of 66.6 GPa [1], with the minor error attributed to the FIB-induced surface amorphous layer and sample geometry uncertainties.

Tensile deformation simulations. To unravel the underlying deformation mechanisms, MD simulations of the $Fe_{48}Mn_{32}Co_{10}Cr_{10}$ MPEA were performed using the Large-scale Atomic/Molecular Massively Parallel Simulator (LAMMPS) [2]. The simulation cell contained 510,528 atoms arranged in an orthogonal geometry with dimensions of 48 nm (length) × 16 nm (width) × 8.4 nm (thickness), as shown in Supplementary Fig. 8. The crystallographic orientation of the model was aligned with the experimental system that show in Fig. 2. Atomic interactions were described using the Mie potential parameterized by Gröger et al. [3], which has been rigorously validated for capturing the effects of compositional randomness on defect properties in FCC high-entropy alloys, including stacking fault energies, point defect formation energies, and dislocation migration barriers. The simulations employed a time step of 1 fs. Free surfaces were imposed in all three dimensions to replicate the surface effects relevant to nanoscale specimens. The system was initially energy-minimized using the conjugate gradient algorithm, followed by a 200 ps equilibration in the NVT ensemble at 300 K to achieve thermodynamic stability before the onset of mechanical loading. Three atomic layers at the top and bottom of the model (along the length direction) were then fixed as rigid loading platens. Uniaxial tensile deformation was applied by imposing opposing velocities of v= ±0.024 Å/ps on the rigid layers, corresponding to an engineering strain rate of approximately 1.0×10^8 s^{-1}. The corresponding stress-strain curve is shown in Supplementary Fig. 9. The tensile simulation was conducted for 3 ns to adequately capture the progression of plastic deformation and the associated microstructural evolution. Microstructural evolution was visualized using the Open Visualization Tool (OVITO) [4]. The common neighbor analysis (CNA) method was employed to identify local crystal structures. Atoms were colored according to their identified structure: green for FCC, red for HCP, and white for disordered or interfacial atoms.

Mixing enthalpy. According to thermodynamic theory, in the MPEA systems, higher configurational entropy results in ideal entropy alloys (elements disordered and random

distribution). In an ideal entropy alloy system, the mixing enthalpy (H_{mix}, see Methods for the calculation of mixing enthalpy) is expected to be approximately zero. However, the actual mixing enthalpy values differ among the elements, as illustrated in Supplementary Fig. 19, which shows the mixing enthalpy between the pairs of Fe, Mn, Co, and Cr. The chemical affinity between pairs of atoms with negative mixing enthalpy fosters the formation of strong atomic bonds, as indicated by the high bonding energies. This facilitates the formation of atomic-scale fluctuations in chemical composition. During the deformation of the MPEA, dislocations traversing these fluctuation regions cause the breaking and reformation of various atomic bonds, potentially resulting in unconventional deformation behavior.

Supplementary Figures

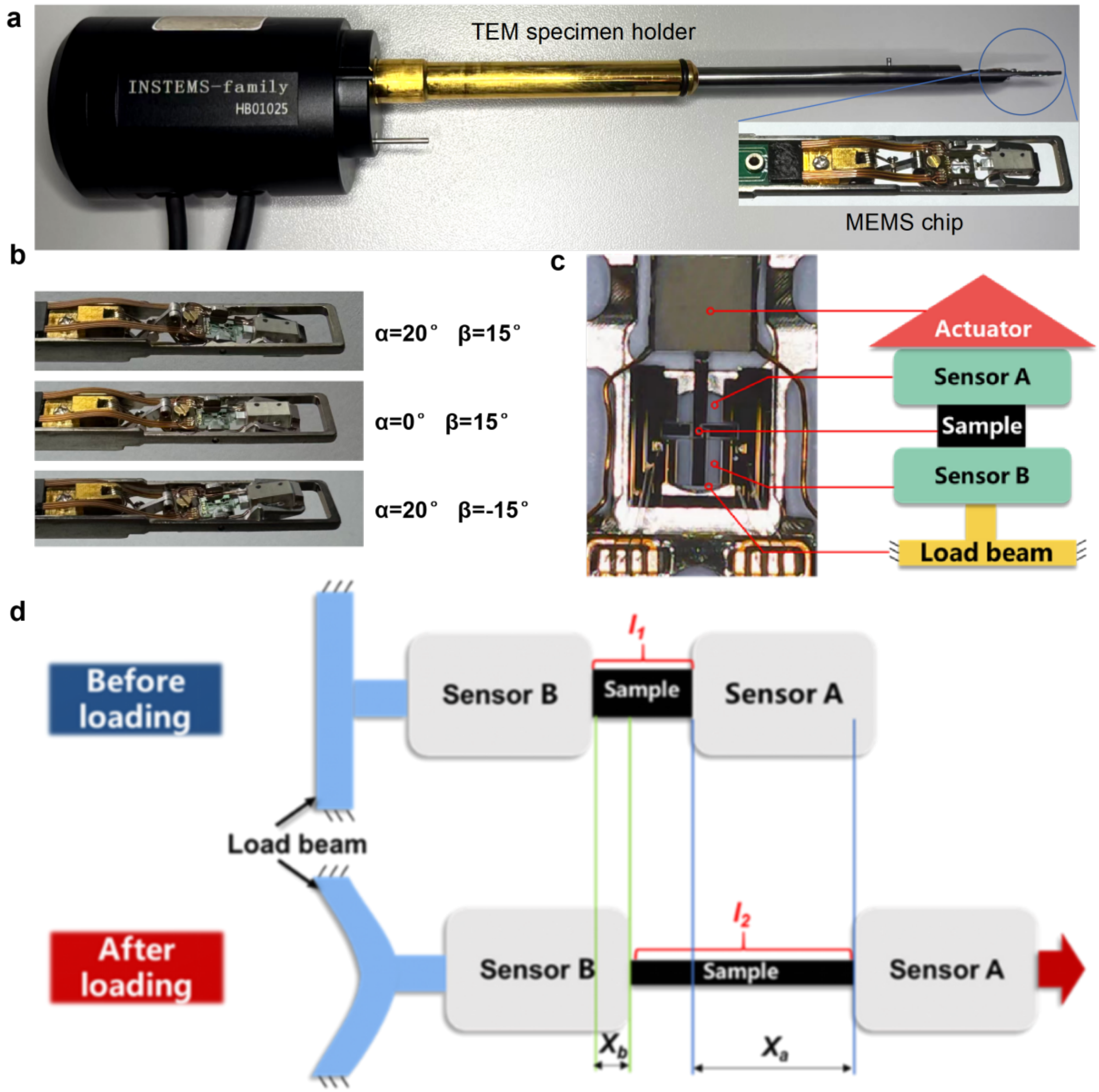

Fig. 1 | Quantitative in situ TEM testing device. (a) In-situ tensile mechanical property quantification TEM sample holder. (b) Three-dimensional simulation of the double-tilt angles. (c) Image of the integrated MEMS tensile device. (d) Schematic illustration of the quantitative in situ mechanical MEMS chip.

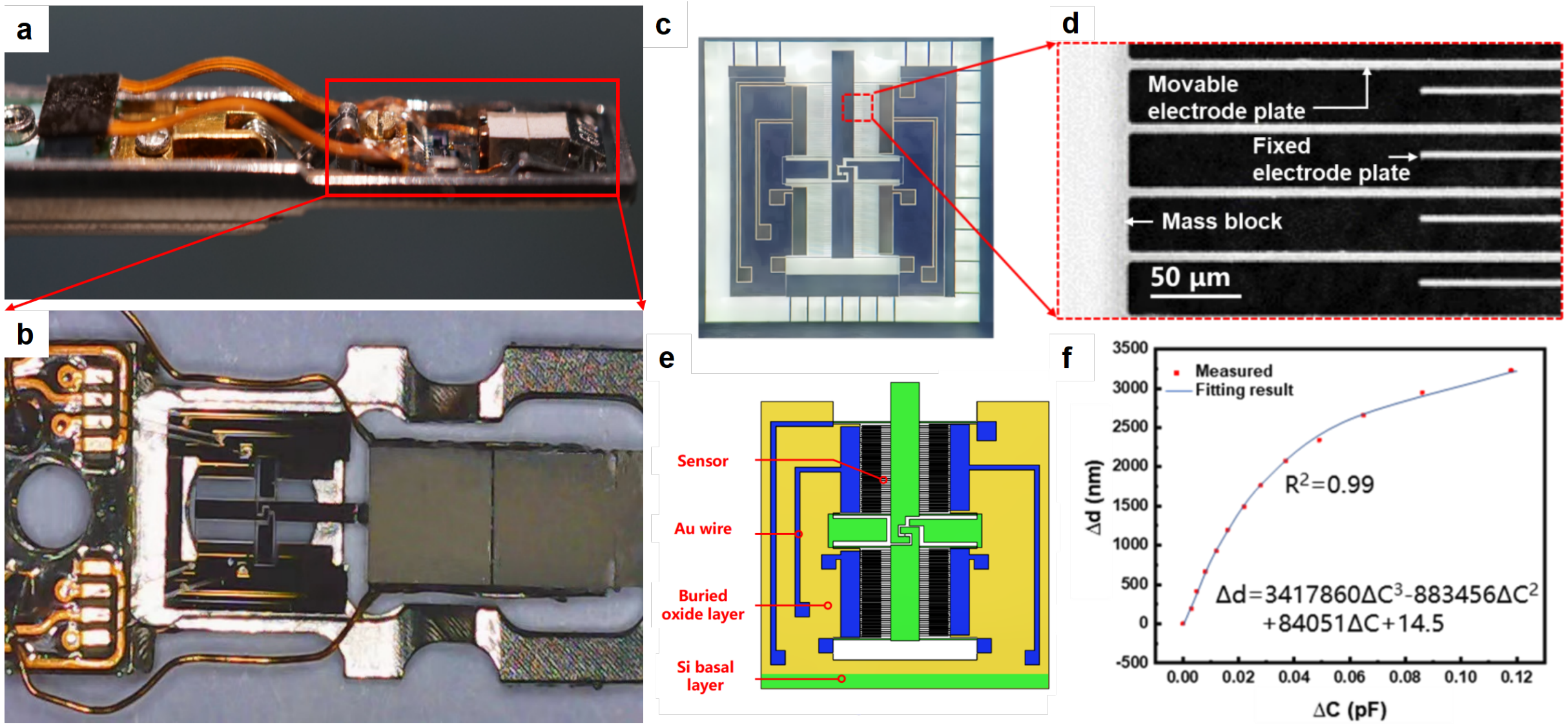

Fig. 2 | Details of the tensile testing device. (a, b) Side view and top view photographs of the in-situ tensile device. (c) Image of the quantitative in situ mechanical MEMS device. (d) Enlarged view of the capacitive sensor. (e) Exploded view of the mechanically quantitative MEMS chip. (f) Calibration curve relating the displacement of the movable electrode to the capacitance variation.

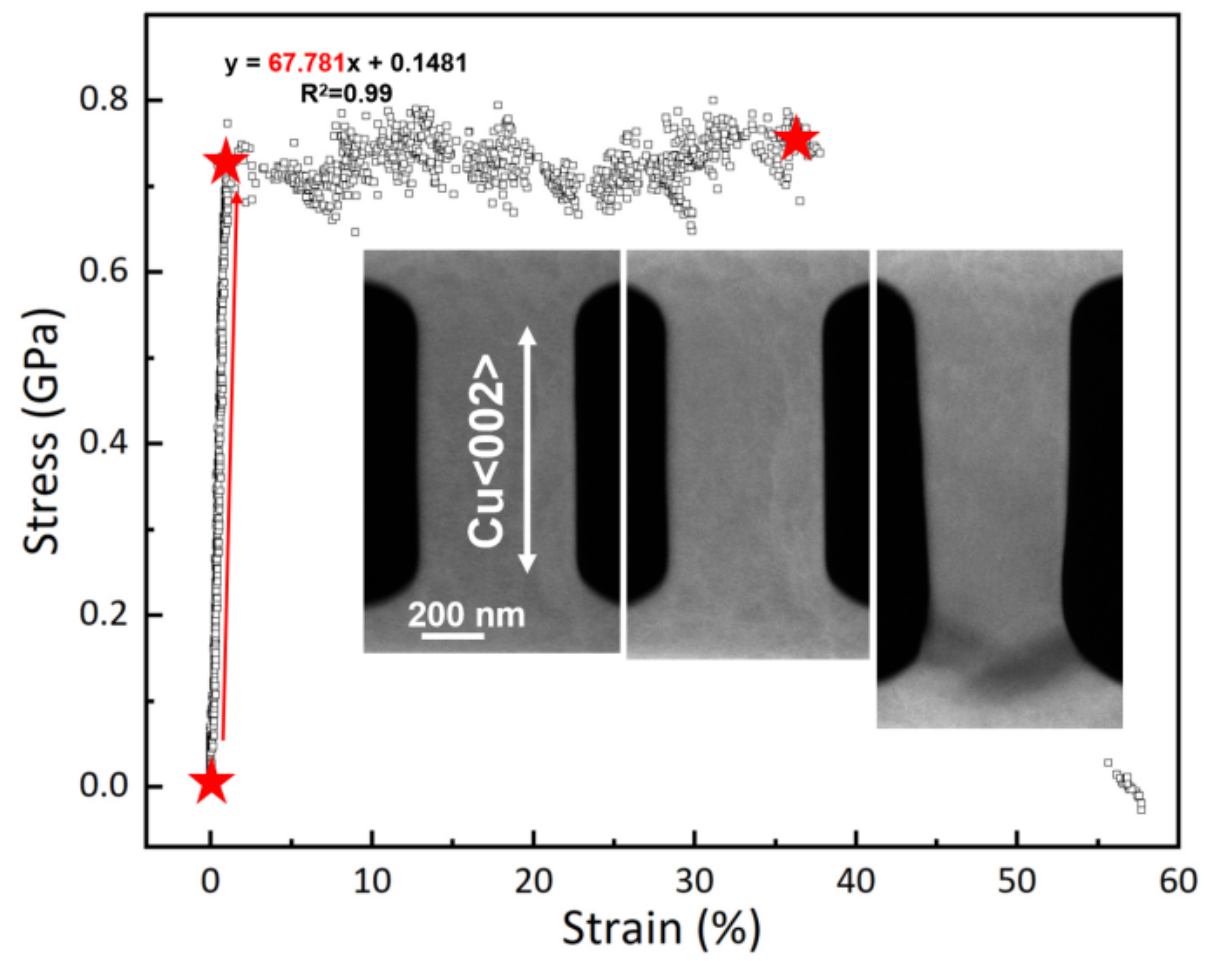

Fig. 3 | Tensile stress-strain curve of single crystal Cu. The elastic modulus of a Cu single crystal with the <002> orientation was measured.

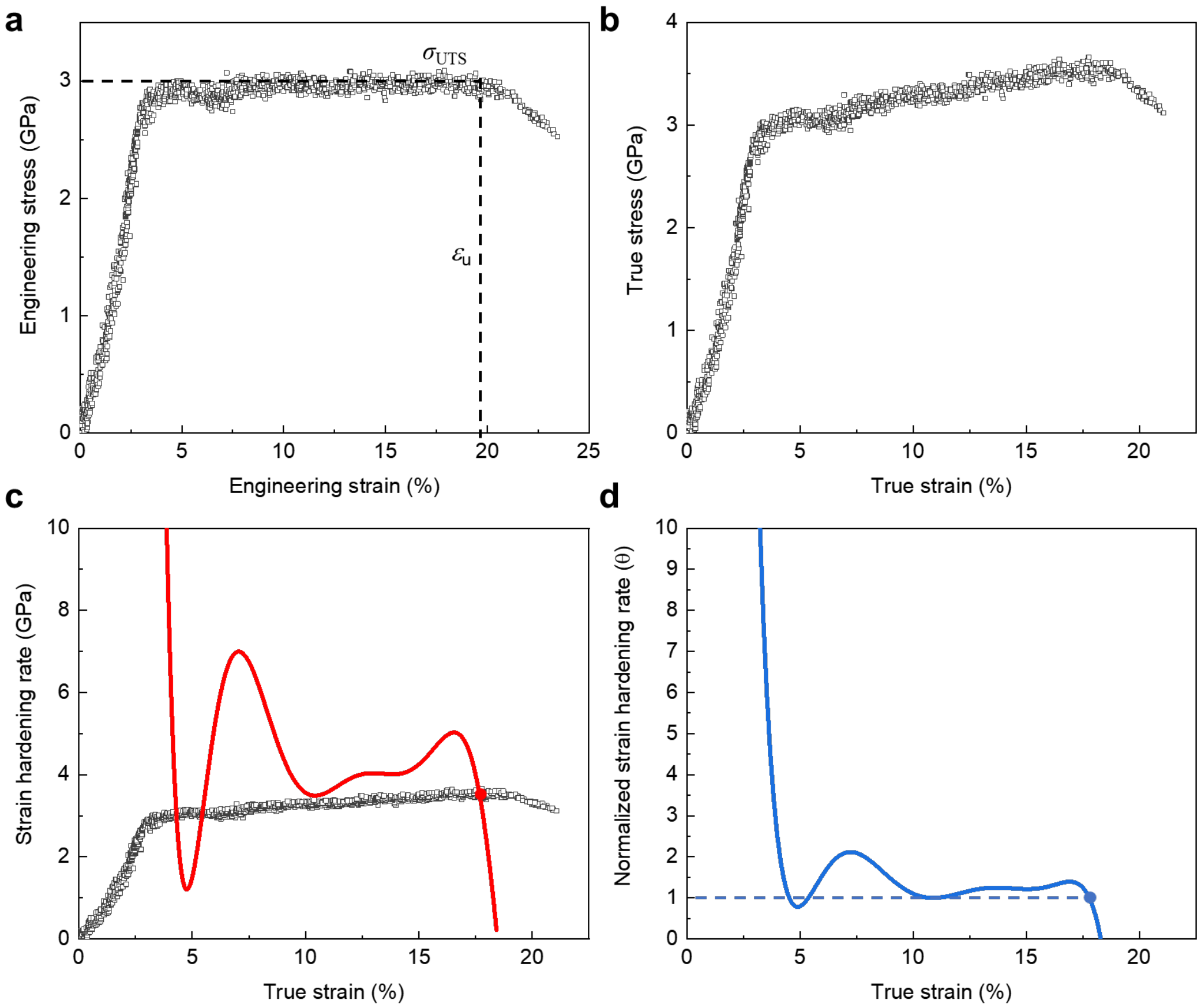

Fig. 4 | Mechanical properties of the NSC (a) Engineering stress-strain curve of the NSC; (b) True stress-strain curve of the NSC; (c) Strain hardening rate curve of the NSC; (d) Normalized strain-hardening rate curve of the NSC. Source data are provided as a Source Data file.

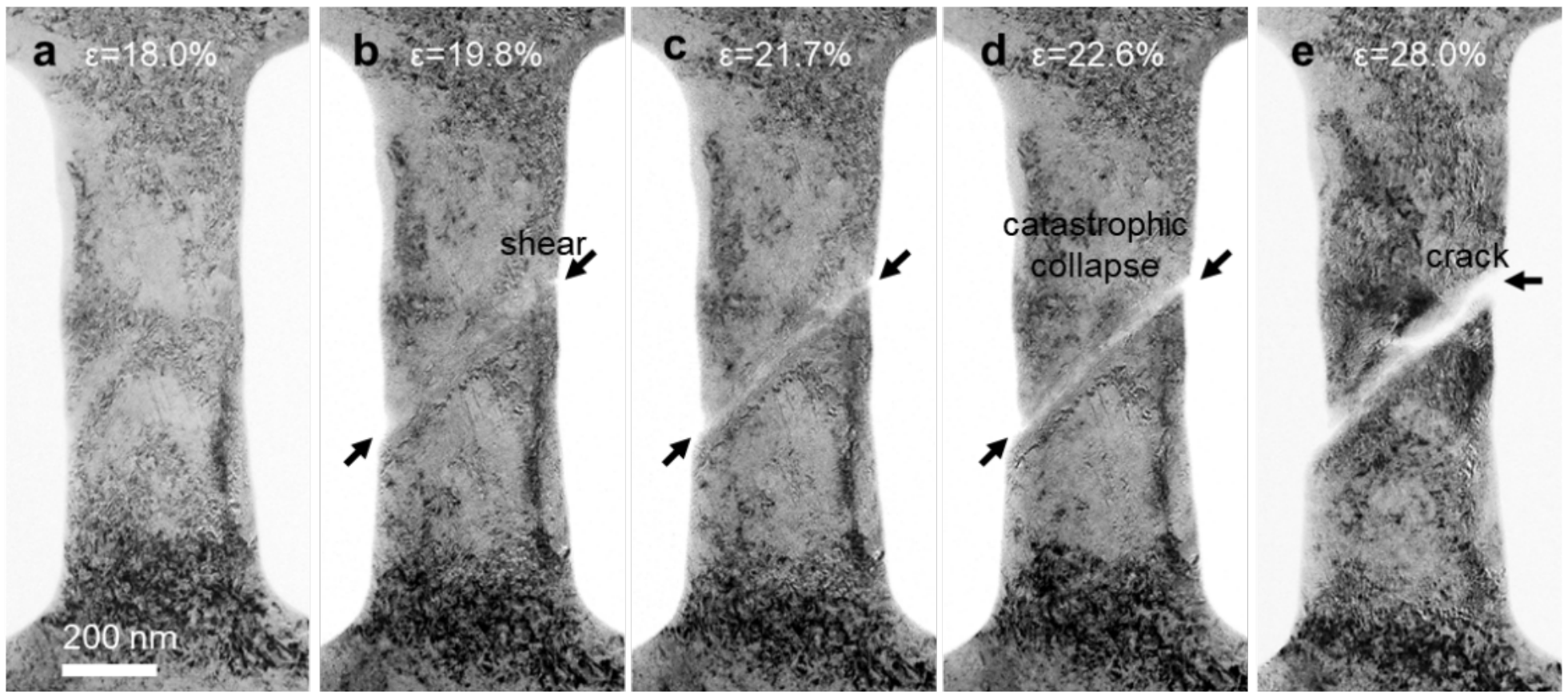

Fig. 5 | In situ TEM images revealing dislocation traversal and the resulting shear instability in the NSC sample before fracture. (a-e) The shear fracture process of the NSC sample is demonstrated.

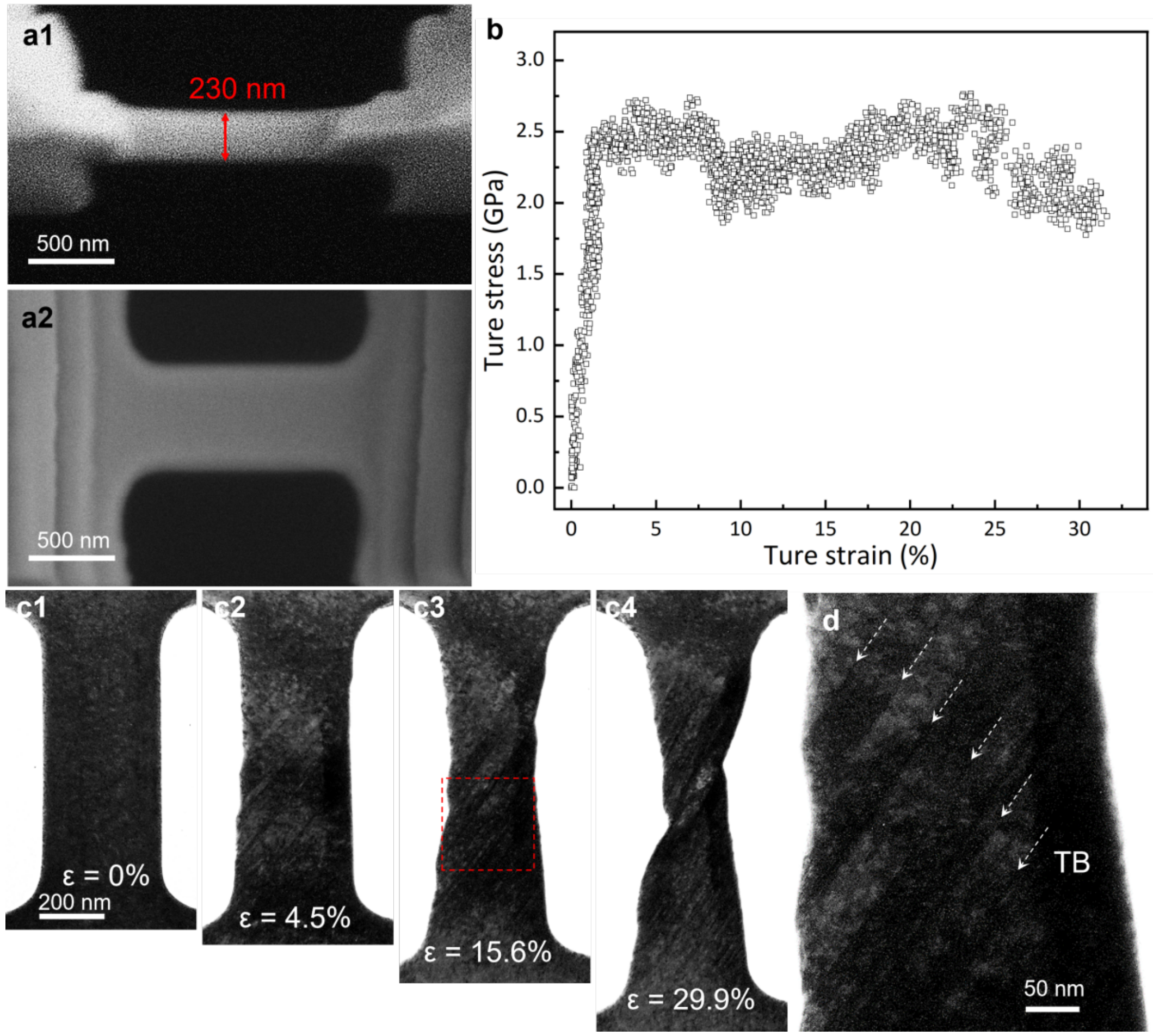

Fig. 6 | In-situ TEM experiment of NSC with a thickness of 230 nm. (a) Front and side views of the NSC sample in SEM. (b) True stress-strain curve. (c) Bright-field images during in-situ tensile testing. (d) Enlarged image of the area outlined in red in **c**. Source data are provided as a Source Data file.

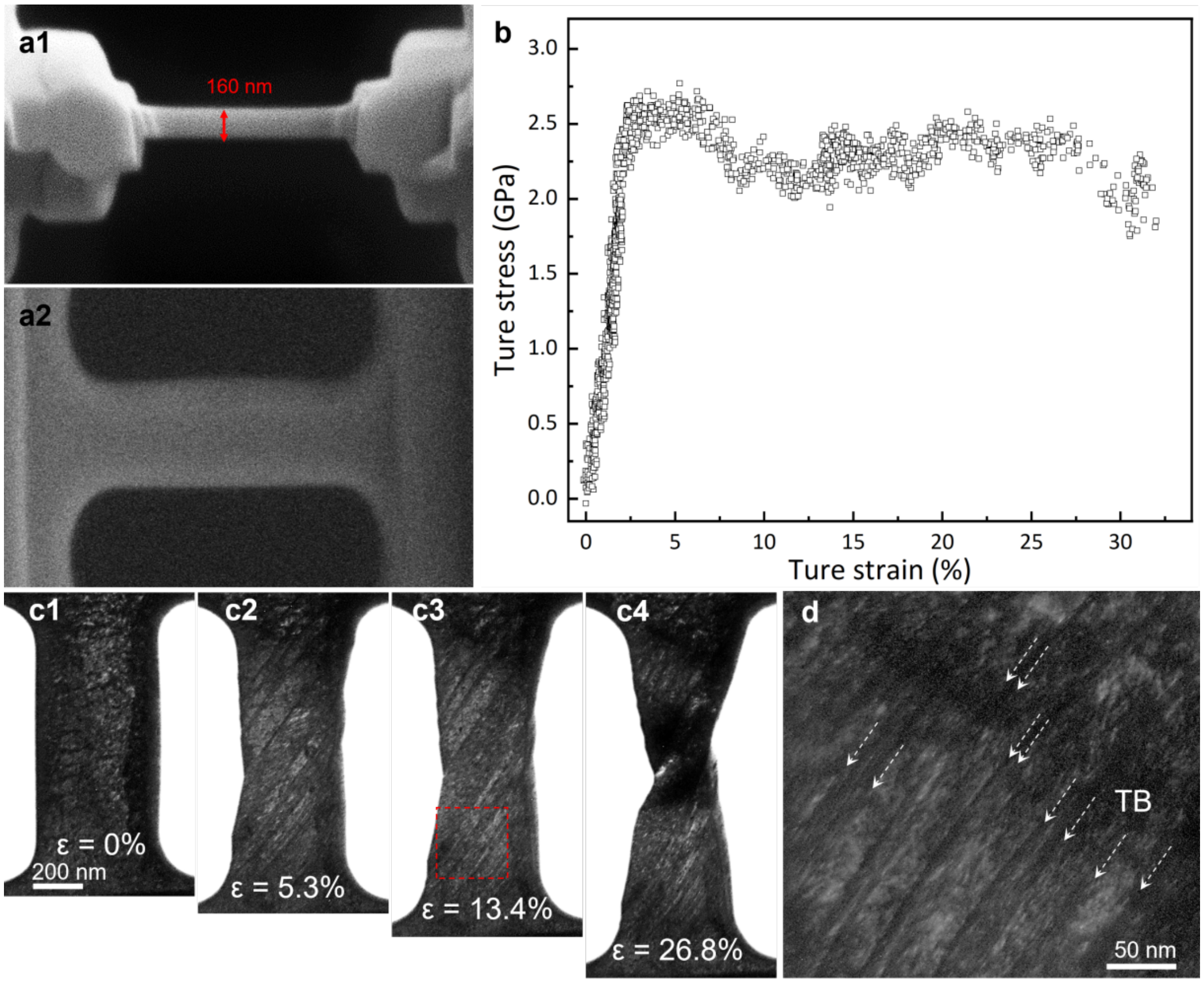

Fig. 7 | In-situ TEM experiment of NSC with a thickness of 160 nm. (a) Front and side views of the NSC sample in SEM. (b) True stress-strain curve. (c) Bright-field images during in-situ tensile testing. (d) Enlarged image of the area outlined in red in **c**. Source data are provided as a Source Data file.

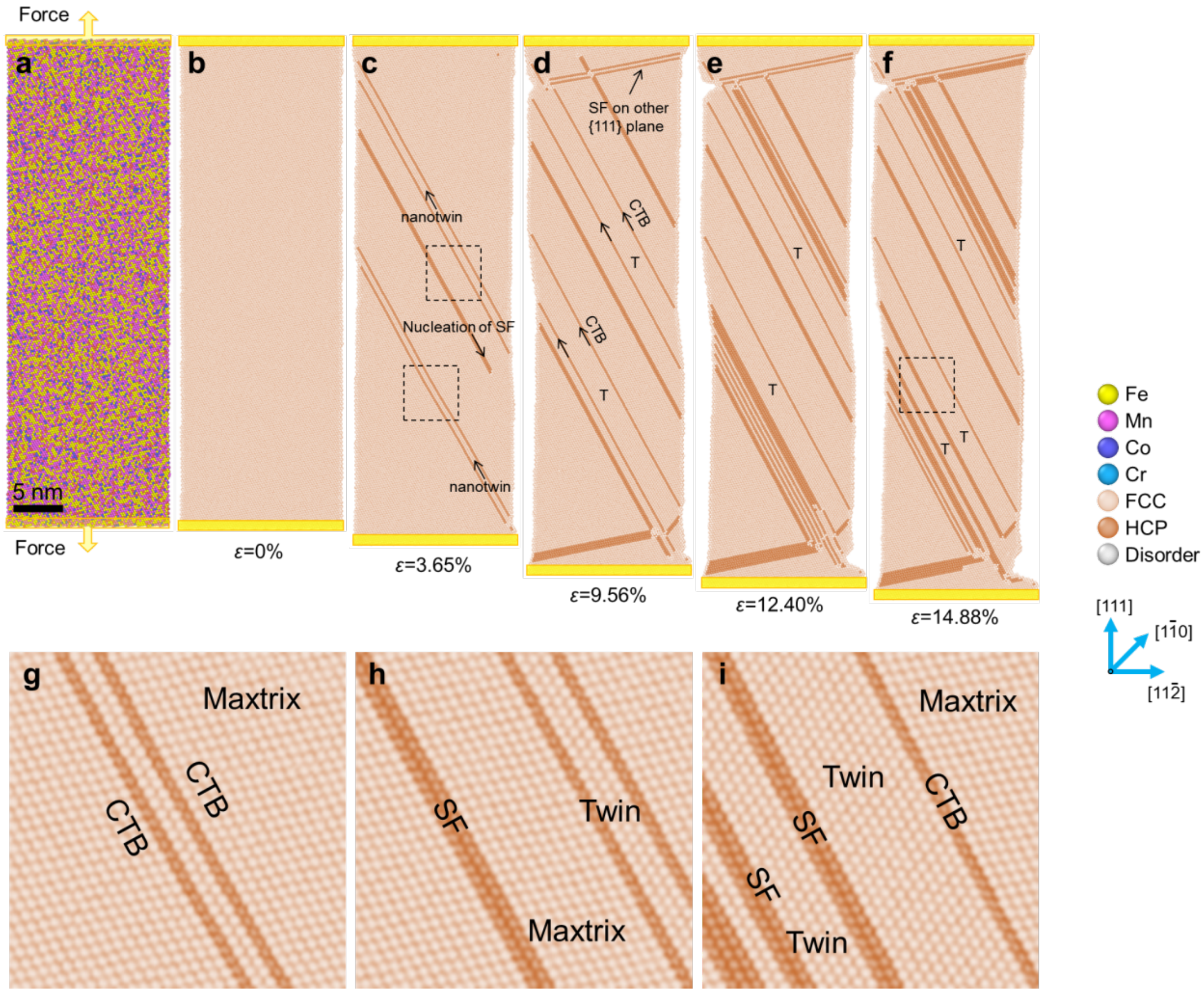

Fig. 8 | The molecular dynamics simulation of $Fe_{48}Mn_{32}Co_{10}Cr_{10}$ MPEA during in-situ tensile deformation. (a) After model relaxation, the distribution of the Fe, Mn, Co, and Cr elements. (b) to (f) The tensile process from 0% to 14.88% strain is displayed. (g) to (i) Magnified details of SF and CTB. Green atoms represent the face-centered cubic structure. Red atoms represent the hexagonal close-packed structure, which can be considered as SFs formed by the emission of dislocations. During deformation, dislocations are emitted on different {111} planes, leading to the microstructure of non-adjacent SFs. (g) to (f) Enlarged image of the area outlined in red in c to e, revealing details of CTB and SF.

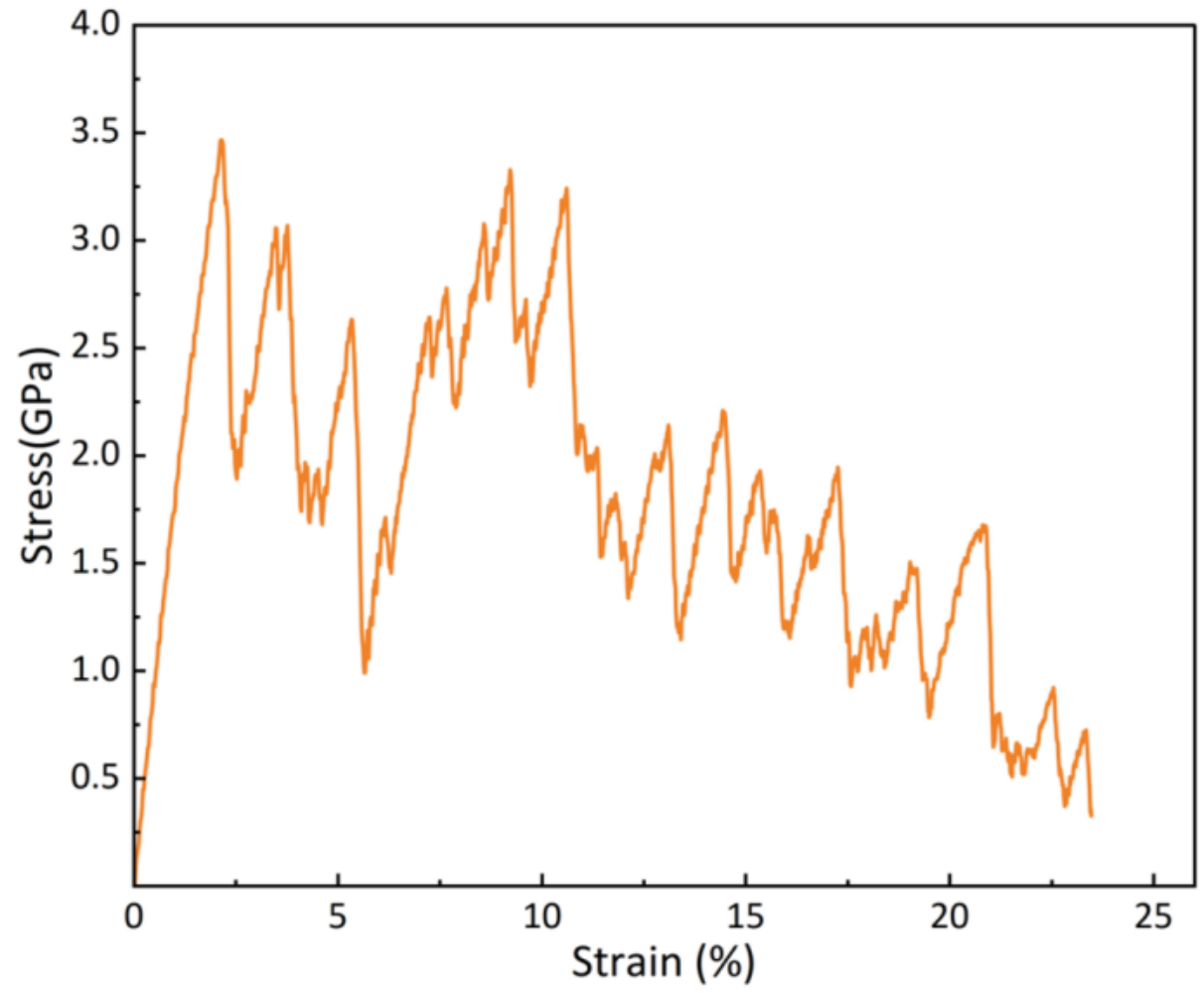

Fig. 9 | MD simulation of the stress-strain curve for NSC. The mechanical response of the NSC during tensile deformation simulated by MD is presented. Source data are provided as a Source Data file.

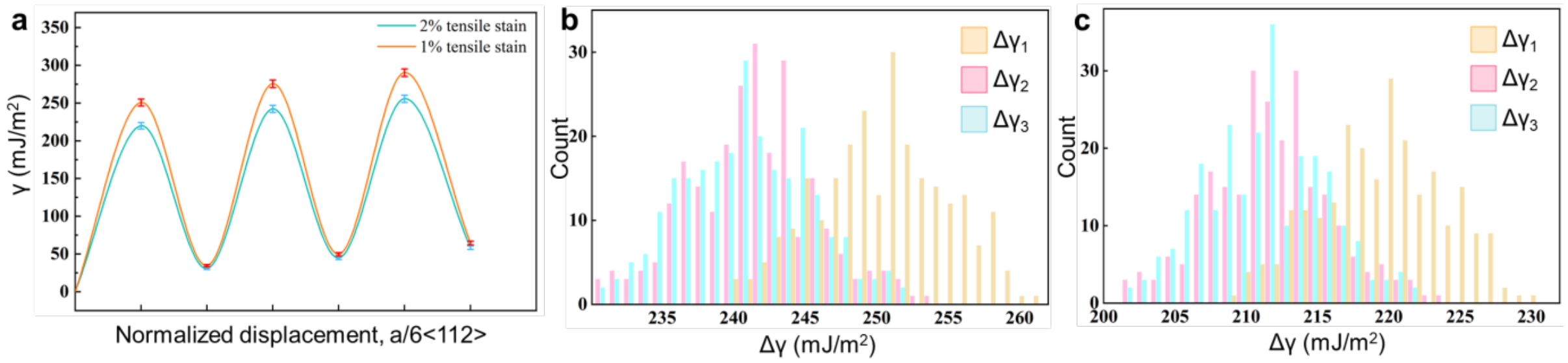

Fig. 10 | GSFE of the NSC under different tensile strain conditions. (a) GSFE curves under 1% and 2% tensile strains of the NSC. (b) Distribution of unstable SFE barriers under 1% tensile strains. (c) Distribution of unstable SFE barriers under 2% tensile strains. Source data are provided as a Source Data file.

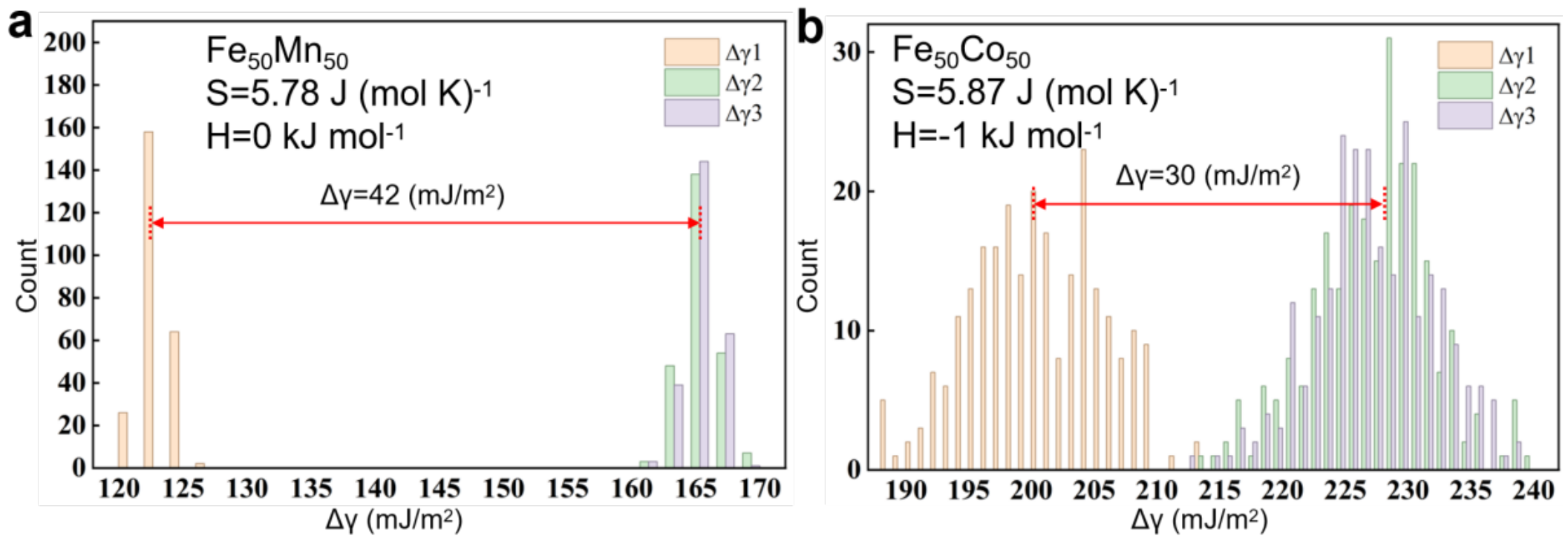

Fig. 11 | Direct comparison of SFE between $Fe_{50}Mn_{50}$ and $Fe_{50}Co_{50}$ binary alloys. Distribution of unstable SFE barriers for (a) $Fe_{50}Mn_{50}$, (b) $Fe_{50}Co_{50}$. Source data are provided as a Source Data file.

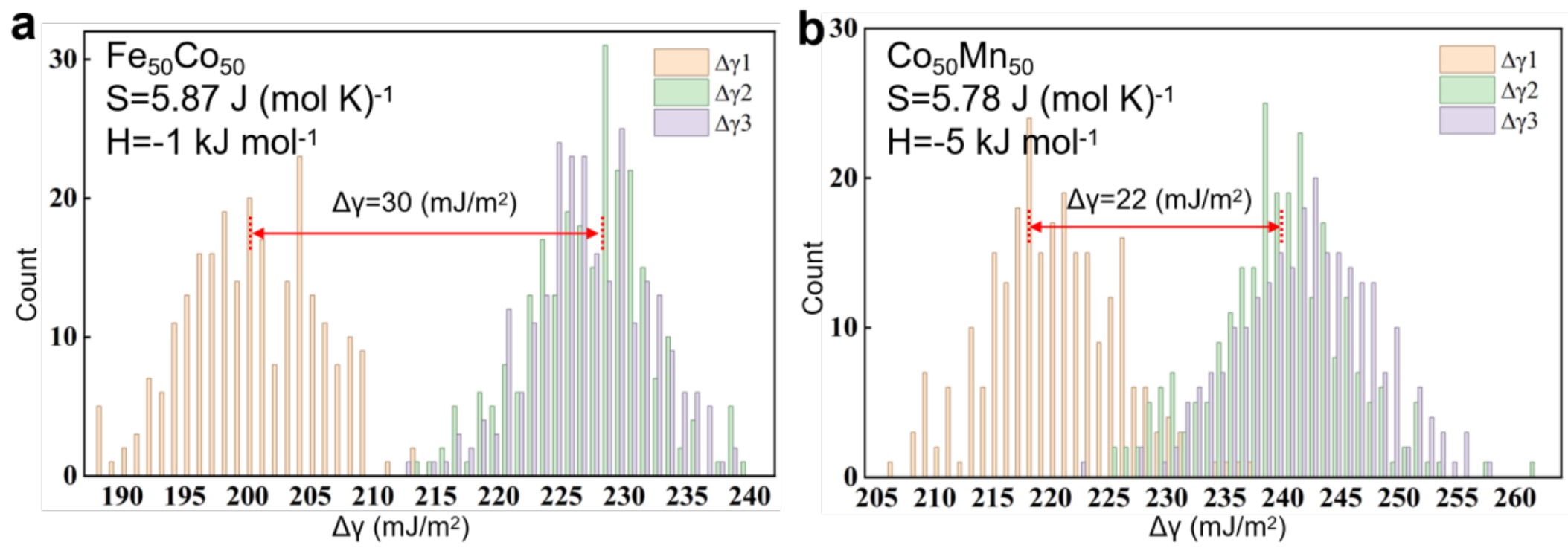

Fig. 12 | Direct comparison of SFE between $Fe_{50}Co_{50}$ and $Co_{50}Mn_{50}$ binary alloys. Distribution of unstable SFE barriers for (a) $Fe_{50}Co_{50}$, (b) $Co_{50}Mn_{50}$. Source data are provided as a Source Data file.

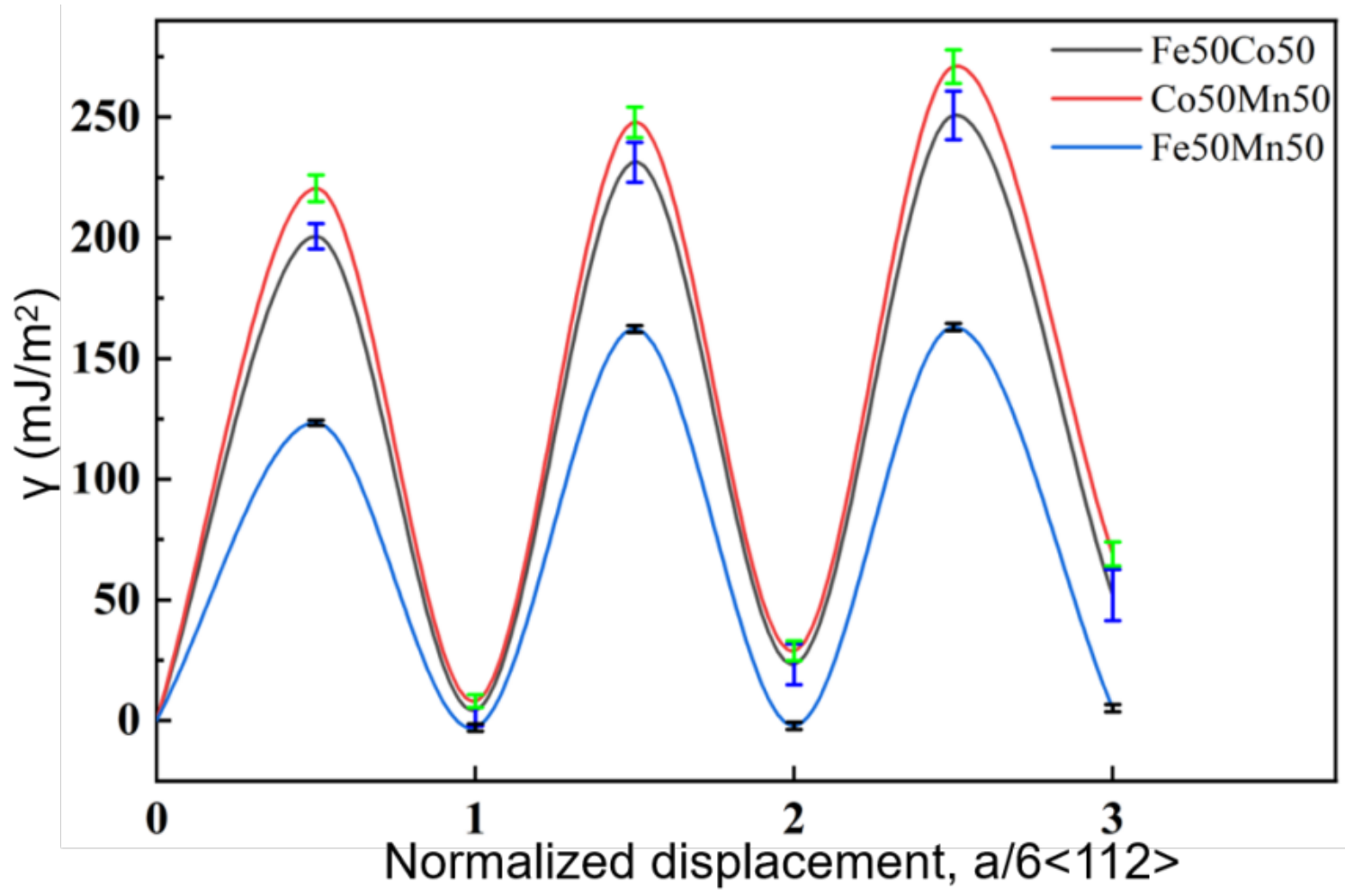

Fig. 13 | Direct comparison of GSFE among different binary alloys. GSFE curves of the $Fe_{50}Mn_{50}$, $Co_{50}Mn_{50}$ and $Fe_{50}Co_{50}$ along the twin formation pathway. Source data are provided as a Source Data file.

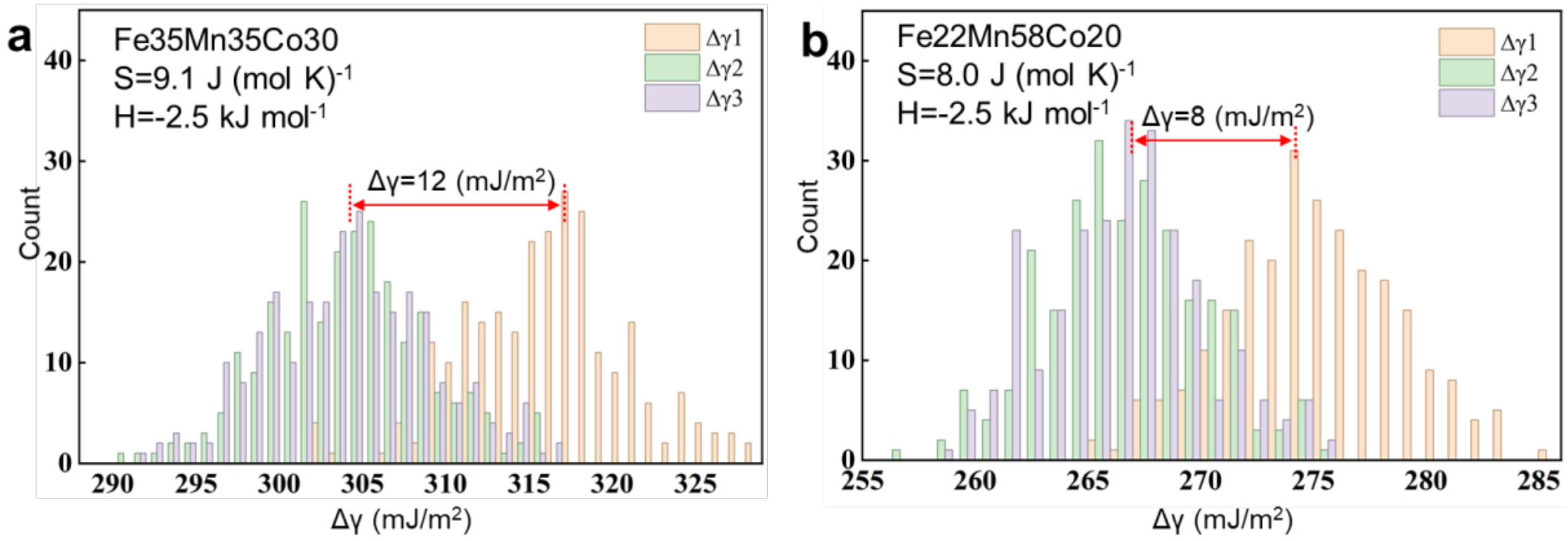

Fig. 14 | Direct comparison of SFE between $Fe_{35}Mn_{35}Co_{30}$ and $Fe_{22}Mn_{58}Co_{20}$ ternary alloys. Distribution of unstable SFE barriers for (a) $Fe_{35}Mn_{35}Co_{30}$, (b) $Fe_{22}Mn_{58}Co_{20}$. Source data are provided as a Source Data file.

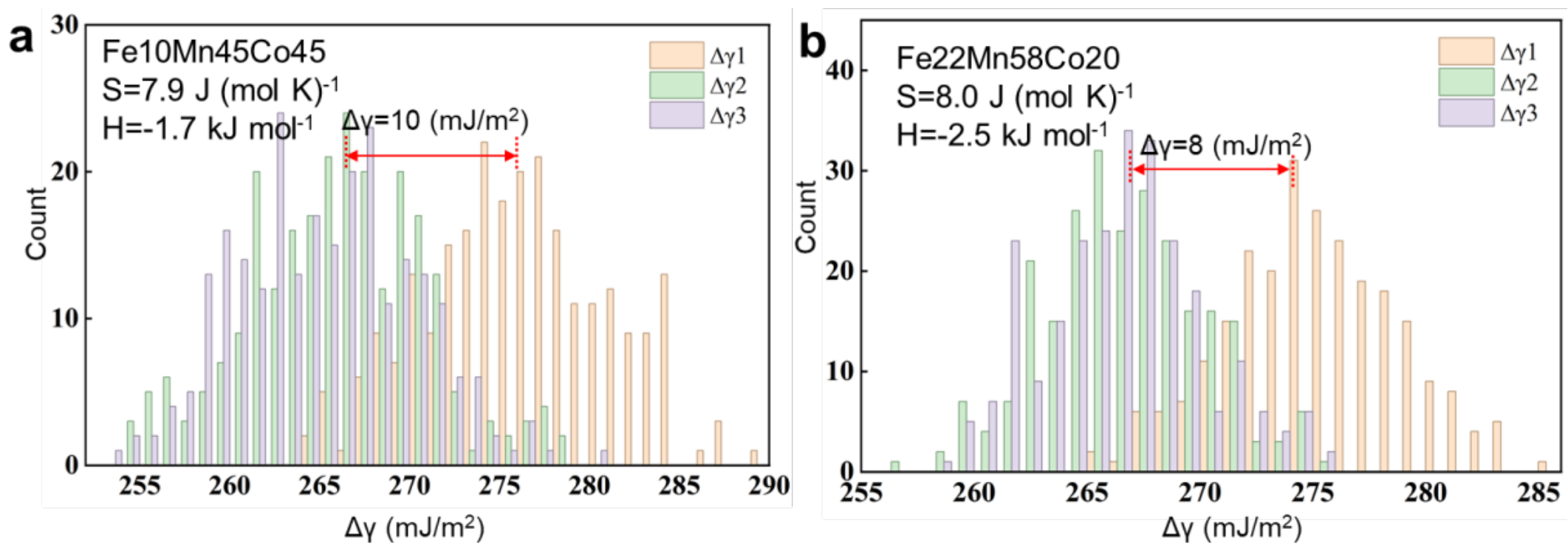

Fig. 15 | Direct comparison of SFE between $Fe_{10}Mn_{45}Co_{45}$ and $Fe_{22}Mn_{58}Co_{20}$ ternary alloys. Distribution of unstable SFE barriers for (a) $Fe_{10}Mn_{45}Co_{45}$, (b) $Fe_{22}Mn_{58}Co_{20}$. Source data are provided as a Source Data file.

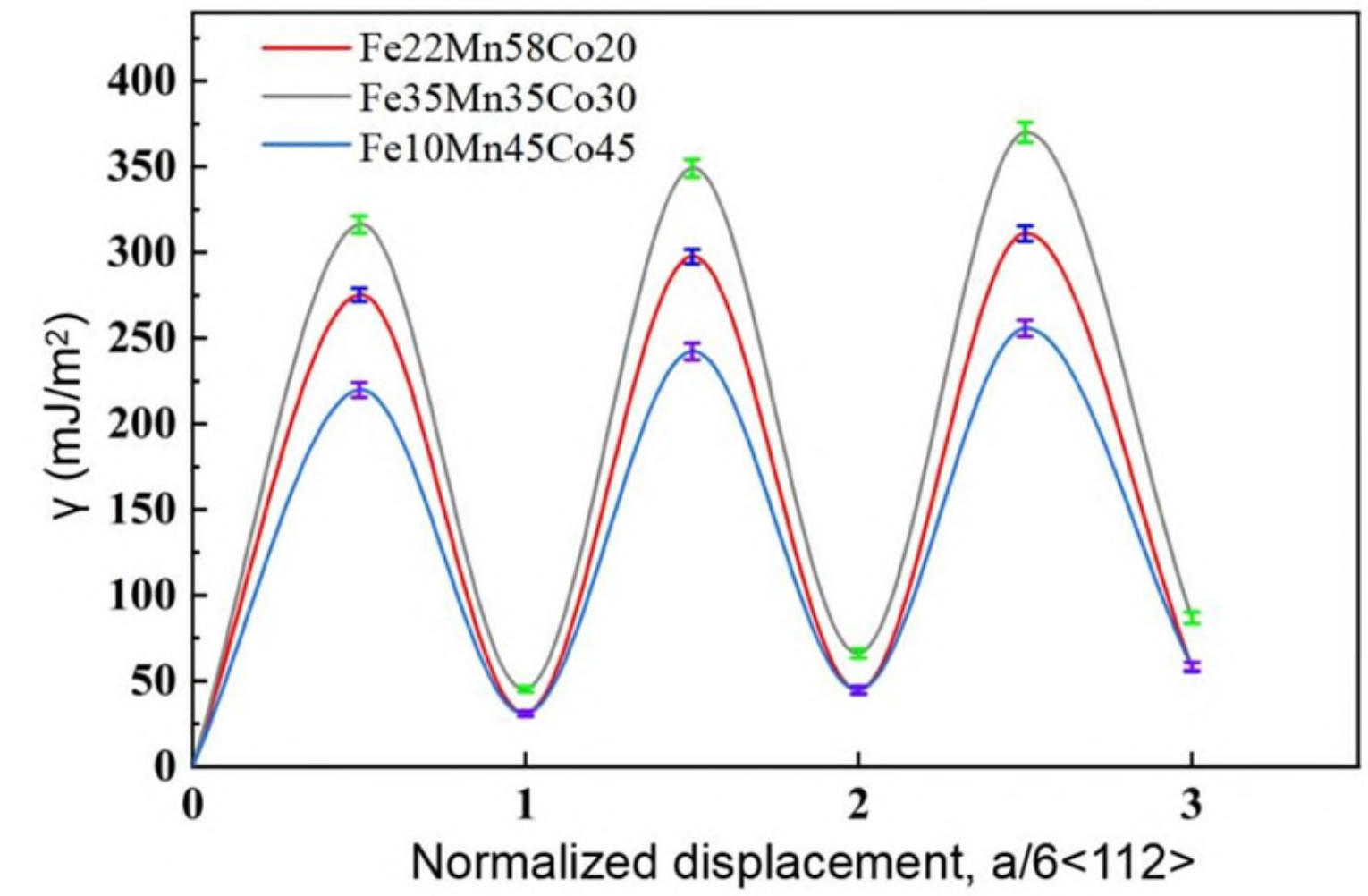

Fig. 16 | Direct comparison of GSFE among different ternary alloys. GSFE curves of the $Fe_{10}Mn_{45}Co_{45}$, $Fe_{22}Mn_{58}Co_{20}$ and $Fe_{35}Mn_{35}Co_{30}$ along the twin formation pathway. Source data are provided as a Source Data file.

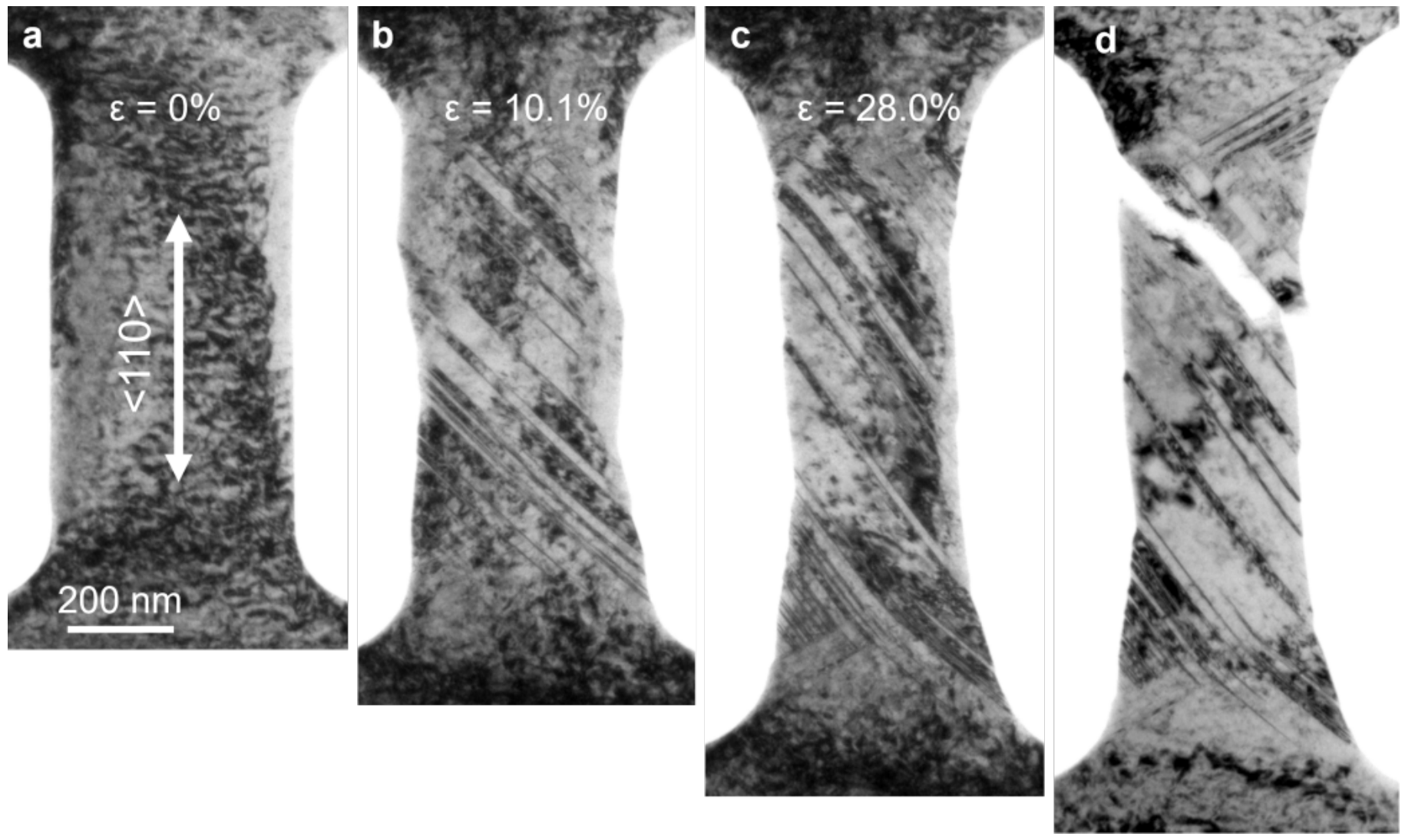

Fig. 17 | Bright-field TEM images of the NSC in the <110> direction during in-situ tensile deformation. (a-d) The deformation mechanism involving nanotwinning is demonstrated, which is similar to that observed in the main manuscript.

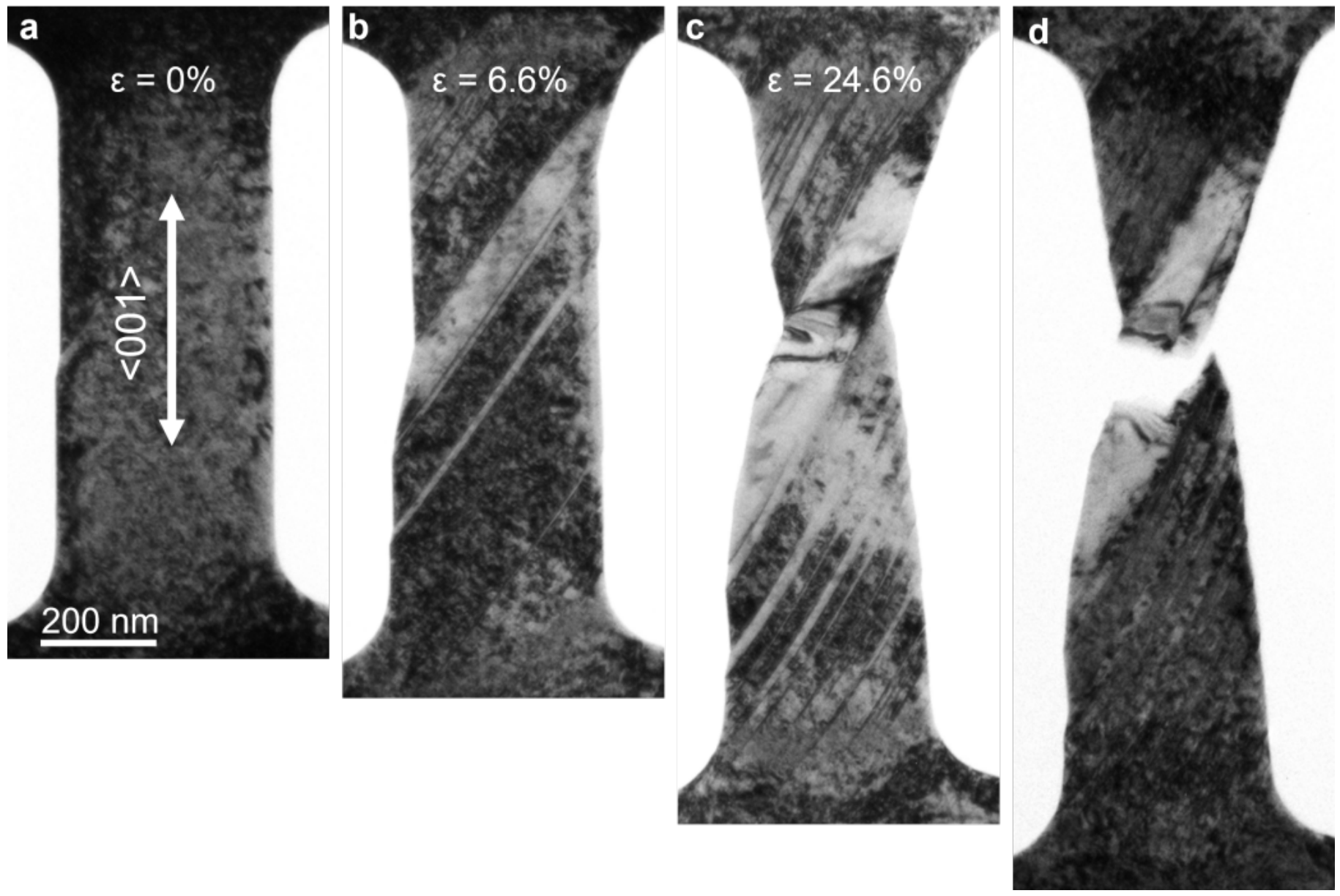

Fig. 18 | Bright-field TEM images of the NSC in the <001> direction during in-situ tensile deformation. (a-d) The deformation mechanism involving nanotwinning is demonstrated, which is similar to that observed in the main manuscript.

	Fe	Mn	Co	Cr
**Fe**	0	0	-1	-1
**Mn**	0	0	-5	2
**Co**	-1	-5	0	-4
**Cr**	-1	2	-4	0

Fig. 19 | Mixing enthalpy values of atomic pairs in the NSC alloy. The values of mixing enthalpy H_{mix} (kJ/mol) for atomic pairs between the elements with Fe, Mn, Co and Cr elements [5].

References

1. Levine, L. E. *et al.* X-ray microbeam measurements of individual dislocation cell elastic strains in deformed single-crystal copper. *Nat. Mater.* **5,** 619-622 (2006).
2. Plimpton, S. Fast parallel algorithms for short-range molecular dynamics. *J. Comput. Phys.* **117,** 1-19 (1995).
3. Gröger, R., Vitek, V., Dlouhý, A. Effective pair potential for random fcc CoCrFeMnNi alloys. *Modelling and Simulation in Materials Science and Engineering* **28,** 075006 (2020).
4. Stukowski, A. Visualization and analysis of atomistic simulation data with OVITO–the Open Visualization Tool. *Modelling and Simulation in Materials Science and Engineering* **18,** 015012 (2010).
5. Takeuchi, A., Inoue, A. Classification of Bulk Metallic Glasses by Atomic Size Difference, Heat of Mixing and Period of Constituent Elements and Its Application to Characterization of the Main Alloying Element. *Mater. Trans.* **46,** 2817-2829 (2005).
