# Supplementary material for: Inhomogeneity of negative enthalpy enables excellent mechanical properties in nanosized single crystals of multi-principal element alloys
Source: Nat Commun. 2026 Oct 11;17:11541. doi: 10.1038/s41467-026-78017-7 (PMC13748569; doi:10.1038/s41467-026-78017-7)
Supplement: Supplementary file 2 [file 41467_2026_78017_MOESM2_ESM.pdf]

Description of Additional Supplementary Files

File Name: Supplementary Data 1
Description: Molecular dynamics trajectories. The initial and final configurations.

File Name: Supplementary Movie 1
Description: Low magnification in-situ dynamic bright field TEM observation of MPEA NSC.
