# Supplementary material for: Inhomogeneity of negative enthalpy enables excellent mechanical properties in nanosized single crystals of multi-principal element alloys
Source: Nat Commun. 2026 Oct 11;17:11541. doi: 10.1038/s41467-026-78017-7 (PMC13748569; doi:10.1038/s41467-026-78017-7)
Supplement: Supplementary file 3 [file 41467_2026_78017_MOESM5_ESM.pdf]

nature portfolio

Peer Review File

Inhomogeneity of negative enthalpy enables excellent mechanical properties in nanosized single crystals of multi-principal element alloys

Corresponding Author: Professor Lihua Wang

Parts of this Peer Review File have been redacted as indicated to remove third-party material.

This file contains all reviewer reports in order by version, followed by all author rebuttals in order by version.

Version 0:

Reviewer comments:

Reviewer #1

(Remarks to the Author)
This manuscript reports the results of an experimental study on the plasticity of an FeMnCoCr-based nano-sized multi-principal element alloy (MPEA), revealed through in situ mechanical testing in a transmission electron microscope (TEM). As clearly described in the Introduction, MPEAs are known to exhibit unique and desirable mechanical properties compared with conventional pure metals. However, the underlying origin of such exceptional behavior still requires further investigation.

In this study, the authors observed that twin growth was considerably limited in the MPEA. Based on this observation, they proposed a broad distribution of stacking fault energies—arising from chemical inhomogeneity—as a primary cause. The experiments were well designed and conducted, and the overall logical flow leading to their conclusions is convincing. Nevertheless, several issues need be addressed to enhance the validity and credibility of their conclusions.

1. On page 4, line 112, the authors report the occurrence of necking during the tensile experiments. However, the stress–strain curve presented in Figure 2b does not exhibit any noticeable strain hardening. Typically, necking is closely associated with strain hardening, yet this connection is not apparent in the experimental results. Is there a specific explanation for this discrepancy?

2. In the paragraph beginning on page 5, line 156, the authors state that the intersection of full and partial dislocations with twin boundaries (TBs) is a critical deformation mode. However, describing this simply as an “intersection of dislocations with TBs” appears to refer to a resultant phenomenon rather than a deformation mechanism. Do the dislocations form within the TBs and subsequently glide toward the surface or adjacent TBs, or are they nucleated at the free surface and then glide into and become absorbed by the TBs?

3. The TEM bright-field images presented in Figures 2e–h do not convincingly serve as experimental evidence supporting the authors’ claims. The image quality appears too low to clearly resolve the relevant features, such as dislocations, making it difficult for this reviewer to interpret the microstructural details.

4. The reliability of the atomistic simulation data shown in Figure 6b requires further verification. While the authors primarily discuss the energy barriers in the coordinate–energy plot, it should be noted that the energies of the metastable configurations (i.e., those corresponding to normalized displacements 1, 2, and 3) are higher than that of the FCC state. This may suggest that the FCC structure is not the energetically favorable, and that other phases—possibly HCP or a mixed FCC + HCP configuration—might be more stable. This observation could also relate to why $\Delta\gamma_1$ is lower than $\Delta\gamma_2$ or $\Delta\gamma_3$.

Reviewer #2

(Remarks to the Author)
In this work, authors conducted in situ TEM tensile test to study the mechanical property of Fe-Mn-Co-Cr multi-principal element alloys (MPEA) and discovered the nano-scale mechanism of the MPEA’s high strength and large plasticity. The

experiments were well-designed and performed at a high quality with thorough analysis backed by theoretical calculations. The results are certainly interesting and provide important information towards understanding fundamental nanomechanics of MPEAs. However, there are several points that need clarification before the manuscript can be considered for publication in Nature Communications as listed below:

- The authors stated nano-sized crystals suffer from poor plasticity. However, this is not entirely true since there are many metals in nano-scale with high plasticity directly observed by in situ TEM experiments. For examples, https://www.nature.com/articles/ncomms2768, https://doi.org/10.1007/s40830-019-00217-6, https://pubs.acs.org/doi/10.1021/nl201890s
- Mixing enthalpy of the Fe-Mn-Co-Cr MPEA was calculated based on the elemental distributions obtained from STEM EDS. How does local strains arising from the varied atomic radius affect this?
- How was the stress measured for the stress-strain curve in Fig. 2b? It seems engineering stress was calculated using initial cross-section area, but please clarify.
- There are signs of dislocations or damages from FIB processing even at 0% strain. Since the sample thickness is relatively small (60 nm), how does this affect the strengths and ductility of the material? Samples with different thickness may be tested to confirm the effect of the surface damage on the mechanical testing results. The observed growth length of the twin boundary limited to only a few nm may be due to the sample thickness (60 nm).
- Authors claimed to have developed a novel in situ TEM device for this experiment that can simultaneously measure stress and strain. However, there have been many in situ mechanical testing holders developed for TEM, including the ones that can measure stress and strain with MEMS chips in a push-to-pull configuration. How is the device used in this work different from them?
- More details need to be explained for the in situ TEM test. What was the strain rate for the in situ tensile experiment? What is the MEMS chip made of and the geometry of the patterning? How was the force measuring beams calibrated? etc…
- How will the strain rate affect the twinning/slip behavior in the Fe-Mn-Co-Cr MPEA?
- Figures 3-5 show different defect evolution mechanisms at early – late stages during the deformation. It can help understand the process better if the strain ranges for each process is indicated or marked in the stress-strain curve.
- How is the stress-strain curve of the MD simulation compared with the in situ TEM data? Is there any agreement in the stress-strain response and the nucleation/growth of the SFs?
- In the GSFE calculation, was the stress/strain effect included? The relationships between Δγ1, Δγ2, and Δγ3 may change at different stresses.
- What interatomic potential and parameters were used for the MD simulations?
- Strain rate of the MD tensile simulation was 108 s-1. To reach 10% strain, it requires MD time in the order of miliseconds, which seems to be long for MD. Was any techniques for accelerated dynamics employed in the simulation? And at what temperature was the tensile MD performed?
- There was no twin boundary observed in the tensile simulation although nucleation and growth were observed in the in situ TEM. Please explain. Is this caused by the time/size scale effect?

Reviewer #3

(Remarks to the Author)
I co-reviewed this manuscript with one of the reviewers who provided the listed reports. This is part of the Nature Communications initiative to facilitate training in peer review and to provide appropriate recognition for Early Career Researchers who co-review manuscripts.

Reviewer #4

(Remarks to the Author)
Excellent work. The reviewer is in general supporting its publication.
Several suggestions/comments are listed below for the consideration of the authors.
1. Is the mixing entropy playing a role here at all? This has to be verified from ternary or binary alloys which also show similar negative enthalpy. Here in this work, pure metals are used as the benchmark. Fine, but it shall also include simple alloys.
2. Why has it to be a single crystal? What happens to a nanosized polycrystalline material? Will the grain orientation for the single crystal change the mechanical response?
3. Any particular reason for the choice of Fe48Mn32Co10Cr10? Is SFE or CRSS ever considered during the material selection?
4. Regarding uniform plasticity: hardening and softening are balanced all the way. The hardening part is nicely explained, but the softening part is not, which is certainly needed to account for this balance.

Version 1:

Reviewer comments:

Reviewer #2

(Remarks to the Author)
Authors conducted additional experiments and simulations and added thorough analyses and discussions, which

significantly improved the quality of the manuscript. The manuscript is recommended for publication, provided that minor English-language corrections are made. For example, in first sentence of the abstract, word "usually" is redundant since it begins with "Normally". Similar redundancies were seen in other parts of the manuscript.

Reviewer #3

(Remarks to the Author)
I co-reviewed this manuscript with one of the reviewers who provided the listed reports. This is part of the Nature Communications initiative to facilitate training in peer review and to provide appropriate recognition for Early Career Researchers who co-review manuscripts.

Reviewer #4

(Remarks to the Author)
The responses from the authors are reasonable, and the revision made to address the comments are satisfactory.

Reviewer #5

(Remarks to the Author)
This is not a full review of the manuscript, but mostly reviewing response to the first reviewer comments. I carefully reviewed the response letter and revised materials specifically regarding the issues of (1) the interpretation of necking and stress–strain behavior, and (2) the reliability of the atomistic simulations.

Regarding the discussion of necking and the stress–strain curves, I do not believe the authors have fully resolved the original concern. Necking is generally identified from the engineering stress–strain response when the slope transitions from positive to negative. Although the material in Fig. 2b exhibits limited strain hardening, necking may still occur under such conditions. However, the explanation provided by the authors is not fully convincing because the presented “true stress–strain” curves still resemble engineering stress–strain behavior. In a true stress–strain response, the stress is generally expected to continue increasing after the onset of necking due to the reduction in cross-sectional area. In contrast, Supplementary Fig. 4 still shows a decrease in stress after the proposed necking point.

In addition, the reported change in strain rate from 1.5% to 1% is relatively small and, considering the typical variability in experimental measurements, does not appear sufficient to conclusively support the authors’ interpretation. The TEM image in Fig. 2a does suggest some reduction in cross-sectional area; however, in my opinion, the evidence is still insufficient to unambiguously establish necking.

Regarding the atomistic simulations, I understand the difficulty of obtaining accurate many-body interatomic potentials for this alloy system and appreciate that the authors employed an available potential based on a Lennard-Jones-type formalism. However, the manuscript still makes strong claims that (i) the observed trends are reliable and (ii) the simulations are consistent with experiments, without providing sufficient justification for these conclusions. I remain concerned that even qualitative trends may depend strongly on the choice of interatomic potential, and this limitation is not adequately addressed.

That said, I find that the additional methodological details provided for the NEB calculations are sufficient, and the explanation for why $\Delta\gamma_1$ is lower than $\Delta\gamma_2$ or $\Delta\gamma_3$ is well justified.

Overall, while the authors have clarified some aspects of the work, I do not believe the original concerns regarding the interpretation of necking behavior and the reliability of the atomistic simulations have been fully resolved.

Version 2:

Reviewer comments:

Reviewer #5

(Remarks to the Author)
Authors' response is good and addressed all my concerns. I will recommend accepting the paper.

Open Access This Peer Review File is licensed under a Creative Commons Attribution 4.0 International License, which permits use, sharing, adaptation, distribution and reproduction in any medium or format, as long as you give appropriate credit to the original author(s) and the source, provide a link to the Creative Commons license, and indicate if changes were made.
In cases where reviewers are anonymous, credit should be given to 'Anonymous Referee' and the source.
The images or other third party material in this Peer Review File are included in the article's Creative Commons license, unless indicated otherwise in a credit line to the material. If material is not included in the article's Creative Commons license and your intended use is not permitted by statutory regulation or exceeds the permitted use, you will need to obtain permission directly from the copyright holder.
To view a copy of this license, visit https://creativecommons.org/licenses/by/4.0/

Reply to the Reviewers

We thank the reviewers for their in-depth review. The constructive suggestions provided have been considered to substantially improve the paper. A point-by-point response is included below, in which the reviewer comments (in italic font) are first repeated and then our responses follow. The changes made in the revised manuscript are indicated by black bold font.

Reviewer #1:

General comments: *This manuscript reports the results of an experimental study on the plasticity of an FeMnCoCr-based nano-sized multi-principal element alloy (MPEA), revealed through in situ mechanical testing in a transmission electron microscope (TEM). As clearly described in the Introduction, MPEAs are known to exhibit unique and desirable mechanical properties compared with conventional pure metals. However, the underlying origin of such exceptional behavior still requires further investigation.*

In this study, the authors observed that twin growth was considerably limited in the MPEA. Based on this observation, they proposed a broad distribution of stacking fault energies—arising from chemical inhomogeneity—as a primary cause. The experiments were well designed and conducted, and the overall logical flow leading to their conclusions is convincing. Nevertheless, several issues need be addressed to enhance the validity and credibility of their conclusions.

Response: We are grateful for the reviewer's immensely positive comments.

Comment 1: *On page 4, line 112, the authors report the occurrence of necking during the tensile experiments. Typically, necking is closely associated with strain hardening, yet this connection is not apparent in the experimental results. Is there a specific explanation for this discrepancy?*

Response: Thanks for the reviewer's comment. Fig. 2b in the manuscript shows the engineering stress-strain curve (stress was calculated using the initial cross-sectional area of the specimen). Based on our true stress-strain and strain-hardening rate curves (Supplementary Fig.4), the strain-hardening rate of NSC remains above ~ 1.5 over a large range of strain, resulting in a relatively large uniform elongation. Once the strain exceeds ~17%, the value of the strain hardening rate drops below 1.0, and necking occurs. This is consistent with previous classical theory. In response to the reviewer's comments, we have included the corresponding true stress-strain curve and normalized strain-hardening rate in the supplementary materials (Supplementary Fig.4) and revised the manuscript accordingly. The revised text reads:

"Based on the true stress-strain curves and the normalized strain-hardening rate curve (Supplementary Fig. 4), NSC exhibits strong strain-hardening at the early stage. As tensile strain increases, the strain-hardening rate remains at ~ 1.5 over a considerable range (5 ~ 17%), which produces the relatively large uniform elongation. When the strain exceeds ~17%, the strain-hardening rate drops below 1.0. At this point, regions with low barriers for dislocation nucleation have been exhausted, and further strain is accommodated by dislocation nucleation in sites with much higher energy barriers. Consequently, NSC can no longer disperse stress through uniform

plastic deformation and instead undergoes non-uniform deformation in weaker regions, rapidly leading to stress concentration and necking."

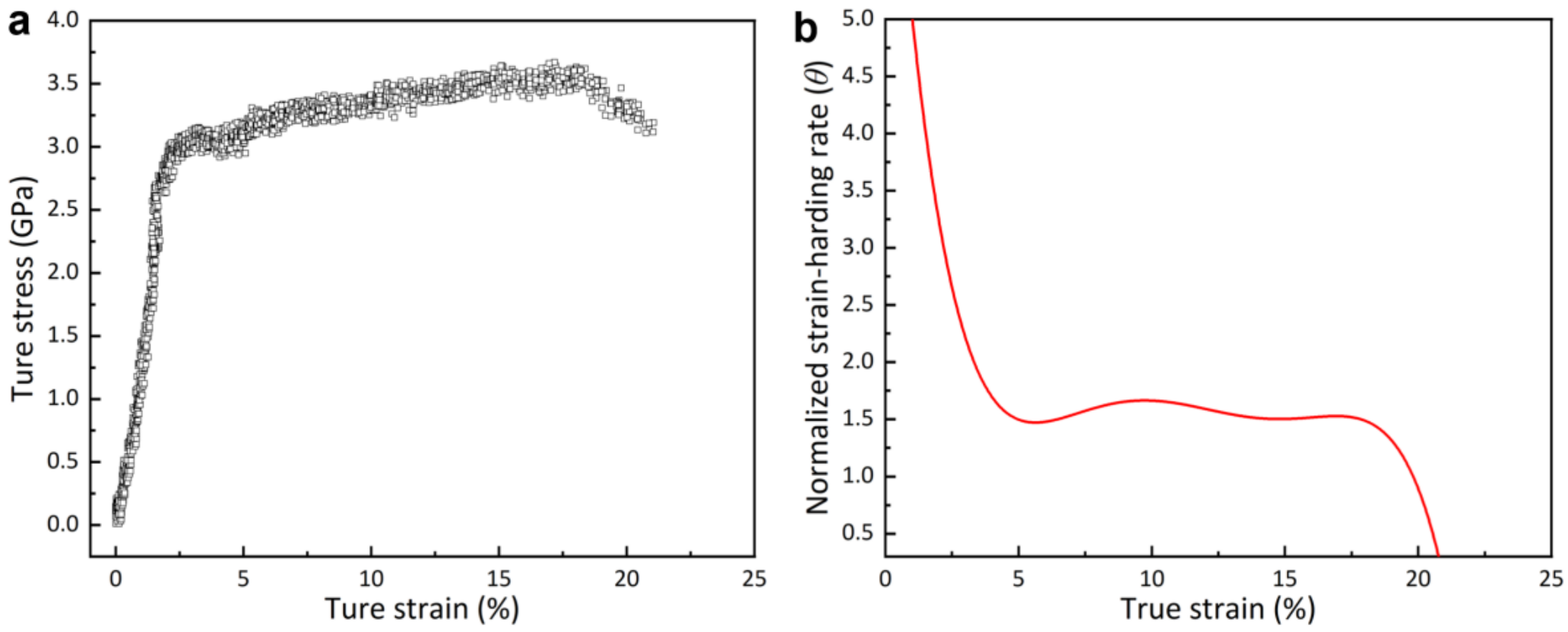

Fig. 4 | (a) True stress-strain curve of the NSC; (b) Normalized strain-hardening rate curve of the NSC.

Comment 2: *In the paragraph beginning on page 5, line 156, the authors state that the intersection of full and partial dislocations with twin boundaries (TBs) is a critical deformation mode. However, describing this simply as an "intersection of dislocations with TBs" appears to refer to a resultant phenomenon rather than a deformation mechanism. Do the dislocations form within the TBs and subsequently glide toward the surface or adjacent TBs, or are they nucleated at the free surface and then glide into and become absorbed by the TBs?*

Response: Thanks for the reviewer's constructive comment. Indeed, our initial phrasing about Fig.5 was overly simplistic. Based on our in-situ atomic-scale observations, both dislocation-TB interaction and partial-full dislocation interaction were observed. First, full dislocations nucleate from the free surface or CTB-surface intersection; many of these dislocations glide on {111} planes that intersect with CTBs and are blocked by the TB. Second, partial dislocations nucleate from the free surface, glide toward CTBs, and intersect with them, leaving SFs that terminate at the TB. Third, partial dislocations nucleate from the CTB-surface intersection, glide along the TB, causing step formation on the TB and TB migration. In response to the reviewer's comments, we have thoroughly revised Fig. 5 and provided details on the deformation mechanism. The details are as follows:

"Fig. 5 presents a typical example of in situ HRTEM images that were captured at a strain of ~15%, where both dislocation-TB interaction and partial-full dislocation interaction were observed. As shown in Fig. 5a, many full dislocations on {111} planes are blocked by the TB (marked with "T"). By comparing the positions of full dislocations D1 in Figs. 5a-b, it seems that this dislocation nucleates from the free surface intersection, and moves from the right side toward the CTB and is blocked by the CTB. As shown in Figs. 5b-c, full dislocations D4 and D5 also nucleate from the free surface; they move from the right side toward the CTB and are then blocked by the CTB. While comparing the positions of full dislocations D2 and D3 in Figs. 5b-c, it can be seen that these two dislocations are

located near the CTB, and move from the CTB toward the right side, demonstrating that these full dislocations nucleate from the CTB-surface intersection. Besides full dislocation, many partial dislocations nucleate and intersect with CTBs, leaving SFs that terminate at the CTB, as shown in Figs. 5a-b (labeled "SF1-SF3"). The arrows indicate that these SFs move from the right side toward the CTB, increasing and becoming progressively closer to the CTB, demonstrating that these partial dislocations nucleate from the free surface. As shown in Figs. 5b-c, the newly formed SF4 and SF5 underwent the similar process as SF1-SF3. In addition, both the steps on the CTB and the width of the twin have changed (Figs. 5a-c), which results from partial dislocations nucleating from the CTB-surface intersection and gliding along the CTB, which is similar to what is show in Fig. 3."

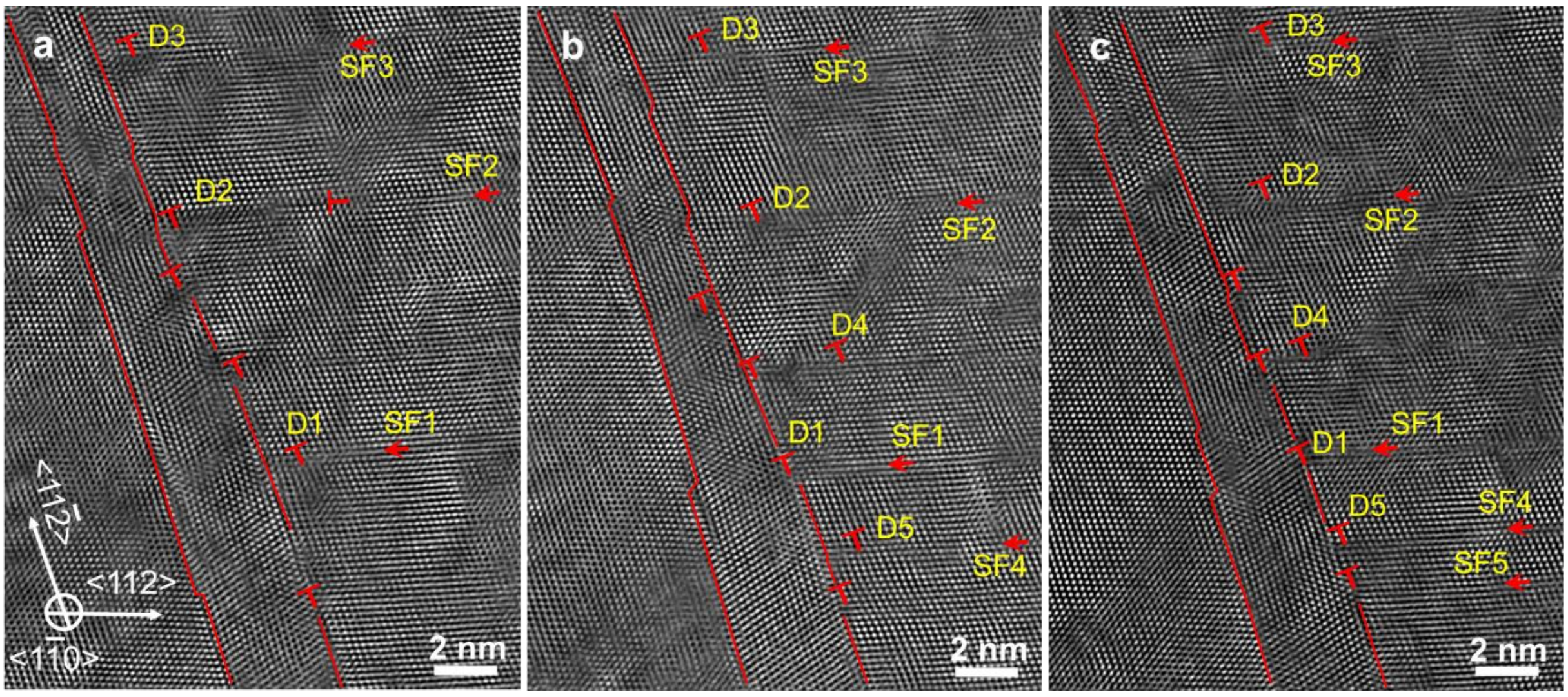

Fig. 5 | HRTEM images illustrating the interaction between partial dislocations and TBs during tensile straining of the NSC. (a) to **(c)** A twin is present on the left side, with the TBs marked by red lines. On the right side of the twin, numerous full dislocations are visible, denoted by "T", along with several SFs, indicated by red arrows. As loading progresses, randomly emitted partial dislocations accumulate at the TB, forming full dislocations and contributing to strain hardening.

Comment 3: *The TEM bright-field images presented in Figures 2e-h do not convincingly serve as experimental evidence supporting the authors' claims. The image quality appears too low to clearly resolve the relevant features, such as dislocations, making it difficult for this reviewer to interpret the microstructural details.*

Response: Thanks for the reviewer's comment. In response to the reviewer's comments, we have provided new results showing the twinning process (shown in Fig. 2e-f and movie 1) and revised the manuscript accordingly. The details are as follows:

"Fig. 2e-g show magnified TEM images illustrating the early-stage deformation. Nanotwins are indicated by red arrows. During the in-situ process, the number of fine nanotwins progressively increases with applied strain, without significant twin thickening (also see Supplementary Movie 1).

Fig. 2h presents the HAADF image of the deformed specimen, confirming the widespread presence of nanotwins."

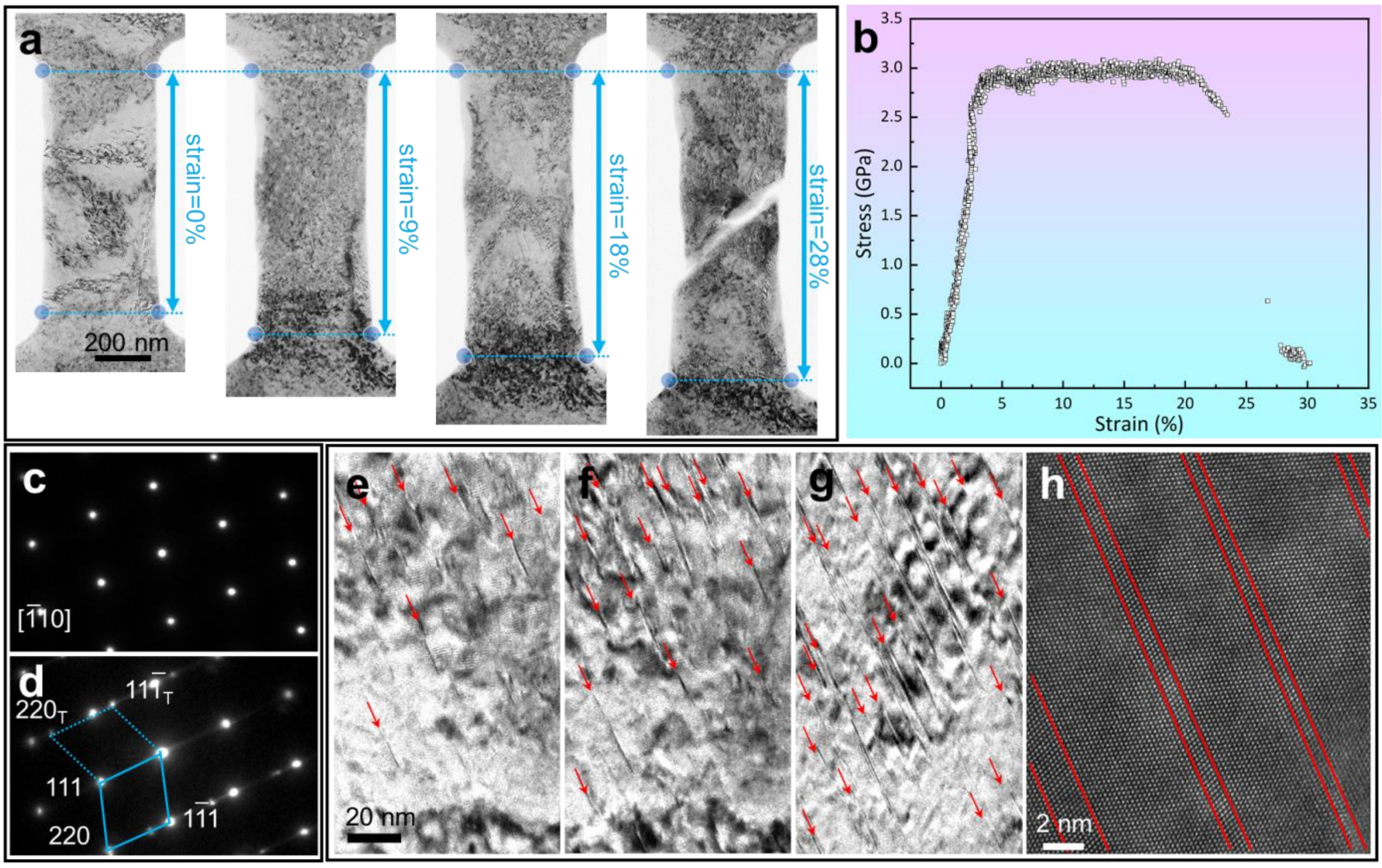

Fig. 2 | In-situ observation of the tensile process. (**a**) Bright-field TEM images captured during in-situ tensile straining at low magnification. (**b**) Tensile stress-strain curve of the NSC. (**c**) and (**d**) SAED patterns before and after deformation, illustrating the formation of SFs and nanotwins. (**e**) to (**g**) Magnified TEM bright-field images of the deformation in the early stage, showing the propagation of SFs and nanotwins. (**h**) HAADF image of the deformed specimen.

Comment 4: *The reliability of the atomistic simulation data shown in Figure 6b requires further verification. While the authors primarily discuss the energy barriers in the coordinate-energy plot, it should be noted that the energies of the metastable configurations (i.e., those corresponding to normalized displacements 1, 2, and 3) are higher than that of the FCC state. This may suggest that the FCC structure is not the energetically favorable, and that other phases—possibly HCP or a mixed FCC + HCP configuration—might be more stable. This observation could also relate to why* $\Delta\gamma_1$ *is lower than* $\Delta\gamma_2$ *or* $\Delta\gamma_3$.

Response: Thanks for the reviewer's valuable comment. In our initial simulations, we employed the second-nearest-neighbor modified embedded-atom method (2NN MEAM) potential developed by Choi [doi.org/10.1016/j.commatsci.2017.01.002]. This potential was parameterized based on binary systems such as Co-Cr and Co-Fe and extended to multi-component alloys like CoCrFeMnNi. However, as you pointed out, the 2NN MEAM potential is robust for binary systems; it may not fully capture the complex characteristics of MPEAs. Thus, we have recalculated the GSFE by using an effective potential developed by Gröger [doi.org/10.1088/1361-651X/ab7f8b], which is specifically designed for fcc CoCrFeMnNi alloys.

This potential employs a Lennard-Jones-type formalism with parameters derived from pure element properties and mixing rules for regular solutions, making it more suitable for capturing the properties of the MPEA. This change was motivated by the fact that the Cauchy pressure (C12-C44) in CoCrFeMnNi is small, indicating that pair interactions dominate the bonding—a feature better represented by a simple pair potential than by many-body methods like 2NN MEAM. It should be emphasized that the actual potential of MPEAs is extremely difficult to determine; thus, the actual values of GSFE are highly dependent on the potential's refinement. Thus, our calculated values represent only a trend, and the results are consistent with our experiments. In response to the reviewer's constructive comments, we have updated Fig. 6, revised the corresponding text, and added a short discussion about the use of MD SFE values, and have also made corresponding revisions to the Methods section. The details are as follows:

"The values of $\Delta\gamma_2$ and $\Delta\gamma_3$ are slightly smaller than $\Delta\gamma_1$, and their distributions exhibit significant overlap. This is in stark contrast to the typical relationship observed in pure metals and conventional alloys ($\Delta\gamma_1 \square \Delta\gamma_2 = \Delta\gamma_3$)[38, 39]. In pure metals, partial dislocations tend to emit continuously, which favors the formation of thick twins over the generation of new SFs. This is because the energy barrier for nucleating subsequent partial dislocations on crystallographic planes adjacent to the first partial (resulting in twin formation) is substantially lower than that for nucleation on distant planes (which leads to SFs)."

"It should be noted that the actual values of GSFE are highly dependent on the potential's refinement. Because the actual potential of MPEAs is extremely difficult to determine, our calculated values may represent only a trend, and the results are consistent with our experiments. Though the values of GSFE may vary when using different potentials, they may exhibit a similar trend, in which the GSFE ($\Delta\gamma_1$, $\Delta\gamma_2$ and $\Delta\gamma_3$) exhibit significant overlap."

"Simulation of generalized stacking fault energy. The GSFE curves for multiple alloy compositions were calculated to assess their intrinsic deformation propensity. The atomic interactions in the quaternary $Fe_{48}Mn_{32}Co_{10}Cr_{10}$ alloy were modeled using the potential developed by Gröger [70]. A total of 250 independent simulation cells were constructed for each composition to ensure statistical significance, as compositional fluctuations in these multi-component systems can significantly influence the GSFE [46]. Each cell contained a planar fault area of approximately 4.2 × 5.4 nm within the FCC lattice. To determine the GSFE pathways, three characteristic planar faults were created from the perfect FCC structure: a one-layer ISF, a two-layer ESF, and a three-layer twin. These defects were generated by introducing Shockley partial dislocations with Burgers vectors of b = 1/6<112> on adjacent {111} close-packed planes. The energy landscape along the transformation pathways (i.e., from perfect FCC to ISF, ISF to ESF, and ESF to twin) was calculated using the Nudged Elastic Band (NEB) method performed in LAMMPS. For each pathway, ten intermediate images were uniformly interpolated between the corresponding initial and final states. The NEB

calculation was stopped when the change in energy of the minimization iterations was less than 1 × 10^{-14} eV. To investigate the effect of pre-existing lattice strain on the deformation energy landscape, the GSFE for the $Fe_{48}Mn_{32}Co_{10}Cr_{10}$ alloy was also calculated under applied tensile strains."

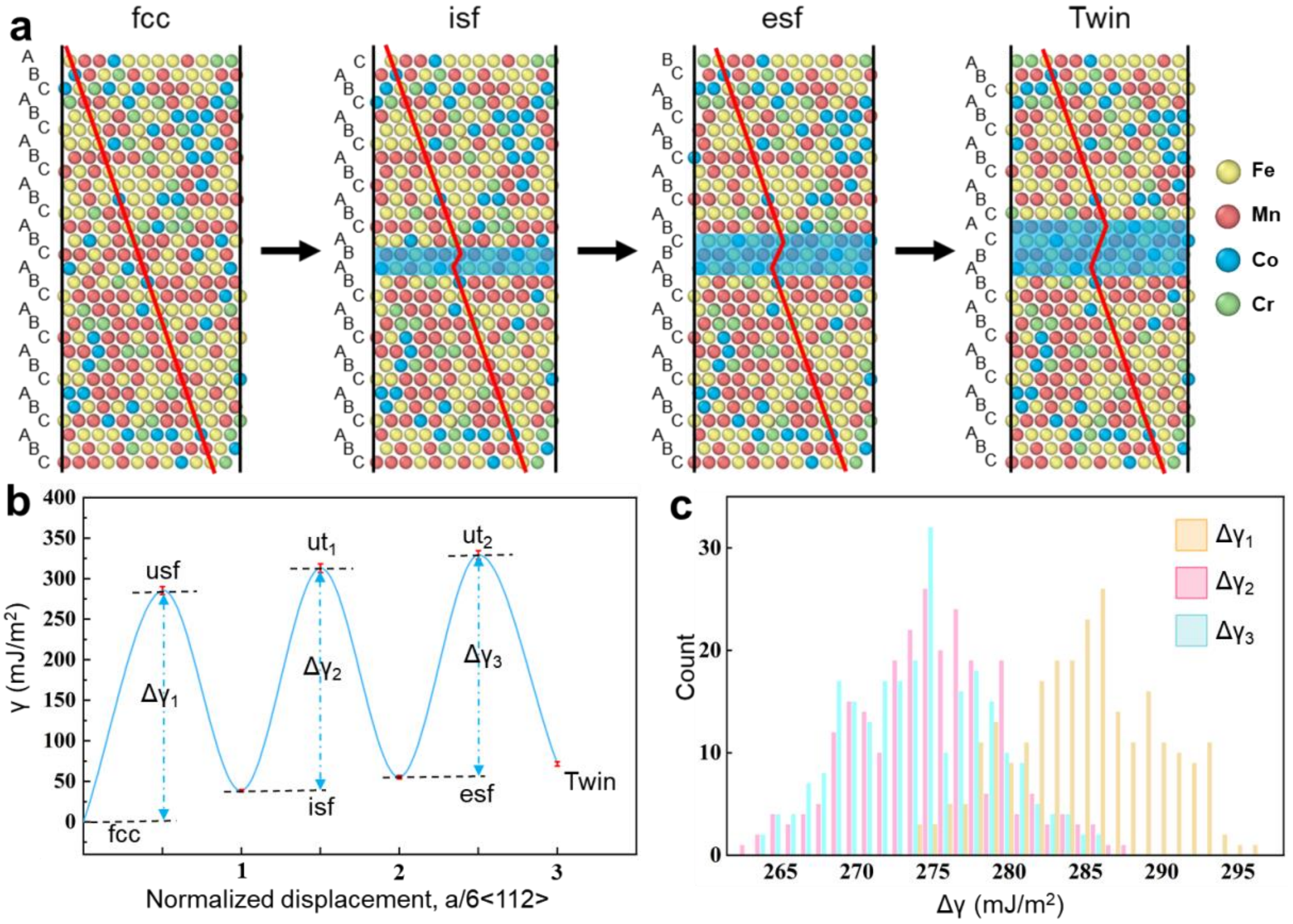

Fig. 6 | The simulation of GSFE in NSC. (a) Schematic of the twin formation pathway. The formations of a one-layer ISF, two-layer ESF, and three-layer twins are created by introducing Shockley partials with ***b***=1/6<112> on the adjacent {111} plane. (b) GSFE curve of the NSC along the twin formation pathway. (c) Distribution of unstable SFE barriers for three selected paths: FCC to ISF, ISF to ESF, and ESF to twin, corresponding to $\Delta\gamma_1$, $\Delta\gamma_2$, and $\Delta\gamma_3$ in (b), respectively.

Reviewer #2:

General comments: *In this work, authors conducted in situ TEM tensile test to study the mechanical property of Fe-Mn-Co-Cr multi-principal element alloys (MPEA) and discovered the nano-scale mechanism of the MPEA's high strength and large plasticity. The experiments were well-designed and performed at a high quality with thorough analysis backed by theoretical calculations. The results are certainly interesting and provide important information towards understanding fundamental nanomechanics of MPEAs. However, there are several points that need clarification before the manuscript can be considered for publication in Nature Communications as listed below:*

Response: We are grateful for the reviewer's immensely positive comments.

Comment 1: *The authors stated nano-sized crystals suffer from poor plasticity. However, this is not entirely true since there are many metals in nano-scale with high plasticity directly observed by in situ TEM*

experiments. For examples, https://www.nature.com/articles/ncomms2768, https://doi.org/10.1007/s40830-019-00217-6, https://pubs.acs.org/doi/10.1021/nl201890s

Response: Thank the reviewer for drawing our attention to these interesting papers. We have carefully read these papers and revised the introduction and discussion accordingly. We have also conducted literature research carefully. We notice that, in most cases, nanosized metals usually exhibit limited dislocation nucleation or localized deformation [60-62], leading them to usually exhibit ultrahigh strength but poor plasticity. Indeed, there are also many cases where nanoscale metals exhibit large strength and high ductility. Such as in previous studies on nanoscale Cu [63] and NiTi [64], dislocations or phase transformations are randomly nucleated on multiple slip systems or positions, therefore leading to the random distribution of dislocations and deformation twins or martensite. This deformation mode is similar to our in-situ observation, which can effectively alleviate deformation localization, thereby enabling nanosized metals to exhibit both ultrahigh strength and large uniform elongation. In response to the reviewer's constructive comments, we have revised the abstract, introduction and discussion accordingly, the details as follows:

"Normally, many nanosized metals with limited dislocation nucleation and dislocation multiplication usually exhibit poor plasticity."

"Previous experiments and simulations have shown that many nanosized metals exhibit high strengths but usually suffer from low plasticity."

"Actually, nanosized metals do not always exhibit poor ductility. Based on previous classic experiments and theoretical studies, in most cases, nanosized metals readily exist in a low dislocation density state, require high stress for the nucleation of new dislocations, and exhibit limited dislocation multiplication during deformation. These features make nanosized metals prone to localized deformation and early necking, thereby leading them to usually exhibit ultrahigh strength but poor plasticity [60-62]. However, there are many cases where nanoscale metals can also exhibit large strength and high ductility. Previous studies show that the random nucleation of dislocations leads to large uniform elongation in nanoscale Cu [63]. Large uniform elongation that resulted from random generation of martensitic transformation was also observed in nanoscale polycrystalline NiTi [64]. These deformation modes are similar to those observed in the current study, which can effectively alleviate deformation localization, thereby enabling nanoscale metals to exhibit both ultrahigh strength and large uniform elongation. Our current results, combined with previous classical studies, suggest that equipping nanoscale metals with the ability to randomly nucleate new dislocations or deformation twins/phases is an efficient method to overcome the strength-ductility trade-off in nanoscale metals."

Comment 2: *Mixing enthalpy of the Fe-Mn-Co-Cr MPEA was calculated based on the elemental distributions obtained from STEM EDS. How does local strains arising from the varied atomic radius affect this?*

Response: Thanks for the reviewer's comment. In the Fe-Mn-Co-Cr system, the constituent elements are all adjacent first-row transition metals with very similar atomic radii. Specifically, the radii are Fe (127 pm), Mn (132 pm), Co (126 pm), and Cr (127 pm). This small difference results in a very low lattice strain energy [31, 32]. In response to the reviewer's comments, several sentences were added in the revised manuscript, as follows:

"Because Fe, Mn, Co, and Cr are adjacent first-row transition metals with very similar metallic radii (Fe: 127 pm, Mn: 132 pm, Co: 126 pm, Cr: 127 pm), the effect of elastic strain (due to atomic size mismatch) on the enthalpy calculation can be neglected [31, 32]."

Comment 3: *How was the stress measured for the stress-strain curve in Fig. 2b? It seems engineering stress was calculated using initial cross-section area, but please clarify.*

Response: Thanks for the reviewer's comment. Fig. 2b shows the engineering stress-strain curve, which was calculated using the initial cross-sectional area. We have added the details of stress-strain curve measurement in the Supplementary information, as follows:

"Stress-strain measurement. As shown in Supplementary Fig. 1, the MEMS chip integrates two capacitive displacement sensors, namely Sensor A and Sensor B. These two sensors, capable of measuring the axial displacement at the front and rear ends of the tested specimen, were integrated at both sides of the gauge section. This setup enables real-time acquisition of the strain and stress of the specimen during tensile loading. The lengths of the sample before and after tensile are denoted as l_1 and l_2, respectively. During tensile deformation, the displacements measured by Sensor A and Sensor B are x_a and x_b, respectively. Accordingly, the engineering strain ε of the specimen can be calculated using Eq. (1):

$$\varepsilon = \frac{l_2 - l_1}{l_1} = \frac{x_a - x_b}{l_1} \quad \textbf{(1)}$$

At the rear side of Sensor B, a double-ended fixed beam with stiffness k was integrated. During tensile loading, the beam undergoes flexural deformation, with a central deflection of x_b. The force applied to the beam, F_b, can be calculated according to Hooke's law as follows:

$$F_b = kx_b \quad \textbf{(2)}$$

Because the central load on the doubly clamped beam, F_b, and the tensile load experienced by the specimen, F, constitute an action-reaction pair, they are equal in magnitude according to Newton's third law (i.e., $F = F_b$). After determining the specimen's initial width w_s and initial thickness d_s from electron microscopy measurements, the engineering stress during tensile loading can be obtained using Eq. (3):

$$\sigma = \frac{F_s}{w_s d_s} = \frac{kx_b}{w_s d_s} \quad \textbf{(3)}$$

It should be noted that, because Eqs. (1) and (3) are formulated using the specimen's initial dimensions; the stress and strain obtained in this work correspond to engineering stress and engineering strain, respectively.

The length *l*, width *w*, and thickness *d* of the doubly clamped beam can be precisely defined by semiconductor microfabrication (etching) processes; therefore, the beam stiffness *k* can be calculated using Eq. (4):

$$k = \frac{2Ew^3d}{l^3} \quad \textbf{(4)}$$

where *E* denotes the axial Young's modulus of the clamped beam."

[Figure Redacted]

Fig. 1 | (a) In-situ tensile mechanical property quantification TEM sample holder. (b) Three-dimensional simulation of the double-tilt angles. (c) Image of the integrated MEMS tensile device. (d) Schematic illustration of the quantitative in situ mechanical MEMS chip.

Comment 4: *There are signs of dislocations or damages from FIB processing even at 0% strain. Since the sample thickness is relatively small (60 nm), how does this affect the strengths and ductility of the material? Samples with different thickness may be tested to confirm the effect of the surface damage on the mechanical*

testing results. The observed growth length of the twin boundary limited to only a few nm may be due to the sample thickness (60 nm).

Response: Thank you for this constructive comment. Following your suggestion, we have conducted in-situ mechanical experiments on NSCs with thicknesses of 230 nm and 160 nm, including corresponding true stress-strain curves (Supplementary Figs. 5 and 6). We notice that as sample thickness increases, NSCs' strength decreases while elongation increases. This size effect originates from the decrease in size, which may constrain dislocation motion (requiring higher stress to activate dislocation sources) and reduce intragranular dislocation storage capacity, thereby increasing strength but decreasing ductility. Though the TEM imaging quality is relatively poor (due to the large thickness), it clearly shows abundant SFs and nanotwins in the thicker NSCs, similar to that observed in 60 nm thickness NSCs. This indicates the observed deformation mode should not be an artifact of FIB damage, but rather the intrinsic geometric constraints on twin propagation at nanoscale dimensions. The results of large thickness NSCs were added in the Supplementary materials, and a short discussion was added in the revised manuscript, as follows:

"We also conducted tests on NSCs with thicknesses of 160 nm and 230 nm (Supplementary Figs. 5 and 6). The in-situ observations show that there are also abundant SFs and nanotwins, indicating the observed deformation mode should not be an artifact of FIB damage. The true strain-stress curve shows a trend that, as the thickness of NSCs increases, their strength decreases while elongation increases. This size effect is consistent with previous studies [38]."

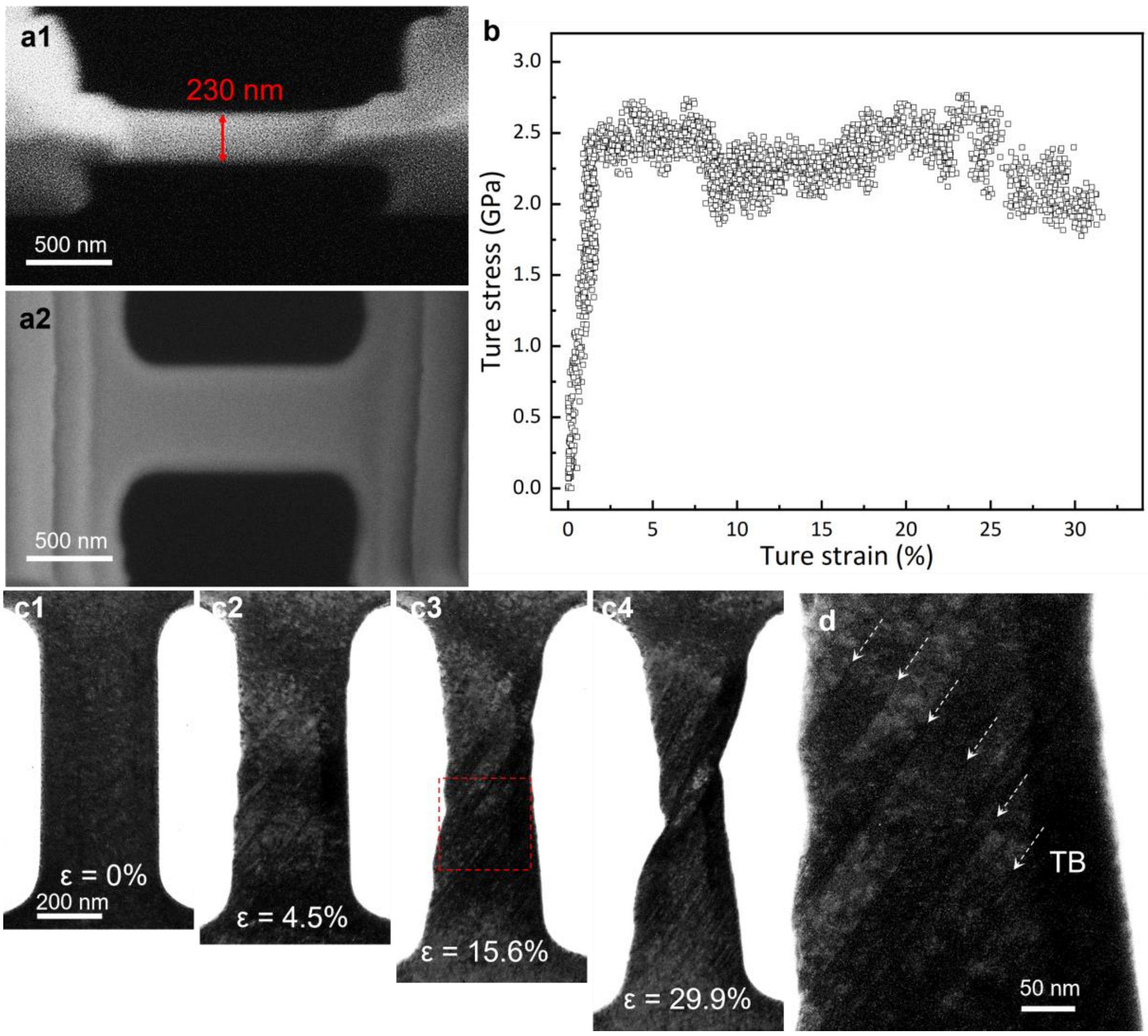

Fig. 17 | In-situ TEM experiment of NSC with a thickness of 230 nm. (a) Front and side views of the NSC sample in SEM. (b) True stress-strain curve. (c) Bright-field images during in-situ tensile testing. (d) Enlarged image of the area outlined in red in **c**.

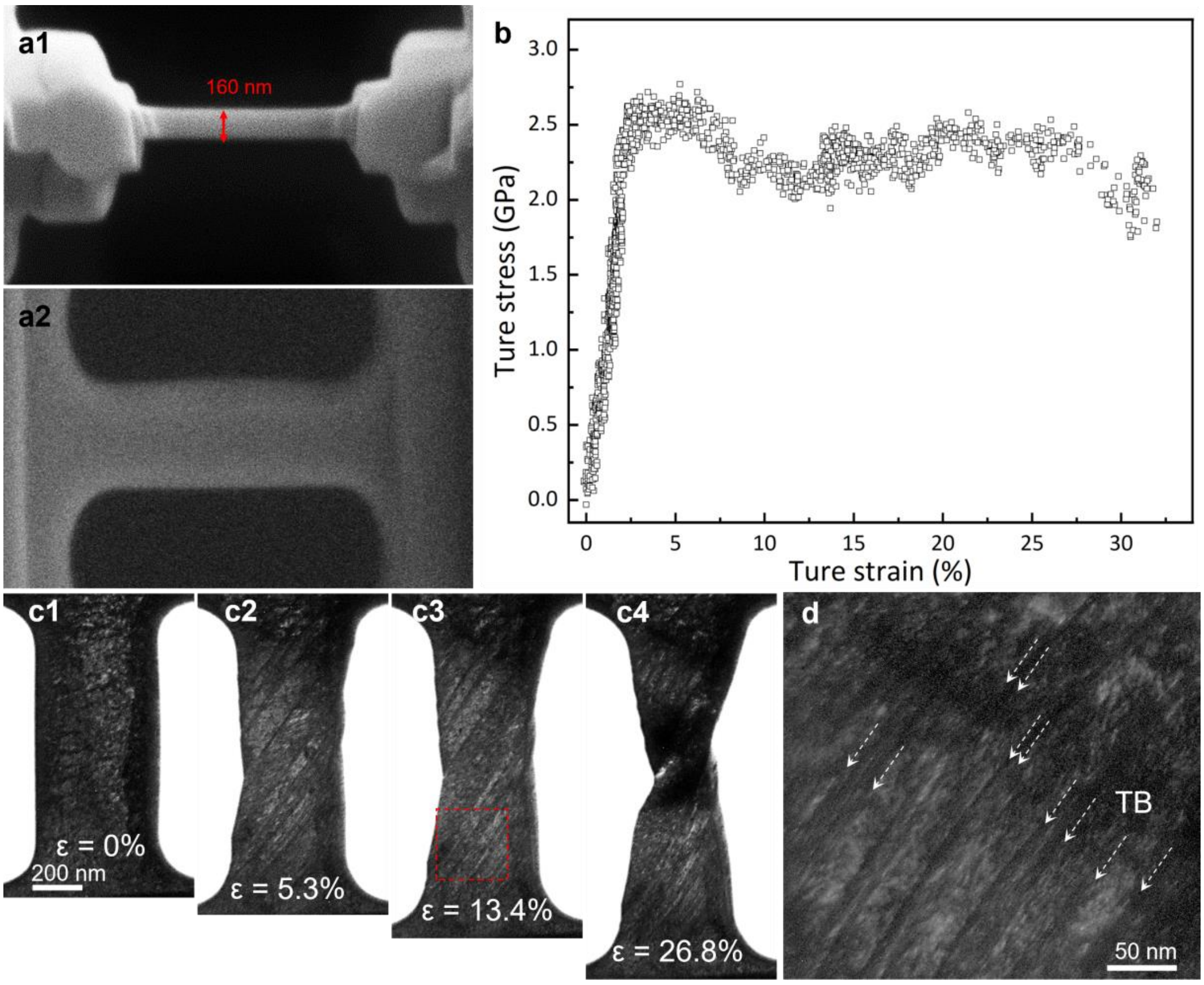

Fig. 18 | In-situ TEM experiment of NSC with a thickness of 160 nm. (a) Front and side views of the NSC sample in SEM. (b) True stress-strain curve. (c) Bright-field images during in-situ tensile testing. (d) Enlarged image of the area outlined in red in **c**.

Comment 5: *Authors claimed to have developed a novel in situ TEM device for this experiment that can simultaneously measure stress and strain. However, there have been many in situ mechanical testing holders developed for TEM, including the ones that can measure stress and strain with MEMS chips in a push-to-pull configuration. How is the device used in this work different from them?*

Response: Thanks for the reviewer's comment. Our newly developed in-situ TEM device enables stress-strain measurement and simultaneous acquisition of atomic-scale HRTEM images during deformation. The details of the device were added in the method section, and we also revised the manuscript accordingly. Details are as follows:

"Quantitative in-situ tensile TEM system. The newly developed double-tilt in situ TEM device comprises an integrated capacitance signal processing module, actuator, MEMS chip and specimen holder, as shown in Supplementary Fig. 1. The integrated capacitance signal processing module can acquire and record the strain-stress in real time with high stability and precision. The actuator provides an external force on the sample. The MEMS chip developed in this work integrates two

capacitive displacement sensors on a single chip platform, which enables the acquisition of stress-strain data (see details in stress-strain measurement section below). As shown in Supplementary Fig. 1b, the developed specimen holder in this work has double tilt ability (±20° about both the α-axis and β-axis), allowing the electron beam to be accurately aligned along low-index zone axes of the specimen and thus enabling atomic-resolution imaging. This capability is key for in situ observation of the deformation process at the atomic-scale, and simultaneously acquiring stress-strain data. Owing to constraints such as actuator size and the limited pole-piece gap in TEM, previously designed specimen holders were only with single tilt ability, making it difficult to obtain atomic-scale imaging. The dual-sensor integrated MEMS chip and double tilt ability developed in the current study allow synchronized atomic-scale observation and quantitative strain-stress readout."

[Figure Redacted]

Fig. 1 | (a) In-situ tensile mechanical property quantification TEM sample holder. (b) Three-dimensional simulation of the double-tilt angles. (c) Image of the integrated MEMS tensile device. (d) Schematic illustration of the quantitative in situ mechanical MEMS chip.

Comment 6: *More details need to be explained for the in situ TEM test. What was the strain rate for the in situ tensile experiment? What is the MEMS chip made of and the geometry of the patterning? How was the force measuring beams calibrated? etc…*

Response: We appreciate the reviewer's suggestion. We have provided the structures of the MEMS chip in supplementary materials and revised the manuscript accordingly. Details are as follows:

"The structure of the MEMS chip. As shown in Supplementary Fig. 1a, the MEMS chip adopts a semi-open C-shaped architecture, with the specimen region positioned between the two displacement sensors. Each sensor employs a parallel-plate capacitive configuration (Supplementary Fig. 1b). During tensile loading, the mass block drives the movable plate to move, thereby modulating the gap between the movable and fixed electrodes. The onboard signal-processing module within the holder performs amplification, demodulation, and acquisition of the capacitance signal (Supplementary Fig. 1c), enabling real-time strain-stress readout via an integrated capacitance signal processing module. With this method, the strain rate is ~10^{-4} s^{-1}.

As shown in Supplementary Fig. 2c, the MEMS chip was fabricated from a sandwich-structured silicon-on-insulator wafer consisting of a Si basal layer, a buried SiO_2 layer, and a p-type device layer (resistivity 1-10 Ω·cm). The buried oxide electrically isolates the device layer from the basal layer, ensuring stable and reliable electrical readout. Microfabrication began with dry etching to define the differential capacitive displacement sensors, force-sensing beam, interconnects, and other microstructural units in the device layer, with the load beam axis aligned along the <110> crystallographic direction. The basal layer and buried oxide were then removed by wet etching to release the suspended structures. To reduce interconnect resistance and improve signal transmission, a thin Au film was subsequently deposited on the interconnects by sputtering, enhancing conductivity and electrical contact stability. Then, the MEMS chip was assembled onto the integrated stage shown in Supplementary Fig. 1b.

The capacitance-to-displacement calibration was performed inside the TEM using the digital double-tilt in situ holder. During actuator-driven motion of the proof mass, the relative displacement of the movable electrode with respect to the fixed electrode was directly measured from TEM images, while the corresponding capacitance output was recorded synchronously by the signal-processing module. Establishing a one-to-one correspondence between displacement and capacitance yields the capacitance-displacement calibration curve (Supplementary Fig. 2d). In subsequent in situ mechanical experiments, the real-time capacitance signals can be readily converted into displacement using this calibration, enabling quantitative determination and analysis of mechanical responses. "The platform's accuracy was validated by testing a FIB-prepared <002> single-crystal Cu standard sample, as shown in Supplementary Fig. 3. The measured Cu <002> Young's modulus from the stress-strain curve was 67.718 GPa, which deviates by only 1.8% from the first-principles value of 66.6 GPa

^{1}, with the minor error attributed to the FIB-induced surface amorphous layer and sample geometry uncertainties.”

[Figure Redacted]

Fig. 2 | (a, b) Side view and top view photographs of the in-situ tensile device. (c) Image of the quantitative in situ mechanical MEMS device. (d) Enlarged view of the capacitive sensor. (e) Exploded view of the mechanically quantitative MEMS chip. (f) Calibration curve relating the displacement of the movable electrode to the capacitance variation.

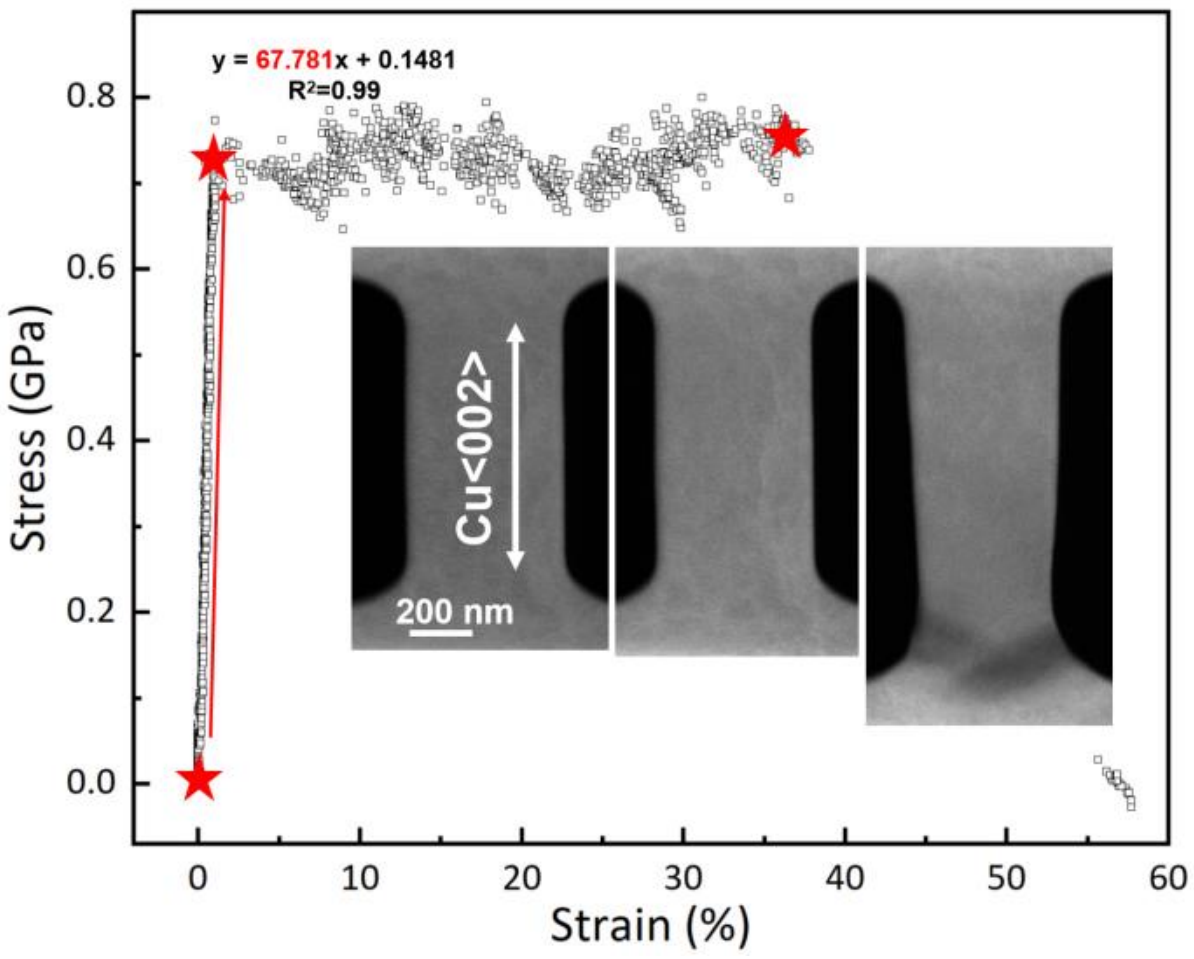

Fig. 3 | Tensile stress-strain curve of single crystal Cu.

Comment 7: *How will the strain rate affect the twinning/slip behavior in the Fe-Mn-Co-Cr MPEA?*

Response: Thanks for the reviewer’s comment. In our study, the strain rate is in the range of ~ 10^{-4}~10^{-3} s^{-1}, which is within conventional testing regimes for alloys. This conventional strain rate should have a negligible effect on deformation twins, as previous studies have shown that only extremely high strain rates (e.g., >10^{2} s^{-1}) can promote twinning/SF activity [65, 66]. Thus, in our experiments, deformation twins and SFs are intrinsic deformation mechanisms in the NSC. In response to the reviewer’s comments, several sentences were added in the revised manuscript, as follows:

“Here, it should be noted that there are many factors that can affect the mechanical behavior of

metals. First, the strain rate can significantly affect the uniform elongation and deformation mechanism of metals. In current experiments, the strain rate in the current experiments is in the range of 10^{-4} to 10^{-3} s^{-1}. Such conventional strain rates should have a negligible effect on deformation twins, as only extremely high strain rates (e.g., >10^2 s^{-1}) can promote twinning/SF activity [65, 66]. Thus, the deformation twins and SFs observed in our experiments are intrinsic deformation mechanisms in the NSC rather than effects of strain rate."

Comment 8: *Figures 3-5 show different defect evolution mechanisms at early - late stages during the deformation. It can help understand the process better if the strain ranges for each process is indicated or marked in the stress-strain curve.*

Response: Following the reviewer's constructive suggestion, we have revised the manuscript accordingly. These descriptions have been added to the relevant results and discussion sections, providing a clearer link between the mechanical response and the underlying microstructural processes. The details are as follows:

"Fig. 3 presents in-situ atomic-scale evidence that the plastic deformation of the NSC is mediated by the random generation of deformation twins and partial dislocations from the onset of plastic deformation up to ~5% strain."

"Fig. 4 presents the HRTEM images taken from the ~5% to ~15% strain stage, showing full and partial dislocations on two intersecting {111} planes."

"Fig. 5 presents a typical example of in situ HRTEM images that were captured at a strain of ~15%, where both dislocation-TB interaction and partial-full dislocation interaction were observed."

Comment 9: *How is the stress-strain curve of the MD simulation compared with the in situ TEM data? Is there any agreement in the stress-strain response and the nucleation/growth of the SFs?*

Response: Thanks for the reviewer's comment. We have supplemented the MD simulation stress-strain curves in Supplementary Fig. 9. These higher stresses in MD simulations are attributed to the high strain rate, i.e., MD simulations with a higher strain rate by approximately 11 orders of magnitude than the experiments. Furthermore, there inevitably exist defects in experimental samples. These pre-existing defects can also lead to the measured strength of NSCs deviating from the MD simulations. Nevertheless, the deformation mechanisms and the overall trend of stress-strain fluctuations of NSCs remain consistent. In response to the reviewer's comments, several sentences were added in the revised manuscript, as follows:

"The MD simulation also shows similar deformation mechanisms that are consistent with our experiments (Supplementary Fig. 8). The simulations thus indeed provide guidance for understanding the deformation mechanisms. The higher stresses in MD simulations are attributed to a higher strain rate by approximately 11 orders of magnitude than the experiments (Supplementary Fig. 9). Furthermore, some defects undoubtedly existed in the experimental samples, which can also lead to the measured strength of NSCs deviation from the MD simulations."

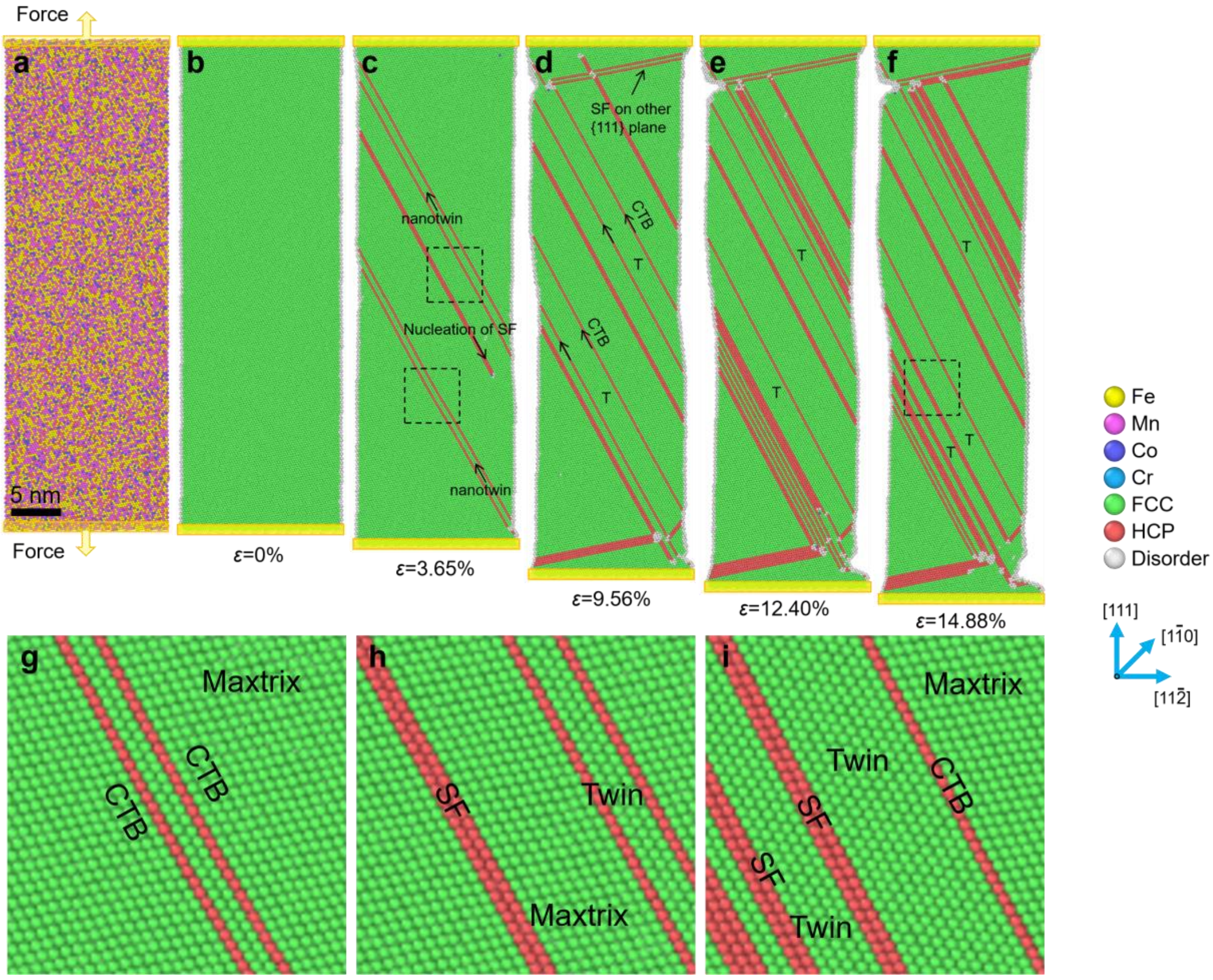

Fig. 8 | The molecular dynamics simulation of $Fe_{48}Mn_{32}Co_{10}Cr_{10}$ MPEA during in-situ tensile deformation. (a) After model relaxation, the distribution of the Fe, Mn, Co, and Co elements. (b) to (f) The tensile process from 0% to 14.88% strain is displayed. (g) to (i) Magnified details of SF and CTB. Green atoms represent the face-centered cubic structure. Red atoms represent the hexagonal close-packed structure, which can be considered as SFs formed by the emission of dislocations. During deformation, dislocations are emitted on different {111} planes, leading to the microstructure of non-adjacent SFs. (g) to (f) Enlarged image of the area outlined in red in c to e, revealing details of CTB and SF.

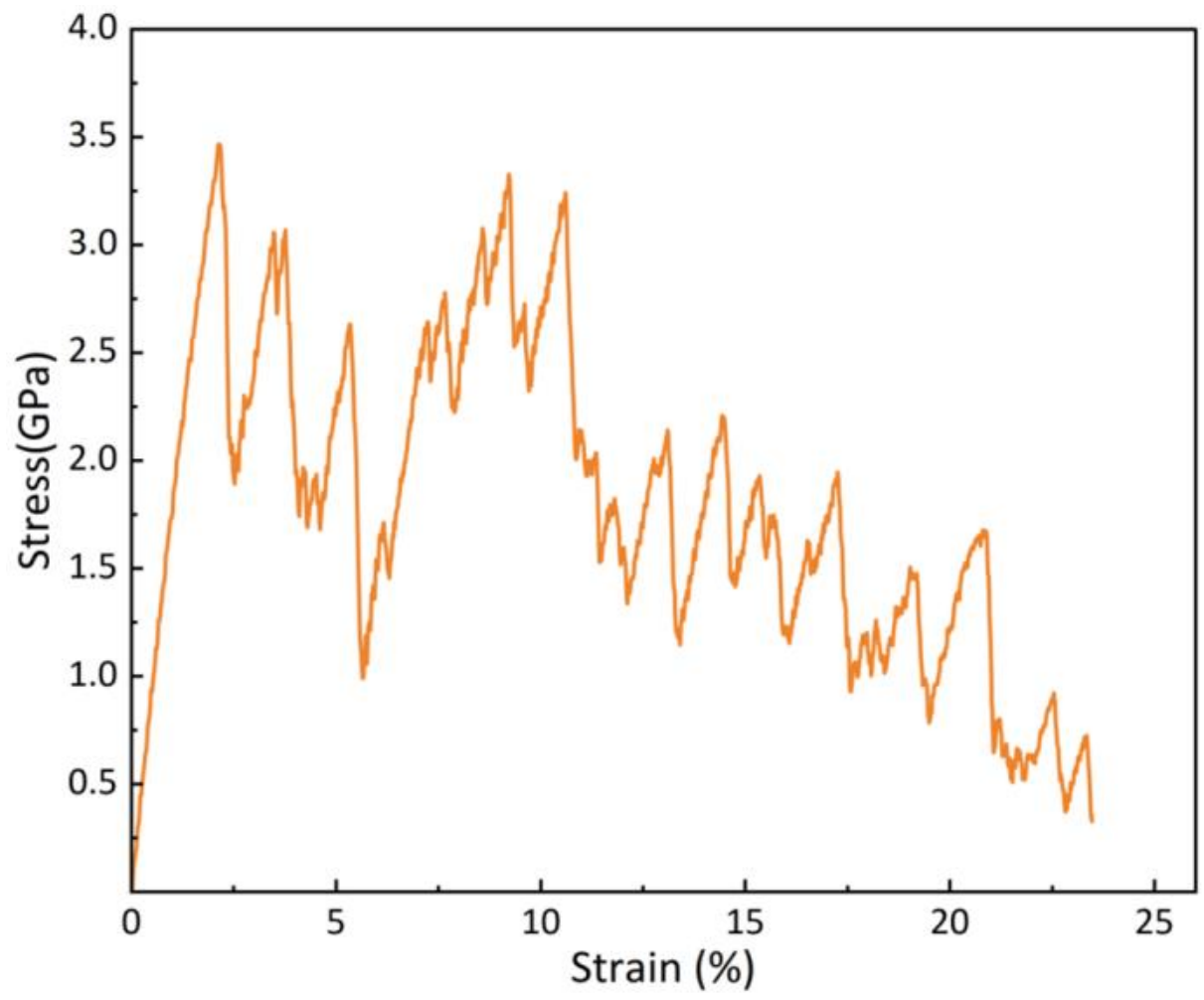

Fig. 9 | MD simulation of the stress-strain curve for NSC.

***Comment 10:** In the GSFE calculation, was the stress/strain effect included? The relationships between $\Delta\gamma_1$, $\Delta\gamma_2$, and $\Delta\gamma_3$ may change at different stresses.*

Response: Following the reviewer's suggestion, we have calculated the GSFE under 1% and 2% tensile strain, as shown in Supplementary Fig. 7. The results reveal that the GSFE is indeed stress/strain-dependent. With increasing strain, the GSFE of the MPEA decreases significantly, indicating a greater propensity for SF and twin formation. This finding is consistent with previous first-principles calculations on MPEAs [46]. In response to the reviewer's comments, we have added a short discussion in the revised manuscript and have also made corresponding revisions to the Methods section. Details as follows:

"In addition, we also calculated the GSFE curves with tensile strains of 1% and 2% (Supplementary Fig. 7), the result reveals a pronounced stress/strain dependence in the NSC MPEA. All GSFE parameters systematically decrease with increasing strain, and their distributions also exhibit significant overlap. This also suggests an enhanced propensity for separated dislocation and deformation twin formation. These findings are consistent with previous first-principles studies on MPEAs [46]."

"Uniaxial tensile strains of 1% and 2% were applied perpendicular to the primary {111} slip plane prior to the fault creation and NEB calculation, simulating a pre-deformed state. For each strain level (1%, 2%), a new set of 250 independent models was generated, and the GSFE calculation procedure described above was repeated. This approach allows for a systematic evaluation of how elastic strain influences the unstable SFE, intrinsic SFE, and the energy barriers associated with twinning."

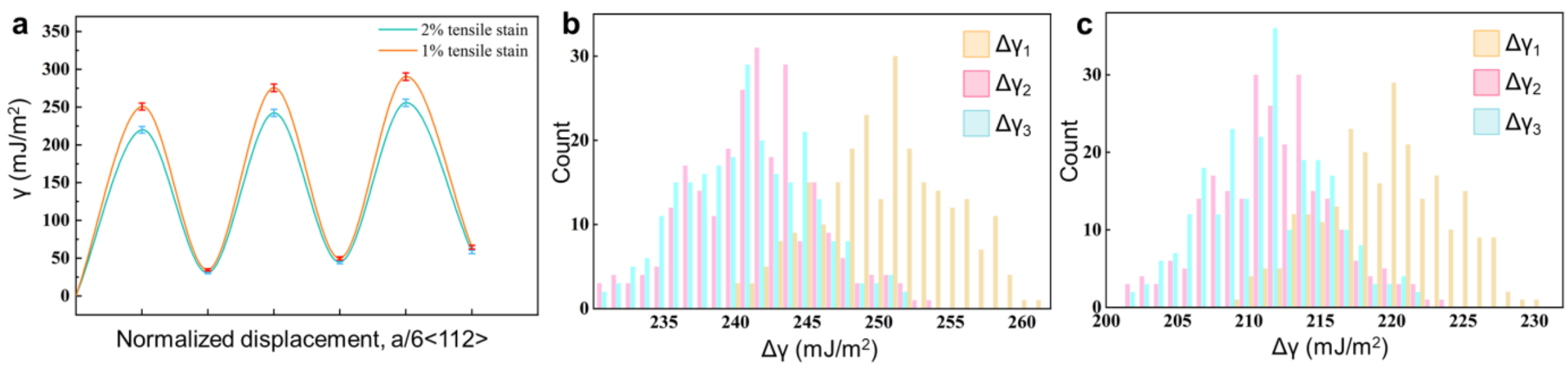

Fig. 7 | (a) GSFE curves under 1% and 2% tensile strains of the NSC. (b) Distribution of unstable SFE barriers under 1% tensile strains. (c) Distribution of unstable SFE barriers under 2% tensile strains.

***Comment 11:** What interatomic potential and parameters were used for the MD simulations?*

Response: Thanks for the reviewer's comment. We have added details of MD simulations in the Supplementary methods section. The details are as follows:

"Tensile deformation simulations. To unravel the underlying deformation mechanisms, MD simulations of the $Fe_{48}Mn_{32}Co_{10}Cr_{10}$ MPEA were performed using the Large-scale Atomic/Molecular Massively Parallel Simulator (LAMMPS) [2]. The simulation cell contained 510,528 atoms arranged in an orthogonal geometry with dimensions of 48 nm (length) × 16 nm (width) × 8.4 nm (thickness), as

shown in Supplementary Fig. 8. The crystallographic orientation of the model was aligned with the experimental system that show in Fig. 2. Atomic interactions were described using the Mie potential parameterized by Gröger et al. [3], which has been rigorously validated for capturing the effects of compositional randomness on defect properties in FCC high-entropy alloys, including stacking fault energies, point defect formation energies, and dislocation migration barriers. The simulations employed a time step of 1 fs. Free surfaces were imposed in all three dimensions to replicate the surface effects relevant to nanoscale specimens. The system was initially energy-minimized using the conjugate gradient algorithm, followed by a 200 ps equilibration in the NVT ensemble at 300 K to achieve thermodynamic stability before the onset of mechanical loading. Three atomic layers at the top and bottom of the model (along the length direction) were then fixed as rigid loading platens. Uniaxial tensile deformation was applied by imposing opposing velocities of v= ±0.024 Å/ps on the rigid layers, corresponding to an engineering strain rate of approximately 1.0 × 10^8 s^{-1}. The corresponding stress-strain curve is shown in Supplementary Fig. 9. The tensile simulation was conducted for 3 ns to adequately capture the progression of plastic deformation and the associated microstructural evolution. Microstructural evolution was visualized using the Open Visualization Tool (OVITO) [4]. The common neighbor analysis (CNA) method was employed to identify local crystal structures. Atoms were colored according to their identified structure: green for FCC, red for HCP, and white for disordered or interfacial atoms.”

Comment 12: *Strain rate of the MD tensile simulation was 10^8 s^{-1}. To reach 10% strain, it requires MD time in the order of miliseconds, which seems to be long for MD. Was any techniques for accelerated dynamics employed in the simulation? And at what temperature was the tensile MD performed?*

Response: Thanks for the reviewer’s comment. In our simulation, no accelerated dynamics were employed since the simulation time was only ~ 1 nanosecond (calculated according to the following formula):

$$\varepsilon = (\Delta L/L)/\Delta t \rightarrow \Delta t = (\Delta L/L)/\varepsilon = 10^{-1}/10^{8}\ \mathrm{s} = 10^{-9}\ \mathrm{s} = 1\ \mathrm{ns}$$

where ε represents strain rate. L and ΔL represent the length and elongation of the nanowire, respectively. Δt represents the simulation time. The temperature of the MD simulations conducted at ~ 300 K, the details of the MD simulation, and the temperature were added in the supplementary information and experimental methods section.

Comment 13: *There was no twin boundary observed in the tensile simulation although nucleation and growth were observed in the in situ TEM. Please explain. Is this caused by the time/size scale effect?*

Response: Thanks for the reviewer’s valuable comment. In initial simulations, we employed the second-nearest-neighbor modified embedded-atom method (2NN MEAM) potential developed by Choi [doi.org/10.1016/j.commatsci.2017.01.002]. This potential was parameterized based on binary systems such as Co-Cr and Co-Fe and extended to multi-component alloys like CoCrFeMnNi. Due to the complex

elemental distribution in MPEAs, it may not fully capture the complex characteristics of CoCrFeMnNi in the current study.

In response to the reviewer's constructive comments, we have carefully re-evaluated our simulation methodology and made modifications to the interatomic potential. We switched to the effective pair potential developed by Gröger [doi.org/10.1088/1361-651X/ab7f8b], which is specifically designed for fcc CoCrFeMnNi alloys. This potential uses a Lennard-Jones-type formalism with parameters derived from pure element properties and mixing rules for regular solutions, making it more suitable for capturing the properties of the MPEA. This change was motivated by the fact that the Cauchy pressure (C12-C44) in CoCrFeMnNi is small, indicating that pair interactions dominate the bonding—a feature better represented by a simple pair potential than by many-body methods like 2NN MEAM. In response to the reviewer's comments, we have revised the Supplementary MD section and replaced Supplementary Fig. 8 with new results. The details are as follows:

"Tensile deformation simulations. To unravel the underlying deformation mechanisms, MD simulations of the $Fe_{48}Mn_{32}Co_{10}Cr_{10}$ MPEA were performed using the Large-scale Atomic/Molecular Massively Parallel Simulator (LAMMPS) [2]. The simulation cell contained 510,528 atoms arranged in an orthogonal geometry with dimensions of 48 nm (length) × 16 nm (width) × 8.4 nm (thickness), as shown in Supplementary Fig. 8. The crystallographic orientation of the model was aligned with the experimental system that show in Fig. 2. Atomic interactions were described using the Mie potential parameterized by Gröger et al. [3], which has been rigorously validated for capturing the effects of compositional randomness on defect properties in FCC high-entropy alloys, including stacking fault energies, point defect formation energies, and dislocation migration barriers. The simulations employed a time step of 1 fs. Free surfaces were imposed in all three dimensions to replicate the surface effects relevant to nanoscale specimens. The system was initially energy-minimized using the conjugate gradient algorithm, followed by a 200 ps equilibration in the NVT ensemble at 300 K to achieve thermodynamic stability before the onset of mechanical loading. Three atomic layers at the top and bottom of the model (along the length direction) were then fixed as rigid loading platens. Uniaxial tensile deformation was applied by imposing opposing velocities of v= ±0.024 Å/ps on the rigid layers, corresponding to an engineering strain rate of approximately 1.0×10^8 s^{-1}. The corresponding stress-strain curve is shown in Supplementary Fig. 9. The tensile simulation was conducted for 3 ns to adequately capture the progression of plastic deformation and the associated microstructural evolution. Microstructural evolution was visualized using the Open Visualization Tool (OVITO) [4]. The common neighbor analysis (CNA) method was employed to identify local crystal structures. Atoms were colored according to their identified structure: green for FCC, red for HCP, and white for disordered or interfacial atoms."

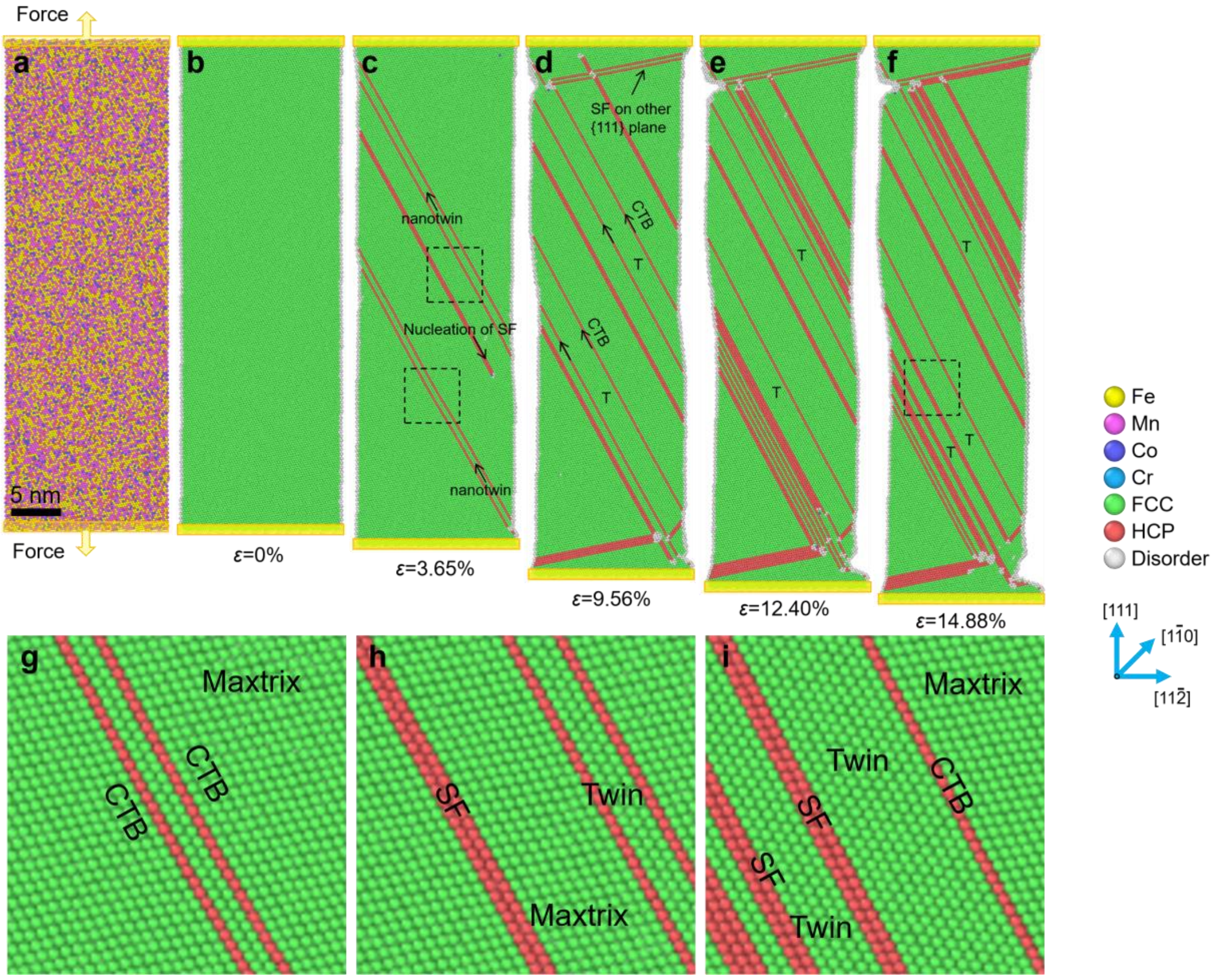

Fig. 8 | The molecular dynamics simulation of $Fe_{48}Mn_{32}Co_{10}Cr_{10}$ MPEA during in-situ tensile deformation. (a) After model relaxation, the distribution of the Fe, Mn, Co, and Co elements. (b) to (f) The tensile process from 0% to 14.88% strain is displayed. (g) to (i) Magnified details of SF and CTB. Green atoms represent the face-centered cubic structure. Red atoms represent the hexagonal close-packed structure, which can be considered as SFs formed by the emission of dislocations. During deformation, dislocations are emitted on different {111} planes, leading to the microstructure of non-adjacent SFs. (g) to (f) Enlarged image of the area outlined in red in c to e, revealing details of CTB and SF.

Reviewer #3:

I co-reviewed this manuscript with one of the reviewers who provided the listed reports. This is part of the Nature Communications initiative to facilitate training in peer review and to provide appropriate recognition for Early Career Researchers who co-review manuscripts.

Response: We acknowledge the reviewer's contribution and thank them for their valuable input on our manuscript.

Reviewer #4:

General comments: *Excellent work. The reviewer is in general supporting its publication.*

Several suggestions/comments are listed below for the consideration of the authors.

Response: We are grateful for the reviewer's immensely positive comments.

Comment 1: *Is the mixing entropy playing a role here at all? This has to be verified from ternary or binary alloys which also show similar negative enthalpy. Here in this work, pure metals are used as the benchmark. Fine, but it shall also include simple alloys.*

Response: Thanks to the reviewer for this valuable comment. We have conducted a substantial number of calculations and simulations to investigate the role of entropy and enthalpy in the deformation mechanisms of MPEAs. From a thermodynamic perspective, both the entropy and enthalpy are crucial for improving the mechanical properties of MPEAs. According to the Gibbs free energy formula $\Delta G = \Delta H - T\Delta S$, decreasing enthalpy ($\Delta H$) or increasing mixing entropy (ΔS) can lower the system's Gibbs free energy, making MPEAs thermodynamically stable. We also notice that entropy and enthalpy are coupled thermodynamic parameters. Adjusting the configurational entropy through compositional variation inevitably alters the mixing enthalpy, making it very difficult to isolate the effect of entropy or enthalpy alone. Based on our extensive MD simulations, a comprehensive literature review, and in alignment with the classical frameworks established by Yeh and Cantor [31, 32], we believe that entropy primarily functions to stabilize and promote single-phase random solid solutions. In other words, it renders the elemental distribution more homogeneous and randomized at the atomic scale. Enthalpy, on the other hand, governs the formation of ordered structures. This means that elements with negative mixing enthalpy are more likely to cluster together to form an ordered structure. We also consulted many senior experts in the field of MPEAs and metallic glasses on this issue, and they all agreed with this viewpoint.

Following the reviewer's suggestion, we also systematically examined binary and ternary alloys with different values of mixing entropy and enthalpy via MD simulations, as presented in Supplementary Figs. 10~15. Due to the increase in the number of elements, the enthalpy and entropy inevitably change simultaneously, making it very difficult to isolate the effect of entropy and enthalpy alone. Our results further support the above viewpoint: increasing entropy appears to render the elemental distribution more homogeneous and randomized, which leads to the SFE distribution becoming further apart, thereby reducing the overlap between $\Delta\gamma 1$ and $\Delta\gamma 2/3$. While decreasing enthalpy appears to render the elemental distribution inhomogeneous, leading to a non-uniform distribution of the SFE and thereby increasing the overlap between $\Delta\gamma_1$ and $\Delta\gamma_{2/3}$. However, we must acknowledge that our simulations encountered several limitations because direct comparison between alloys with different chemical elements is remarkably complicated. First, the reduced mixing entropy in binary and ternary systems often fails to sustain single-phase FCC solid solutions. This frequently results in mixed microstructures comprising FCC, BCC, and HCP phases. Second, the calculated SFEs are highly sensitive to the choice of interatomic potentials. A definitive, quantitative disentanglement of entropy and enthalpy contributions would require more extensive and systematic investigations.

In response to the reviewer's valuable comment, we have added the results of binary and ternary alloys in the Supplementary materials and added a short discussion in the Supplementary text, as follows:

"From a thermodynamic perspective, it should be emphasized that we do not deny the role of entropy in strengthening MPEAs, but rather that enthalpy is also crucial for improving their mechanical properties. According to the Gibbs free energy formula $\Delta G = \Delta H - T\Delta S$, decreasing enthalpy or increasing mixing entropy can lower the system's Gibbs free energy, making MPEAs thermodynamically stable. We performed GSFE molecular dynamics simulations on binary and ternary alloys with varying mixed entropy and enthalpy, as shown in Supplementary Figs. 10~15. On the one hand, reducing ΔH promotes element segregation/inhomogeneity and the formation of phases or intermetallic compounds. In MPEAs, regulating enthalpy to a suitable value is necessary to achieve both high strength and large uniform elongation (as shown in this study). Excessively negative enthalpy promotes the formation of intermetallic compounds, leading to high strength but poor plasticity. Conversely, high (positive) enthalpy provides only a limited strengthening effect. On the other hand, increasing entropy drives homogeneous distribution of alloy atoms and suppresses element clustering and the formation of independent phases or intermetallic compounds. High entropy drives uniform mixing of elements with different atomic radii, resulting in lattice distortion and solid solution strengthening, thereby also improving the hardness and strength of MPEAs [44]. Therefore, the excellent properties of MPEAs originate from the synergistic effect of enthalpy and entropy, rather than a single factor."

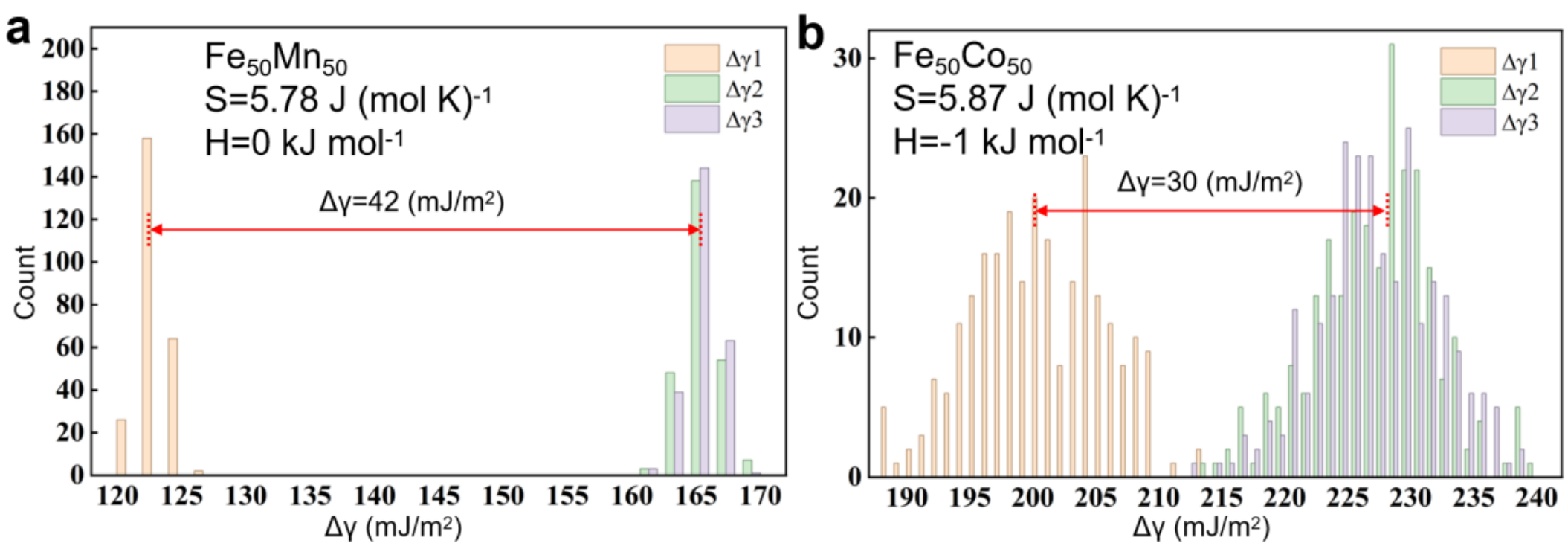

Fig. 10 | Distribution of unstable SFE barriers for (a) $Fe_{50}Mn_{50}$, (b) $Fe_{50}Co_{50}$. While the entropy remains constant, the decrease in enthalpy drives the peaks of the SFE distribution further apart.

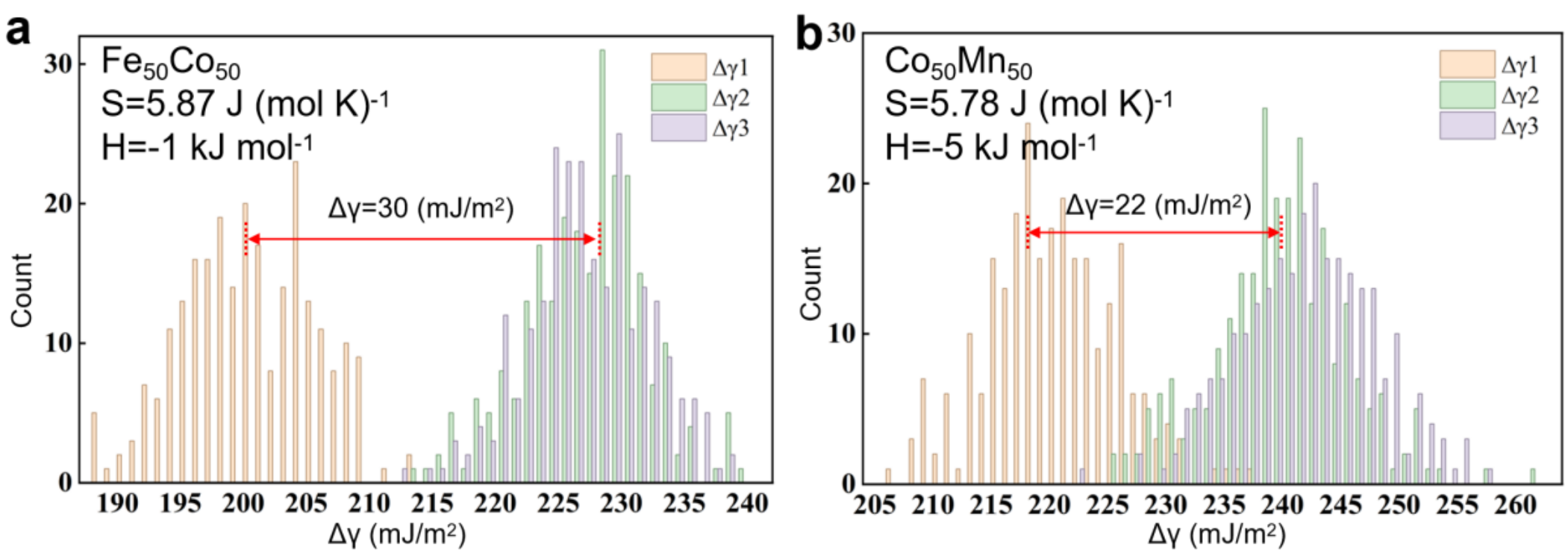

Fig. 11 | Distribution of unstable SFE barriers for (a) $Fe_{50}Co_{50}$, (b) $Co_{50}Mn_{50}$. While the entropy remains constant, the decrease in enthalpy drives the peaks of the SFE distribution further apart.

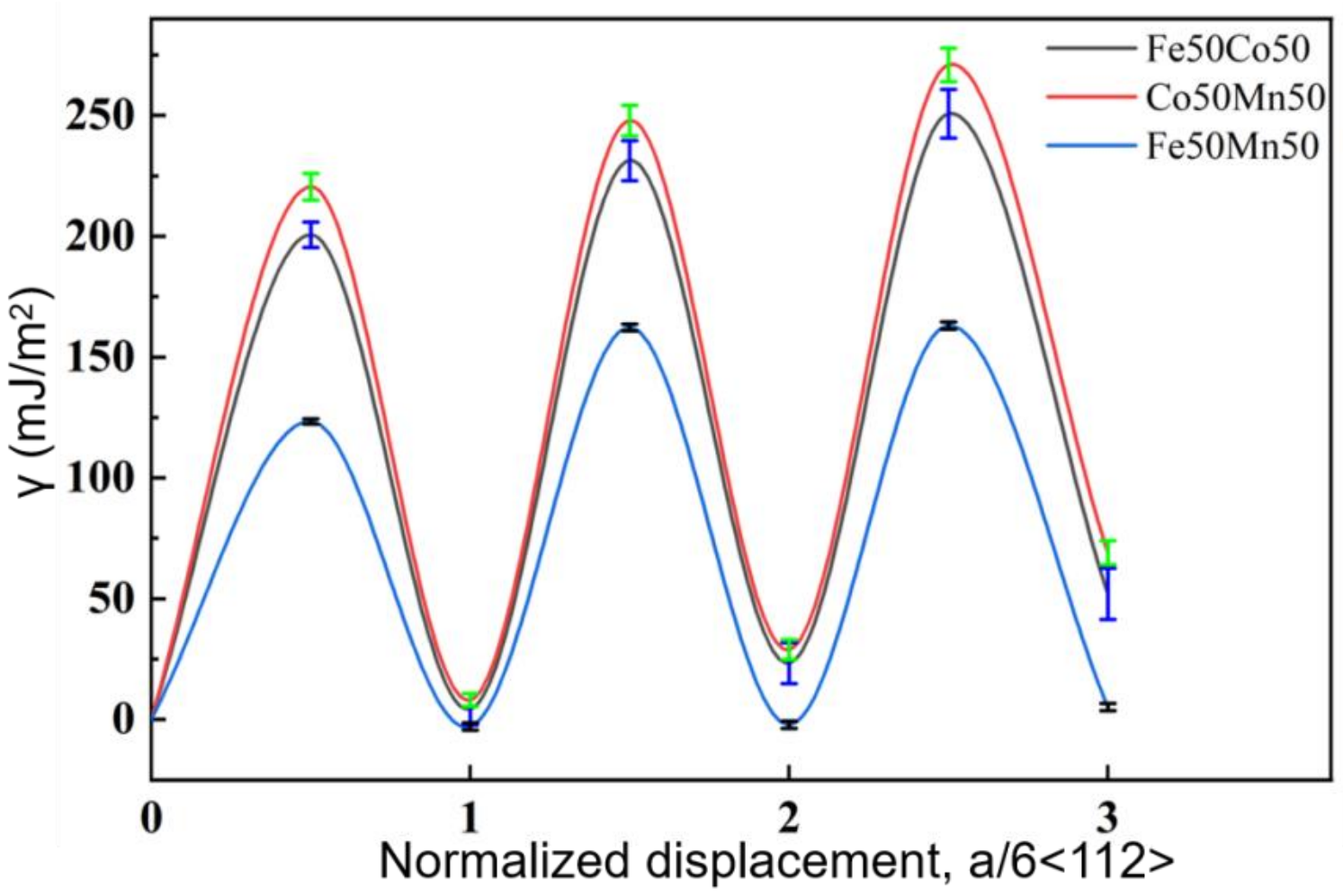

Fig. 12 | GSFE curves of the $Fe_{50}Mn_{50}$, $Co_{50}Mn_{50}$ and $Fe_{50}Mn_{50}$ along the twin formation pathway.

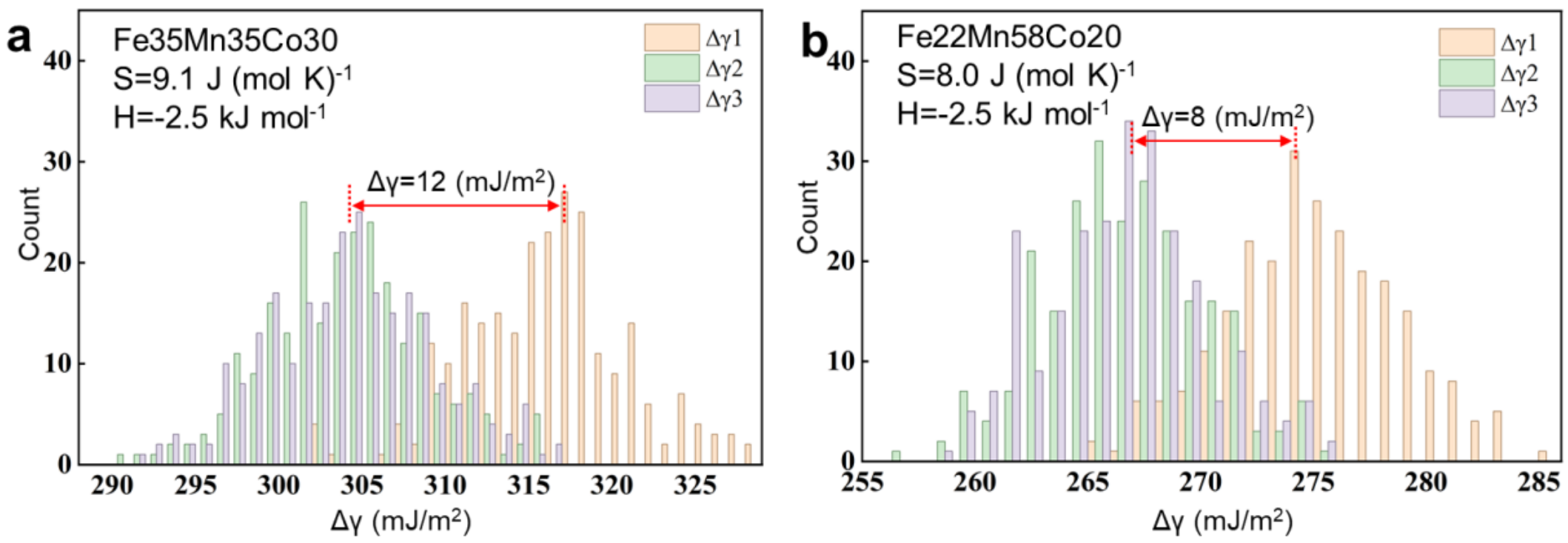

Fig. 13 | Distribution of unstable SFE barriers for (a) $Fe_{35}Mn_{35}Co_{30}$, (b) $Fe_{22}Mn_{58}Co_{20}$. While the enthalpy remains constant, the increase in entropy drives the peaks of the SFE distribution further apart.

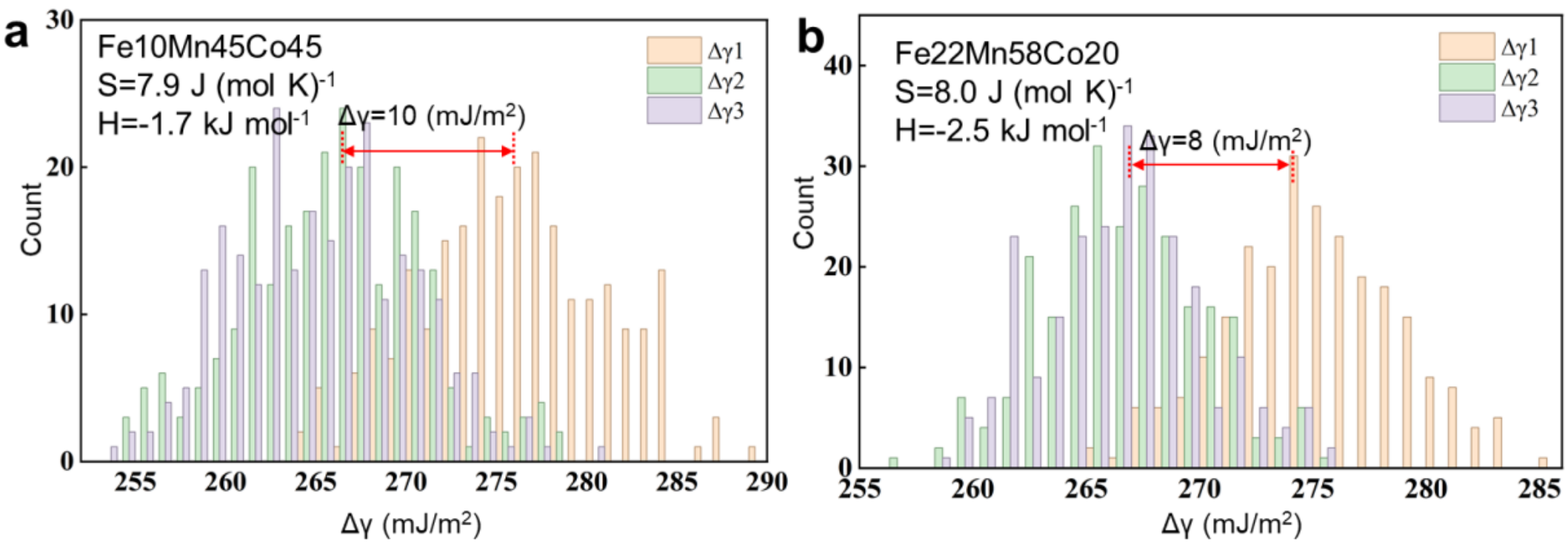

Fig. 14 | Distribution of unstable SFE barriers for (a) $Fe_{10}Mn_{45}Co_{45}$, (b) $Fe_{22}Mn_{58}Co_{20}$. While the enthalpy remains constant, the increase in entropy drives the peaks of the SFE distribution further apart.

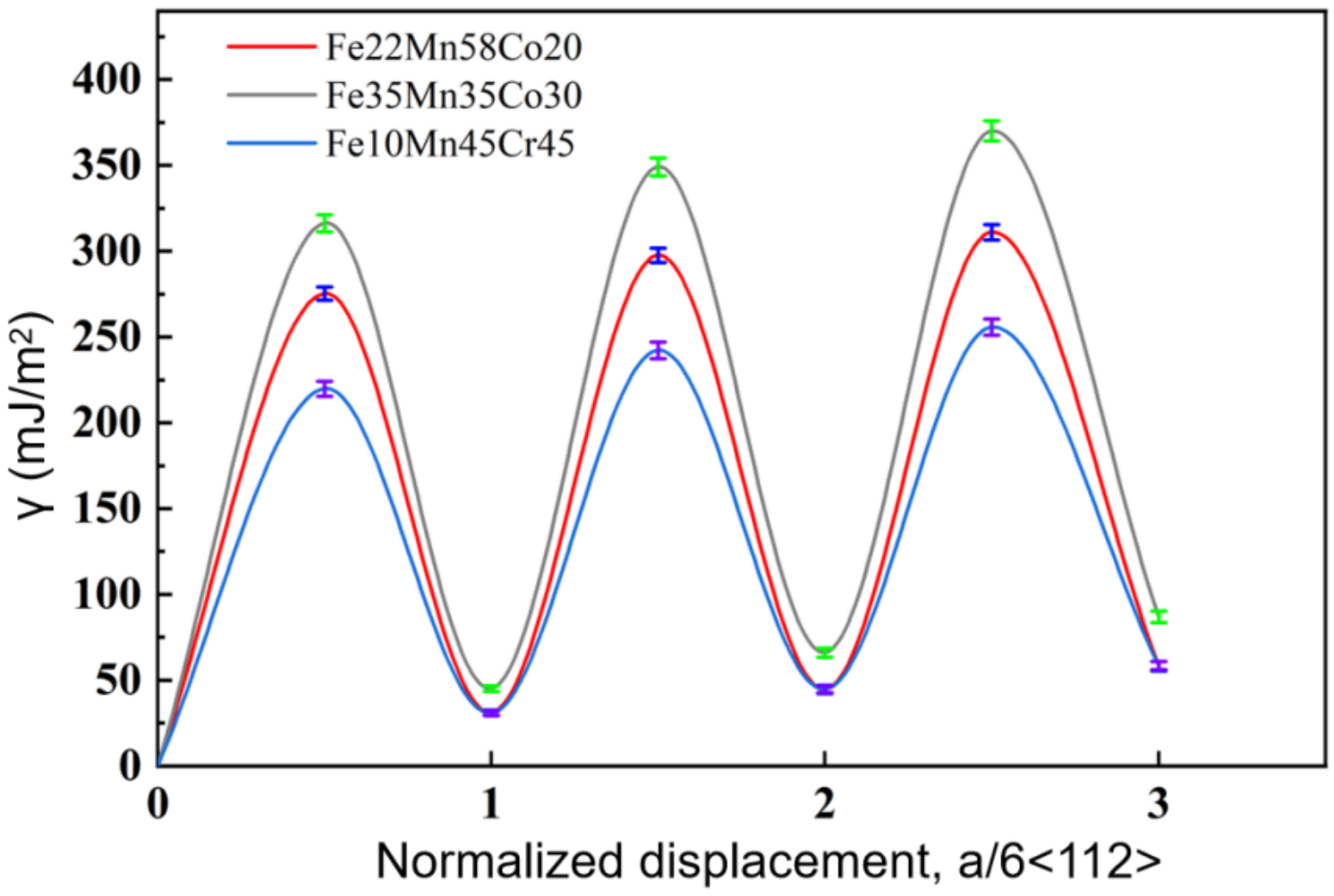

Fig. 15 | GSFE curves of the $Fe_{10}Mn_{45}Co_{45}$, $Fe_{22}Mn_{58}Co_{20}$ and $Fe_{35}Mn_{35}Co_{30}$ along the twin formation pathway.

Comment 2: *Why has it to be a single crystal? What happens to a nanosized polycrystalline material? Will the grain orientation for the single crystal change the mechanical response?*

Response: We appreciate the reviewer's comments.

1)Why has it to be a single crystal?

First, the single crystal is a typical model material system. However, most previous studies have focused on bulk polycrystalline MPEAs (containing numerous GBs) or relatively large sized samples, with limited research on the mechanical behavior of NSC MPEAs. Investigating NSC MPEAs can provide essential experimental data for advancing the theoretical framework of material mechanics. In addition, NSCs exhibit excellent mechanical properties such as high strength and hardness, but usually suffer from poor plasticity. Revealing the atomic-scale deformation mechanisms of NSC MPEAs can provide valuable guidance for designing high-performance nanosized metals. Furthermore, using single crystals eliminates the complicating effects of GBs. In nanosized polycrystalline metals, GBs usually significantly influence dislocation and twinning activities and frequently introduce GB plasticity (e.g., GB sliding, grain rotation, and GB migration). Thus, it is always difficult to distinguish the GB effect from chemical inhomogeneity. By employing single crystal samples, we can exclude the GB effect and reveal that elements with negative mixing enthalpy tend to cluster together, resulting in chemical inhomogeneity that significantly affects the mechanical behavior of MPEAs.

2) What happens to a nanosized polycrystalline material?

For nanosized polycrystalline MPEAs, we note that relevant research is very limited. Valuable inferences can be drawn from previous studies on nanosized polycrystalline pure metals. Based on these studies, for nanosized polycrystalline metals with grain sizes above ~50 nm, deformation is dominated by intragranular dislocation slip and twinning, with GBs serving as dislocation sources. When the grain size becomes sufficiently small (e.g., ~15 nm), GB plasticity (such as GB sliding, grain rotation, and GB migration)

becomes the dominant deformation mechanism [67]. Thus, it is reasonable to infer that nanosized polycrystalline MPEAs should exhibit similar trends: for those with relatively large grain sizes, deformation should be dominated by intragranular dislocation slip and twinning; for those with very small grain sizes, GB plasticity will become the dominant mechanism [68, 69]. However, because intragranular and intergranular deformation mechanisms can be significantly affected by both GBs and chemical inhomogeneity, the activation of dislocation sources, twinning behavior, and the transition to GB plasticity should differ substantially from those observed in pure metals.

3) Will the grain orientation for the single crystal change the mechanical response?

We have also conducted tensile tests with loading directions along <110> and <001> (Supplementary Figs. 16 and 17). The results show that grain orientation does influence the macroscopic mechanical response, such as uniform elongation, which reached ~28.0% and ~24.6% for loading along <110> and <001>, respectively. We notice that the loading direction can affect the slip systems, thus leading to various uniform elongations. However, the fundamental deformation mechanisms remain consistent across different orientations: the deformation is governed by the random generation of full dislocations, partial dislocations, and nanotwins, regardless of loading direction. Therefore, while orientation can affect certain aspects of the mechanical behavior, it does not alter the intrinsic deformation mechanisms in these nanoscale MPEAs.

In response to the reviewer's comments, we have added some sentences to the Methods part and a short discussion in the revised manuscript, as follows:

"Furthermore, we also notice that most previous studies have focused on bulk polycrystalline MPEAs (containing numerous GBs) or relatively large samples; research on the mechanical behavior of NSC MPEAs remains scarce. Finally, nanoscale single crystals without GBs eliminate the complicating effects of GBs, which helps to more accurately investigate the effect of negative mixing enthalpy on the mechanical behavior of MPEAs."

"Second, grain size also influences deformation mechanisms. Based on previous studies, as grain size decreases to a critical value (e.g., ~15 nm), the deformation of nanosized polycrystalline metals transitions from intragranular activities (dislocation slip and twinning) to intergranular mechanisms (grain boundary (GB) sliding, migration, and grain rotation) [67]. Thus, it is reasonable to infer that nanosized polycrystalline MPEAs should exhibit similar trends. In addition, both intragranular and intergranular deformation mechanisms can be significantly affected by GBs and chemical inhomogeneity; the dislocation activities, twinning mechanisms, and GB plasticity in nanosized polycrystalline MPEAs should differ substantially from pure metals and NSC MPEAs [68, 69]. Third, the loading direction can affect the uniform elongation of metals. We also conduct tensile tests of NSC MPEAs with orientation of <110> and <001> (Supplementary Figs. 16 and 17), the results indicate that loading orientation can influence macroscopic mechanical response, such as uniform elongation. However, the underlying deformation mechanisms—governed by the random activation of full

dislocations, partial dislocations, and nanotwins—remain consistent across different orientations. This suggests that while orientation affects certain mechanical properties, it does not change the intrinsic deformation behavior of nanoscale MPEAs, which is primarily determined by their chemical inhomogeneity."

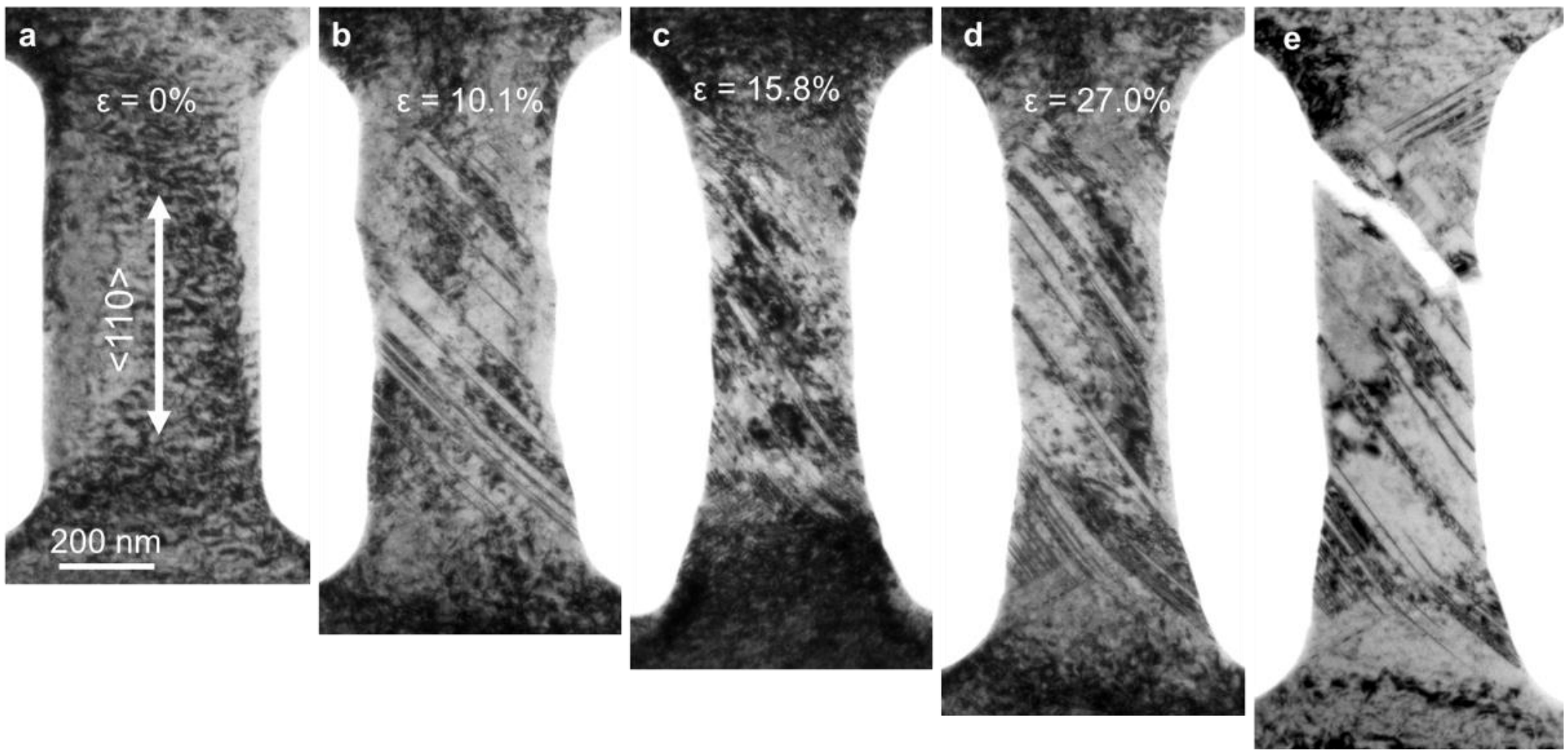

Fig. 16 | Bright-field TEM images of the NSC in the <110> direction during in-situ tensile deformation.

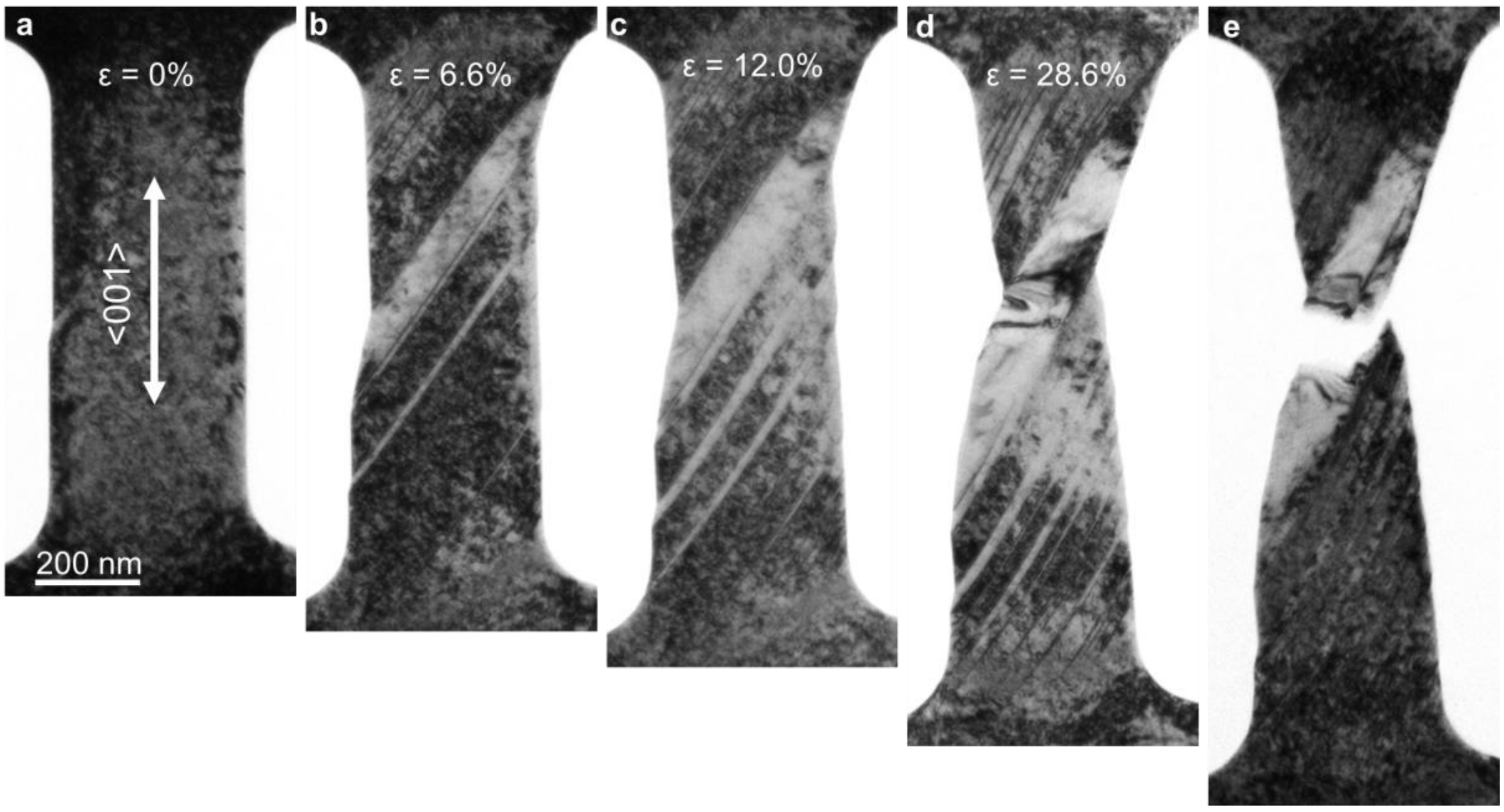

Fig. 17 | Bright-field TEM images of the NSC in the <001> direction during in-situ tensile deformation.

Comment 3: *Any particular reason for the choice of $Fe_{48}Mn_{32}Co_{10}Cr_{10}$? Is SFE or CRSS ever considered during the material selection?*

Response: Thanks for the reviewer's comment. We selected $Fe_{48}Mn_{32}Co_{10}Cr_{10}$ based on three primary considerations. First, $Fe_{48}Mn_{32}Co_{10}Cr_{10}$ is a well-known system with exceptional mechanical properties that have attracted intensive interest [9]. Second, this alloy is characterized by negative enthalpy, leading to a wide range of SFE values due to local chemical fluctuations. Third, the elements Fe, Mn, Co, and Cr have very similar metallic radii, so the effect of elastic strain energy (due to atomic size mismatch) on enthalpy can be neglected. For this reason, $Fe_{48}Mn_{32}Co_{10}Cr_{10}$ NSC is an ideal system for investigating the effect of

negative enthalpy-induced local chemical fluctuations on the mechanical behavior of multi-principal element alloys. In response to the reviewer's comments, several sentences were added in the Methods part of the revised manuscript, as follows:

"The $Fe_{48}Mn_{32}Co_{10}Cr_{10}$ NSC was selected as the model system. This is a well-known system with exceptional mechanical properties that has attracted intense interest [9]. In addition, it is suitable for investigating how negative-enthalpy-induced local chemical fluctuations influence the mechanical behavior of multi-principal element alloys. This system exhibits negative enthalpy and covers a wide range of SFE values. Moreover, the metallic radii of Fe, Mn, Co, and Cr are very similar, allowing the elastic strain energy contribution to enthalpy to be neglected. Such a design enables the isolated examination of enthalpy effects on the deformation behavior of nanosized single-crystal MPEAs."

Comment 4: *Regarding uniform plasticity: hardening and softening are balanced all the way. The hardening part is nicely explained, but the softening part is not, which is certainly needed to account for this balance.*

Response: Thanks for the reviewer's comment. We have added a detailed discussion on the competing strain-hardening and softening mechanisms and their evolution during deformation, which explains the balance observed at the plateau stage and the eventual necking. In response to the reviewer's constructive comments, several sentences were added in the revised manuscript, as follows:

"In addition, chemical inhomogeneity—specifically, mixing of elements with different atomic radii resulting in lattice distortion—and solid solution strengthening effects also contribute to strain hardening [44]. Besides strain hardening, several factors lead to softening. First, large free surfaces allow numerous nucleated dislocations to easily escape. Second, many partial dislocations nucleate and glide parallel to the TBs, leading to TB migration. The synergy of these mechanisms results in a balance between strain hardening and softening, as observed in the flat plateau of the strain-hardening rate curve (Supplementary Fig. 4). In the later stage of deformation, those regions that can easily nucleate dislocations become exhausted, and strain is then accommodated by dislocation nucleation elsewhere at sites with very high energy barriers. As a result, stress concentrations readily occur and the balance between strain hardening and softening is disturbed. Consequently, the strain-hardening rate decreases to values below 1.0, and the NSC can no longer effectively disperse stress through uniform plastic deformation; instead, it becomes prone to non-uniform deformation in weak areas, leading to necking. This mechanism completely explains the plateau feature of the strain-hardening rate curve and the final instability behavior of the NSC MPEA."

Reply to the Reviewers

We thank the reviewers for their in-depth review. The constructive suggestions provided have been considered to substantially improve the paper. A point-by-point response is included below, in which the reviewer's comments (in italic font) are first repeated and then our responses follow. The changes made in the revised manuscript are indicated by black bold font.

Reviewer #5:

General comments: This is not a full review of the manuscript, but mostly reviewing response to the first reviewer comments. I carefully reviewed the response letter and revised materials specifically regarding the issues of (1) the interpretation of necking and stress-strain behavior, and (2) the reliability of the atomistic simulations.

Response: We thank the reviewer for the careful evaluation of our revised manuscript and for the constructive comments.

Comment 1: Regarding the discussion of necking and the stress-strain curves, I do not believe the authors have fully resolved the original concern. Necking is generally identified from the engineering stress-strain response when the slope transitions from positive to negative. Although the material in Fig. 2b exhibits limited strain hardening, necking may still occur under such conditions. However, the explanation provided by the authors is not fully convincing because the presented "true stress-strain" curves still resemble engineering stress-strain behavior. In a true stress-strain response, the stress is generally expected to continue increasing after the onset of necking due to the reduction in cross-sectional area. In contrast, Supplementary Fig. 4 still shows a decrease in stress after the proposed necking point.

In addition, the reported change in strain rate from 1.5% to 1% is relatively small and, considering the typical variability in experimental measurements, does not appear sufficient to conclusively support the authors' interpretation. The TEM image in Fig. 2a does suggest some reduction in cross-sectional area; however, in my opinion, the evidence is still insufficient to unambiguously establish necking.

Response: We thank the reviewer for this constructive comment. We fully agree with the reviewer's criterion (well-established) for necking identification: the transition from positive to negative slope in the engineering stress-strain curve. In addition, according to the Considère criterion [1], necking can also be determined by identifying the intersection point between the strain hardening rate and the true strain-stress curve, or by identifying the point where the normalized strain hardening rate (the ratio of strain hardening rate and true stress) falls below 1.0 (100%). As the reviewer pointed out, variability in experimental measurements makes it difficult to accurately identify the necking point. Thus, we carefully evaluated the onset of necking/localized deformation by identifying the point at which the slope transitions from positive to negative in the engineering stress-strain curve, identifying the intersection point between the true stress-strain curve and the strain-hardening rate (Supplementary Fig. 4b and 4c), and determining the point where the normalized strain-hardening rate drops below 100% (Supplementary Fig. 4d).

In addition, we thank the reviewer for pointing out that the true stress-strain curve in Supplementary Fig. 4b is different from the conventional true stress-strain response observed in bulk materials ("the stress is generally expected to continue increasing after the onset of necking due to the reduction in cross-sectional area"). Inspired by the reviewer's constructive comments, we have carefully re-examined our in situ TEM results. We observed that after the true strain exceeds ~17%, the failure mode of the NSC proceeds through rapid local shear followed by fracture, rather than conventional necking (Supplementary Fig. 5). This rapid local shear and subsequent fracture may contribute to the atypical true stress-strain response observed in the NSC. Because the true stress is calculated according to the equation $\sigma_T=\sigma_N\times(1+\varepsilon_N)$, the effect of local cross-sectional reduction may not be reflected in the same manner as that typically observed in bulk materials. This may explain why the true stress-strain response of the NSC differs from that typically observed in bulk materials.

Following the reviewer's constructive suggestion and considering the variability in experimental measurements, we carefully re-evaluated the onset of necking/localized deformation using a combination of the three methods mentioned above together with the in situ TEM observations, and revised the manuscript accordingly.

"Uniaxial tensile loading was applied along the <111> direction perpendicular to the TEM beam direction, resulting in three distinct stages: (a) uniform plastic deformation, (b) stress concentration and rapid local shear, and (c) crack initiation, propagation, and fracture."

"Based on the engineering/true strain-stress curve, strain-hardening rate curve (Supplementary Fig. 4) and in situ TEM images (Supplementary Fig. 5), after the true strain exceeds ~ 17%, the NSC fractures rapidly through local shear, followed by crack initiation and propagation. At the early stage (true strain below 5%), the NSC exhibits strong strain hardening (Supplementary Fig. 4d). As the true strain increases to the range of 5-17%, the normalized strain-hardening rate remains at ~1.5 (150%). This relatively stable normalized strain-hardening rate ensures the relatively large uniform elongation of the NSC. After the true strain exceeds ~17%, the normalized strain-hardening rate falls below 1.0 (100%) and drops sharply. At this point, the readily mobile dislocations have been exhausted, and further deformation becomes increasingly localized in high-stress regions, where dislocation activities with much higher energy barriers are required. Consequently, the NSC can no longer disperse stress through uniform plastic deformation and instead undergoes rapid local shear followed by crack initiation and propagation (Supplementary Fig. 5). This may explain why the true stress-strain response of the NSC differs from that typically observed in bulk materials."

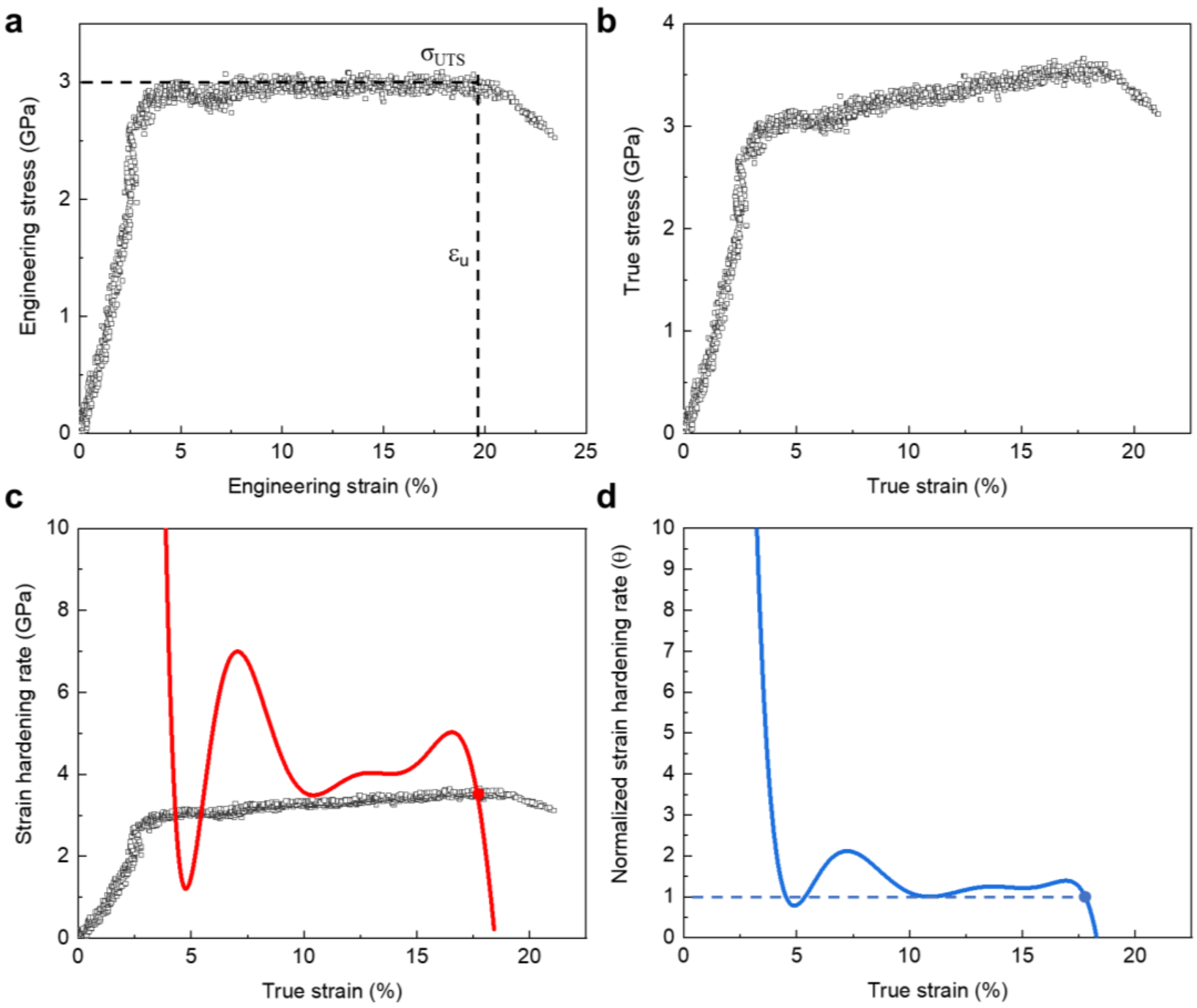

Fig. 4 | (a) Engineering stress-strain curve of the NSC; (b) True stress-strain curve of the NSC; (c) Strain hardening rate curve of the NSC; (d) Normalized strain-hardening rate curve of the NSC.

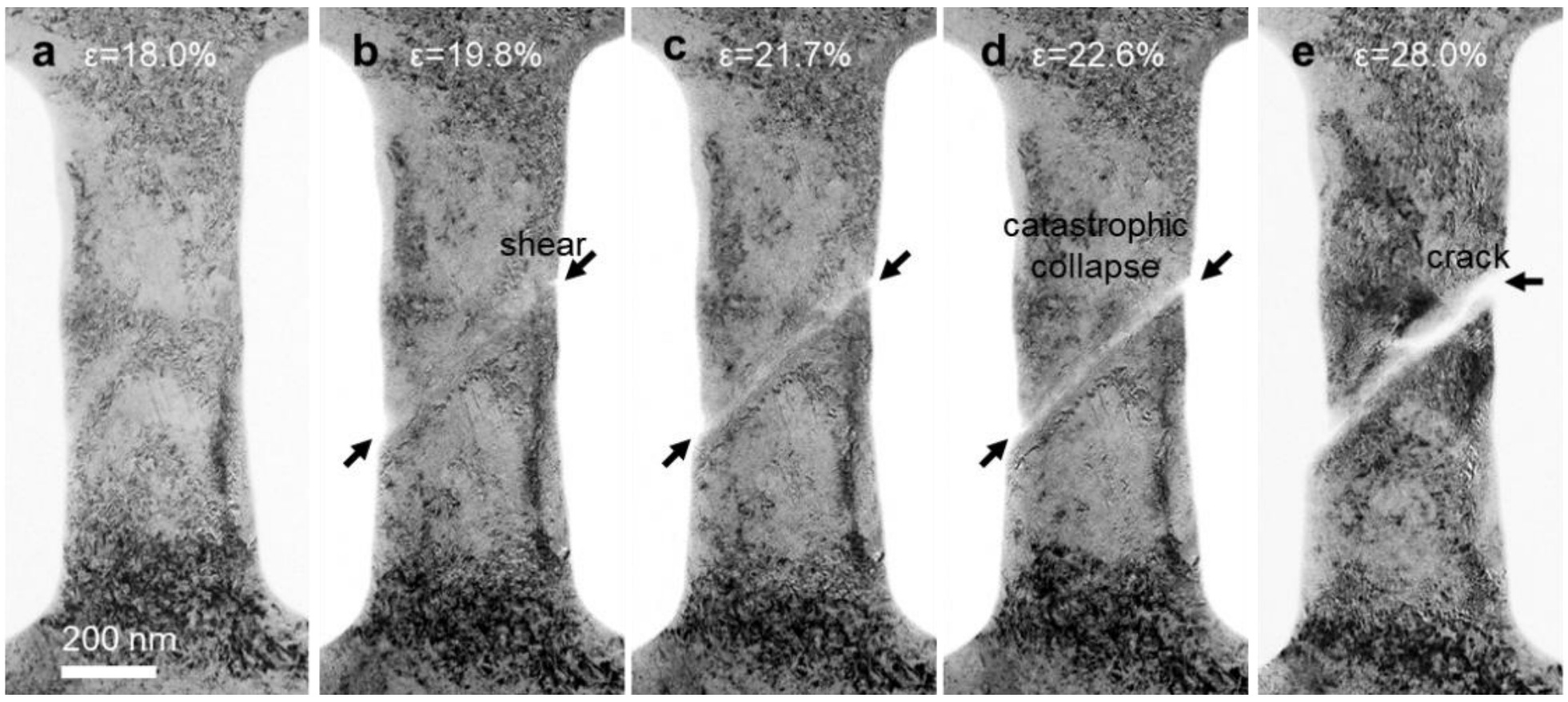

Fig. 5 | In situ TEM images revealing dislocation traversal and the resulting shear instability in the NSC sample before fracture.

Comment 2: Regarding the atomistic simulations, I understand the difficulty of obtaining accurate many-body interatomic potentials for this alloy system and appreciate that the authors employed an available potential based on a Lennard-Jones-type formalism. However, the manuscript still makes strong claims that (i) the observed trends are reliable and (ii) the simulations are consistent with experiments, without providing sufficient justification for these conclusions. I remain concerned that even qualitative trends may depend strongly on the choice of interatomic potential, and this limitation is not adequately addressed.

That said, I find that the additional methodological details provided for the NEB calculations are sufficient, and the explanation for why $\Delta\gamma_1$ is lower than $\Delta\gamma_2$ or $\Delta\gamma_3$ is well justified.

Response: We thank the reviewer for these constructive comments. We fully agree that, as the reviewer pointed out, even qualitative trends may depend strongly on the choice of interatomic potential. In addition, selecting an accurate interatomic potential for multi-principal element alloys remains highly challenging because of their complex chemical environments and many-body interactions.

In response to the reviewer's concern, we have removed the statements suggesting that "(i) the observed trends are reliable" and "(ii) the simulations are consistent with experiments" from the revised manuscript. We have also added a discussion to explicitly acknowledge the limitations of the present simulations and to avoid overinterpreting the MD results. The revised text is provided below:

"In addition, we calculated the GSFE curves under tensile strains of 1% and 2% using MD simulations (Supplementary Fig. 10). The results reveal a pronounced stress/strain dependence in the NSC MPEA. All GSFE parameters systematically decrease with increasing strain, and their distributions also exhibit significant overlap, which is consistent with previous calculations [47]. While it should be noted that, because the actual potential of MPEAs is extremely difficult to determine, the calculated values of GSFE may vary when using different potentials. Thus, the MD simulation may only represent a trend. In many cases, even the qualitative trends may depend strongly on the choice of interatomic potential."

[1] I.S. Yasnikov, A. Vinogradov, Y. Estrin, Revisiting the Considère criterion from the viewpoint of dislocation theory fundamentals, *Scripta Materialia*, 76, 2014, 37-40.
